# The first hyaenodont from the late Oligocene Nsungwe Formation of Tanzania: Paleoecological insights into the Paleogene-Neogene carnivore transition

**DOI:** 10.1371/journal.pone.0185301

**Published:** 2017-10-11

**Authors:** Matthew R. Borths, Nancy J. Stevens

**Affiliations:** 1 Department of Biomedical Sciences, Heritage College of Osteopathic Medicine, Ohio University, Athens, Ohio, United States of America; 2 Center for Ecology and Evolutionary Studies, Ohio University, Athens, Ohio, United States of America; Royal Belgian Institute of Natural Sciences, BELGIUM

## Abstract

Throughout the Paleogene, most terrestrial carnivore niches in Afro-Arabia were occupied by Hyaenodonta, an extinct lineage of placental mammals. By the end of the Miocene, terrestrial carnivore niches had shifted to members of Carnivora, a clade with Eurasian origins. The transition from a hyaenodont-carnivore fauna to a carnivoran-carnivore fauna coincides with other ecological changes in Afro-Arabia as tectonic conditions in the African Rift System altered climatic conditions and facilitated faunal exchange with Eurasia. Fossil bearing deposits in the Nsungwe Formation in southwestern Tanzania are precisely dated to ~25.2 Ma (late Oligocene), preserving a late Paleogene Afro-Arabian fauna on the brink of environmental transition, including the earliest fossil evidence of the split between Old World monkeys and apes. Here we describe a new hyaenodont from the Nsungwe Formation, *Pakakali rukwaensis* gen. et sp. nov., a bobcat-sized taxon known from a portion of the maxilla that preserves a deciduous third premolar and alveoli of dP^4^ and M^1^. The crown of dP^3^ bears an elongate parastyle and metastyle and a small, blade-like metacone. Based on alveolar morphology, the two more distal teeth successively increased in size and had relatively large protocones. Using a hyaenodont character-taxon matrix that includes deciduous dental characters, Bayesian phylogenetic methods resolve *Pakakali* within the clade Hyainailouroidea. A Bayesian biogeographic analysis of phylogenetic results resolve the *Pakakali* clade as Afro-Arabian in origin, demonstrating that this small carnivorous mammal was part of an endemic Afro-Arabian lineage that persisted into the Miocene. Notably, *Pakakali* is in the size range of carnivoran forms that arrived and began to diversify in the region by the early Miocene. The description of *Pakakali* is important for exploring hyaenodont ontogeny and potential influences of Afro-Arabian tectonic events upon mammalian evolution, providing a deep time perspective on the stability of terrestrial carnivore niches through time.

## Introduction

Carnivores occupy vital ecological roles in modern terrestrial ecosystems, stabilizing community structure and shaping patterns of biodiversity [1–5]. Today, terrestrial carnivore niches in Africa—and in most terrestrial ecosystems—are primarily occupied by species from the mammalian order Carnivora [6]. Yet carnivorans were absent from the African landscape for the first two-thirds of the Cenozoic [7]. Instead, carnivore niches in Afro-Arabia throughout the Paleogene were primarily occupied by species of the extinct mammalian clade Hyaenodonta [8, 9]. During the Paleogene, Afro-Arabia was relatively isolated from other landmasses [10, 11] and hyaenodonts featured as apex predators in a largely endemic fauna that included Afro-Arabian radiations of afrotherians [12], anthracotheres [13, 14], rodents [15, 16], and primates [17, 18]. Near the end of the Paleogene, the Arabian Peninsula closely approximated Eurasia, facilitating periodic faunal interchange between the African continent and Eurasia [19, 20]. The middle Miocene witnessed the development of the “Gomphothere landbridge”, and with it, progressive faunal exchange [21]. The Miocene fossil record of Afro-Arabia documents these events, providing evidence of a mixed carnivore fauna that includes Afro-Arabian hyaenodonts and Eurasian carnivorans in the same fossil localities [22–24]. For millions of years, hyaenodonts and carnivorans co-existed on the African landmass, until hyaenodonts became extinct near end of the Miocene [9], making way for the evolution of the modern African carnivore fauna [7]. During this interval, the African fauna underwent dramatic transformations as faunal exchange continued with Eurasia [25, 26] against the backdrop of landscape alteration influenced by tectonic activity as the East African Rift System developed [27] and seasonally drier and more open habitats emerged [28]. The changing carnivore fauna of the African early Neogene is a natural ecological experiment offering insights into potential impacts of rapid environmental and faunal shifts upon modern carnivore faunas.

Unfortunately, the earliest phases of the African carnivore faunal transition are not well documented. A substantial temporal gap in the fossil carnivore record of Afro-Arabia exists between the early Oligocene hyaenodonts found in the Fayum Depression of Egypt (~29.2 Ma), and the early Miocene hyaenodonts and carnivorans found in eastern and southern Africa (23–17 Ma) [18, 29]. Without higher-resolution paleontological sampling, it is difficult to unravel specific faunal impacts of the carnivoran invasion of Afro-Arabia. Work in the Rukwa Rift Basin (RRB) in the western arm of the East African Rift System (EARS) in southwestern Tanzania aims to address this temporal gap, providing a precisely dated glimpse of the Afro-Arabian fauna near the Paleogene-Neogene boundary [27, 30].

Here we describe the first carnivorous mammal documented from the late Oligocene of Africa south of the equator. This new taxon was discovered in a ~25.2 Ma locality in the Nsungwe Formation of the RRB, a temporal window that closely approximates estimates for the arrival of Carnivora on the African continent [31, 32]. The fragmentary specimen preserves key new insights into hyaenodont deciduous dentition. Recent work by Bastl et al. [33, 34, 35] built a foundational understanding of hyaenodont ontogeny in a phylogenetic context by focusing on the deciduous dentition of the Eurasian and North American genus *Hyaenodon*. With this specimen we are able to expand Bastl’s insights from *Hyaenodon* to Afro-Arabian hyaenodonts. We incorporate deciduous dental morphology into a phylogenetic and biogeographic analysis to place the new taxon within a systematic, temporal, and biogeographic context with other Afro-Arabian hyaenodonts. This discovery demonstrates there were hyaenodonts in the late Oligocene that closely overlapped in body size with early Miocene carnivores. Among carnivores, body size is a significant indicator of niche occupation [36]. This taxon is an important source of information on the faunal restructuring that followed the arrival of carnivorans on the African continent, a change in the carnivore guild that may have displaced hyaenodonts from previously occupied niches, and contributed to the eventual extinction of the first Afro-Arabian carnivores.

### Institutional abbreviations

AMNH, American Museum of Natural History, New York; DPC, Division of Fossil Primates, Duke Lemur Center, Duke University; BMNH, Natural History Museum, London; KNM/NMK, National Museums of Kenya, Nairobi, Kenya; MCZ, Museum of Comparative Zoology, Harvard University, Cambridge; MNHN, Muséum National d’Histoire Naturelle, Paris; RRBP, Rukwa Rift Basin Project (identifier used by the Tanzanian Antiquities Unit), Dar es Salaam, Tanzania.

## Materials and methods

### Geological context

**Fig 1:**
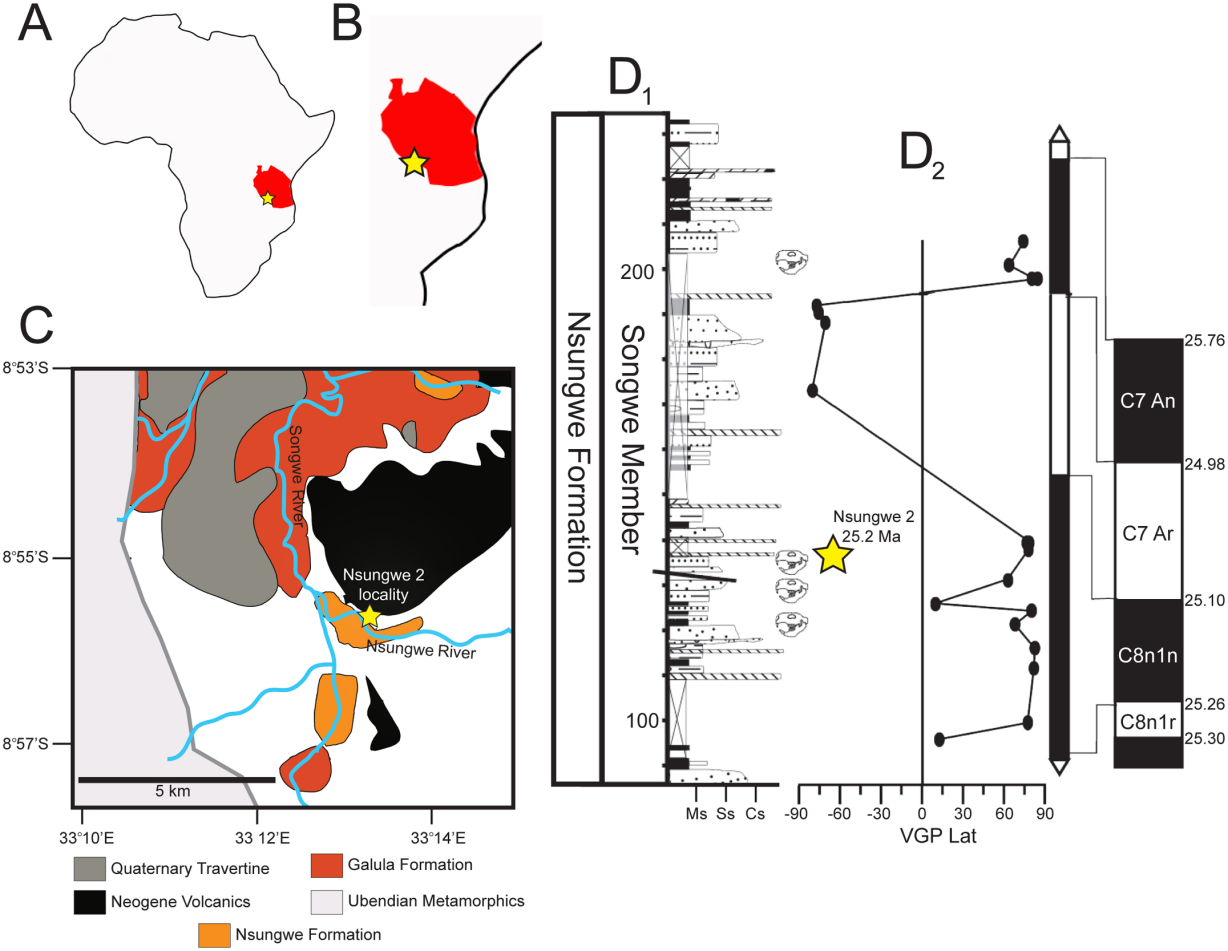
Geological context of Nsungwe 2 locality. Nsungwe 2 (yellow star in all portions of the figure) is a fossiliferous locality in the Songwe Member of the Nsungwe Formation in the Rukwa Rift Basin of southwestern Tanzania. **A**, The context of the Rukwa Rift Basin in Africa and **B**, in Tanzania. **C**, Geological map of the Nsungwe Formation and surrounding outcrop. **D**, Stratigraphic column of the fossiliferous section of the Songwe Member of the Nsungwe Formation with magnetostratigraphic correlations mapped through the section. Radiometric date of 25.2 Ma inferred from U-Pb zircon dating (see [27, 37]). **Ms**, Mudstone; **Ss**, Sandstone; **Cs**, Conglomerate; **Striped**, Volcanic tuff; **VGP lat**, virtual geomagnetic pole latitude; **Black**, normal polarity; **White**; reverse polarity.

The type specimen was excavated from the Nsungwe 2 locality in the late Oligocene Songwe Member of the Nsungwe Formation in the Rukwa Rift Basin of southwestern Tanzania (Fig 1). The Nsungwe Formation represents a continental rift-fill sequence containing several fossil bearing localities in horizons interpreted as sheet flood deposits within a small, flashy discharge fluvial system that appears to have drained into a local wetland [27, 30]. Nsungwe 2 is one of the richest localities in the Nsungwe Formation, and is dated to ~25.2 Ma based on biostratigraphy, dated ash beds, and detrital zircon geochronology [27, 30, 37]. Sedimentological data and the presence of aquatic and semi-aquatic taxa (e.g., fishes, frogs, and aquatic invertebrates), suggest habitats that experienced seasonal or periodic climatic fluctuations, with perennially available water sources [27, 30, 38, 39].

### Nomenclatural acts

The electronic edition of this article conforms to the requirements of the amended International Code of Zoological Nomenclature, and hence the new names contained herein are available under that Code from the electronic edition of this article. This published work and the nomenclatural acts it contains have been registered in ZooBank, the online registration system for the ICZN. The ZooBank LSIDs (Life Science Identifiers) can be resolved and the associated information viewed through any standard web browser by appending the LSID to the prefix "http://zoobank.org/". The LSID for this publication is: urn:lsid:zoobank.org:pub:B03A9CC0-B057-42AB-A7A0-DEDC3BBF16F0. The electronic edition of this work was published in a journal with an ISSN, and has been archived and is available from the following digital repositories: PubMed Central, LOCKSS.

### Permissions

All necessary permissions were obtained to undertake the described study, which complied with all relevant regulations. Fieldwork was conducted under permits issued by the Tanzanian Commission for Science and Technology (COSTECH), the Tanzania Antiquities Unit, and the Tanzanian Division of Immigration.

### Specimen preparation, measurement, and body mass estimation

After mechanical preparation by K. Whitman at the Ohio University Fossil Preparation and Imaging Facility, the specimen was micro-CT scanned in the Duke MicroCT lab in the Shared Materials Instrumentation Facility at Duke University, Raleigh, NC on a Nikon XTH 225 ST scanner. A digital model of the specimen is available as a 3D PDF in the supplementary materials for this study and a PLY digital model of the specimen is available for viewing and download in Project 303 on MorphoSource, a repository for 3D scan data supported by the NSF < http://www.morphosource.org/Detail/SpecimenDetail/Show/specimen_id/8140>. The specimen was scanned with a voxel size of 0.02003 mm in each dimension, at a voltage of 114 kV and amperage of 127 μa. The digital model was constructed and visualized using volume rendering and isosurface rendering in Avizo 8.0.

Dental nomenclature used in this study follows Holroyd [40] and Borths et al. [29]. Specimen measurements from the new taxon were collected using hand calipers and ImageJ [41].

Body mass for the new taxon was estimated using equations developed for this study and by Van Valkenburgh [42] as detailed in S1 Appendix. We recognize mammalian dentition is not a perfect proxy for body mass [43], but it is possible to derive an estimate to ease communication about the new taxon using Van Valkenburgh’s [42] methods.

### Phylogenetic analysis

A phylogenetic analysis was conducted to place the Nsungwe hyaenodont in a larger systematic context within hyaenodont evolution. Of interest in this analysis are the relationships between the Nsungwe taxon and hyaenodonts from the early Oligocene and early Miocene of Afro-Arabia (e.g., [29, 44]). The Nsungwe hyaenodont is relatively incomplete, hence phylogenetic hypotheses presented here should be interpreted with caution until additional materials become available.

A character taxon matrix, modified from Borths and Seiffert [44], included 81 OTUs and 143 characters including nine characters either modified from Bastl et al. [35] or new to this study to capture the morphology of dP^3^ and dP^4^. The character matrix is S1 Dataset and a list of characters, their states, and their sources is available as S2 Appendix. The character-taxon matrix was assembled in Mesquite [45]. Eighteen multistate characters were treated as ordered, using outgroup morphology as a point of reference for character order [46]. All characters were treated as equally weighted. Additional information is included in S3 Appendix on all OTUs, including their age, formation, locality, and specimens used to score the character-taxon matrix.

For the analysis of the character-taxon matrix, we employed a model-based, Bayesian approach, a phylogenetic method applied in many recent studies of phylogenetic relationships based on morphological character information [29, 47–55]. In this case, we use “tip-dating” Bayesian inference, which incorporates the character-taxon matrix, estimated age ranges for each OTU, and a clock model to simultaneously estimate the most likely topology, divergence times, and the rate of evolution for each lineage. This allows us not only to explore topologies that are likely based on the character-taxon matrix, but also to search for topologies that are likely, given the estimated rates of evolution implied by the fossil record. This powerful analytical method is particularly useful for understanding hyaenodont evolution, as the earliest fossil evidence of this clade is a highly specialized taxon, *Lahimia* [56], from the middle Paleocene. In contrast with results obtained using Bayesian tip-dating approaches, some parsimony analyses recover *Lahimia* in a somewhat unlikely position, deeply nested within Hyaenodonta [29, 57], implying many extensive ghost lineages in the early history of the clade.

The analysis was conducted in MrBayes 3.2.6 [58] and the MrBayes formatted nexus file is available as S2 Dataset. The stratigraphic age and the sources of the estimated ages for each OTU are included in S3 Appendix. The M_k_ likelihood model was used to model morphological evolution and an independent gamma rates (IGR) relaxed clock model [58, 59] was used to estimate divergence dates and infer evolutionary rates within the tree. Hyaenodonta was constrained as an ingroup with *Tinerhodon*, *Altacreodus magnus*, and *Maelestes* designated as part of the outgroup. A prior of 100–75 Ma was used to constrain the root to be consistent with estimates of the age of Eutheria [60]. A prior of 75–62 Ma was set for the origin of Hyaenodonta, bracketing the estimates for the emergence of crown placental mammals and the earliest known hyaenodont [60, 61]. The precision of the date associated with the Nsungwe 2 locality allows us to fix the age of the Nsungwe hyaenodont at 25 Ma [27]. All other members of the in-group were assigned age ranges based on literature review. The analysis was run for 20,000,000 generations, and performed with two runs simultaneously with four Markov chains, three of which were heated (temp = 0.02), sampling every 1000^th^ generation with the first 25% discarded as part of the burn-in period. The remaining generations are incorporated into the summary statistics for the analysis. After the analysis was completed, convergence was assessed using effective sample size and average standard deviation of split frequencies statistics from the final generation. The results are summarized using an “allcompat” tree with the discussion of evolutionary rates based on median rate estimates as suggested Beck and Lee [49].

### Biogeographic analysis

Hyaenodonts are the only known carnivorous mammals occupying Afro-Arabia during the Paleogene, but they are not limited to that landmass. Hyaenodonts are also found on the northern landmasses of Asia, North America, and Europe and multiple studies have discussed close phylogenetic connections among hyaenodonts from the northern continents and Afro-Arabia [62–66]. Borths et al. [29] specifically illustrated multiple likely dispersals of hyainailourine hyaenodonts (the clade that includes *Pterodon* and *Megistotherium*) from Afro-Arabia to Europe, North America, and Asia and two possible dispersals during the Oligocene from Europe to Afro-Arabia. With the new morphological and temporal data provided by the Nsungwe taxon, we performed a biogeographic analysis with the “allcompat” consensus tree to determine how the new taxon may affect our understanding of the complicated biogeographic history of Hyaenodonta.

We used Bayesian Binary MCMC (BBM) to reconstruct the likely origins of each clade given the distribution of the taxa included in the analysis and the branch lengths implied by the phylogenetic analysis. Each OTU was assigned to one of four continental areas: Afro-Arabia, Asia, Europe, or North America. The two hyaenodont OTUs known from India–*Paratritemnodon* and *Indohyaenodon*–were placed in the “Asia” continental land area for the purposes of this analysis which is focused on exchange between Afro-Arabia and the northern continents.

The biogeographic analysis was conducted in RASP version 3.2 [67]. We hypothesize that dispersal is a more likely mechanism to explain the distribution of hyaenodonts during the Cenozoic, so the number of continental areas from which a clade could disperse was limited to one in the analysis. The MCMC analysis ran for 1,000,000 generations with 10 Markov chains sampled every 100 generations and all chains set with a temperature of 0.1. The first 100 trees were discarded as part of the burn-in period and the Jukes-Cantor model was implemented with equal among-site variation.

### Ecomorphological reconstruction

A study of Afro-Arabian carnivore diversity through the Paleogene and early Neogene was conducted to place the Nsungwe hyaenodont in a temporal and ecological context with other Afro-Arabian carnivores. In extant mammalian carnivores, the ratio of trigonid to total molar length on the lower carnassial can be used to infer diet [68, 69]. The trigonid is the portion of the tooth that bears the slicing carnassial blades. The talonid is a separate a portion of the molar that often forms a basin that occludes with the protocone. Food is ground between the protocone and talonid like a dental mortar and pestle. Carnivores that are hypercarnivores, acquiring 70% or more of their calories from meat, have a high trigonid ratio, with most of the mesiodistal length of the molar occupied by elongate carnassial blades. In contrast, generalist carnivores exhibit a lower trigonid ratio (in other words, the slicing trigonid is reduced relative to total molar length). This relatively longer grinding talonid reflects the fact that generalists acquire only 50% to 60% of their calories from meat and supplement the rest of their diet with plant matter and invertebrates [68, 70].

In this study, we calculate the trigonid ratio in all Paleogene (hyaenodonts) and early Miocene (hyaenodonts + carnivorans) carnivores. This allows us to explore the changing dietary diversity of hyaenodonts and carnivorans through the Paleogene-Neogene transition. *Prionogale* and *Namasector*, two taxa referred to the enigmatic lineage Prionogalidae from the early Miocene of Afro-Arabia, are also included in the analysis. In addition to trigonid ratio, we also collected a proxy for body mass for all carnivore taxa, using the mean mesiodistal length of M_1_ as a proxy for carnivoran body mass and mean mesiodistal length of M_2_ as a proxy for hyaenodont body mass. M_2_ is used as a proxy for body mass in hyaenodonts because it is more functionally and developmentally homologous with the carnivoran M_1_ than the often heavily worn hyaenodont M_1_ [71, 72]. Specimens measured in this study are listed in S3 Appendix. Measurements were collected using ImageJ [41].

Relevant portions of the lower dentition are not known for all hyaenodonts (e.g., *Koholia*, *Pterodon syrtos*, *Metapterodon kaiseri*). In a series of correlation studies (detailed in S1 Appendix), we demonstrate the significant correlation between measurements of the upper carnassial to the lower carnassial. The Nsungwe specimen preserves only where dP_4_ and M_1_ would have occluded. In order to provide a preliminary assessment for the Nsungwe taxon to be tested upon recovery of more complete materials, we estimate M_1_ size for RRBP 09088 based on the size of the alveoli of dP^4^ and M^1^. Regression equations used to calculate the crown measurements of dP^4^ and M^1^ are presented in S1 Appendix. We then used the regression equations calculated based on a large sample of carnassial-bearing carnivorous mammals to reconstruct dimensions of M_1_. Using M_1_ to reconstruct the body mass of the Nsungwe taxon likely underestimates the size of the animal, but is a best effort to reconstruct the body mass of the only carnivorous mammal known from the late Oligocene of Afro-Arabia. This body size estimate can help provide a backdrop for understanding the meat-eating fauna encountered by the first Afro-Arabian carnivorans that dispersed to the landmass from Eurasia.

## Results

### Systematic paleontology

### Systematic hierarchy

### Placentalia Owen, 1837 [73]

### Hyaenodonta Van Valen, 1967 [74]

### Hyainailouroidea Borths, Holroyd & Seiffert, 2016 [29]

### Genus *Pakakali* Borths & Stevens, gen. nov.

urn:lsid:zoobank.org:act:991B6709-BFFA-430C-AD11-C603C4F56E9B

#### Type species

*Pakakali rukwaensis*, sp. nov.

#### Etymology

Meaning “fierce cat” from Swahili: “paka” meaning “cat”, and “kali” meaning “fierce” or “ferocious.”

#### Generic diagnosis

As for the type and only species.

#### *Pakakali rukwaensis* Borths & Stevens, sp. nov.

urn:lsid:zoobank.org:act:A16065CB-5BC1-4441-AA12-11E79D43FDB8

Fig 2

**Fig 2:**
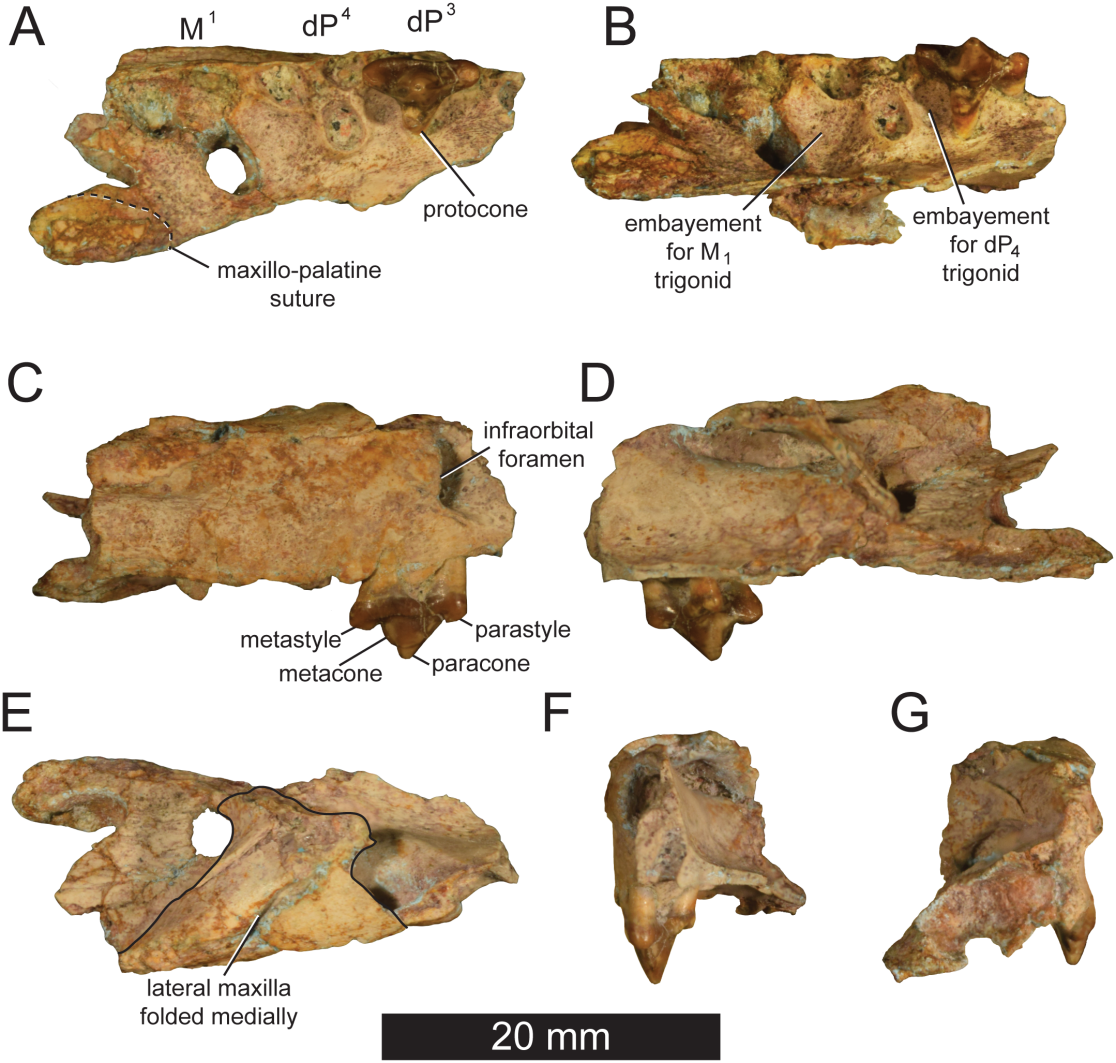
*Pakakali rukwaensis* holotype (RRBP 09088). Rostrum fragment from the right maxilla of *Pakakali rukwaensis* discovered at Nsungwe 2 locality, Nsungwe Formation, late Oligocene (~25.2 Ma) with dP^3^, alveoli of dP^4^ (or P^4^) and M^1^ in (**A**) occlusal view, (**B**) occlusal-lingual view, (**C**) buccal (lateral) view, (**D**) lingual (medial) view, (**E**) dorsal view, (**F**) mesial (rostral) view, (**G**) distal (caudal) view. Digital model of the specimen is available as S4 Appendix and as Project 303 at Morphosource.

#### Etymology

Generic epithet combines the Swahili words for cat (“paka”) and fierce (“kali”). Specific epithet refers to the Rukwa Rift Basin in which the holotype was discovered.

#### Holotype

RRBP 09088, maxilla fragment with dP^3^, alveoli for either dP^4^ or P^4^ and M^1^

#### Type locality

Late Oligocene Nsungwe Formation, locality Nsungwe 2 (~25.2 Ma), Rukwa Rift Basin, southwestern Tanzania.

#### Diagnosis

Differs from all other hyainailouroids in exhibiting the following combination of features: dP^3^ metacone closely appressed to the paracone; dP^3^ protocone mesiodistally narrow; dP^3^ paracone distally inclined; dP^3^ protocone not connected to metastyle by lingual cingulum. Further differs from *Mlanyama* and *Leakitherium* by being smaller and by having relatively thinner and smoother enamel; differs from *Teratodon* by having buccolingually compressed premolars with mesiodistally narrow protocones; differs from *Isohyaenodon pilgrimi* by being larger and by having a more lingually placed M^1^ protocone.

### Description

Specimen RRBP 09088 is a portion of the right maxilla and palatine bearing a complete dP^3^ and the alveoli of dP^4^ and M^1^. A digital model of the specimen is available as S4 Appendix and is accessioned as part of Project 303 at morphosource.org. The rostral margin of the specimen contains the infraorbital foramen dorsal to the mesial root of dP^3^, a feature commonly observed in hyaenodonts. The medial edge of the specimen is fractured along the maxillo-palatine suture just lingual to the alveolus of M^1^. A portion of the right palatine is preserved lingual to the protocone alveolus of dP^4^ and the protocone of dP^3^. The palatal process of the maxilla is embayed between the protocone of dP^3^ and the protocone alveolus of dP^4^ and between the protocone alveolus of dP^4^ and the protocone alveolus of M^1^, providing space for the occluding trigonid of dP_4_ and M_1_. The maxillary process of the specimen is fractured caudal to the infraorbital foramen. The maxillary process was folded medially postmortem and it is preserved lying superior to the infraorbital canal, obscuring the maxillary recess.

Although germs of P^3^ and P^4^ are not visible on micro-CT scans of the specimen, the tooth crown on the Nsungwe specimen is consistent in morphology with dP^3^s preserved in other hyaenodont specimens. It has three distinct roots and is mesiodistally elongate (mesiodistal length = 7.0 mm; more than 1.5 times its buccolingual width = 4.2 mm). The elongate parastyle comprises over 20% of the entire mesiodistal length of dP^3^. The parastyle is buccolingually compressed into a blade-like crista that meets the preparacrista at a defined notch. The preparacrista slopes distally at a 45-degree angle to the apex of the paracone. Like the parastyle, the paracone is buccolingually compressed. The postparacrista slopes distally at a steeper angle than the preparacrista. The postparacrista meets a distinct metacone that is fused to the distal margin of the paracone. The metacone is compressed buccolingually into a blade-like cusp. The postmetacrista slopes distally to form a deep notch at the junction with the metastyle. The metastyle, like the parastyle and paracone, is buccolingually compressed into an elongate blade. The mesiodistal length of the metastyle (2.0 mm) accounts for nearly 30% of the total mesiodistal length of the tooth. The lingual margin of the metastyle is sheer with a distal wear facet that indicates the sharp upper carnassial of dP^3^ sliced past the preprotocristid and postparacristid of the occluding dP_4_. The buccal margin of metastyle slopes to the buccal cingulum more gently than the metastylar blade slopes to the lingual margin of occlusion. A shallow basin is formed between the metastyle, buccal cingulum and the paracone. The buccal cingulum is narrow, but distinct, connecting the buccal metastyle to the buccal parastyle. Lingually, the metastyle is not connected to the protocone. Rather, the postprotocrista abuts the base of the paracone. The protocone projects lingually 1.3 mm from the base of the paracone. The distinct protocone forms the lingual-most point of a shallow talon basin that is mesially open along the preprotocrista. The preprotocrista traces the lingual-mesial face of the paracone to the lingual base of the parastyle. In occlusal view, the elongate parastyle, paracone, and metastyle are perpendicular to the lingual projection of the protocone.

The crown of dP^4^ (or possibly) P^4^ is not preserved, but the alveoli indicate the tooth had three distinct roots. The mesial root of dP^4^ is very close to the metastyle of dP^3^, indicating the metastyle of dP^3^ and parastyle of dP^4^ would have been closely packed. The lingual root of dP^4^ projects as far lingually as the lingual protocone of dP^3^. Unlike the protocone of dP^3^, the orientation of the lingual root of dP^4^ indicates the cusp was mesial relative to the paracone. The diameter of the alveolus for the lingual root of dP^4^ is equal to the diameter of the mesial root, indicating the protocone would have been a large, distinct cusp. The alveoli of M^1^ also indicate the lingual alveolus held a large, deeply anchored root for the protocone. The diameter of the lingual alveolus of M^1^ is nearly equal to the diameter of the distal alveolus. The mesial and distal alveoli of M^1^ are aligned along the buccal margin of the maxilla and the lingual alveolus is more mesially oriented than the lingual alveolus of dP^4^. The lingual alveolus of M^1^ is almost directly medial to the mesial root of M^1^.

The mesiodistal distance from the distal protocone alveolus of M^1^ to the distal protocone of dP^4^ is 7.91 mm. This measurement was used to infer the length of M_1_ with the hyaenodont carnassial regression equation presented in S1 Appendix. The estimated length of M_1_ is approximately 8.3 mm, yielding an estimated body mass of 5.8–10.1 kg with Van Valkenburgh’s [42] body mass equations as detailed in the S1 Appendix.

### Hyaenodont comparisons

Few upper deciduous teeth of hyaenodonts have been formally described, though recent efforts [35] have presented specimens that can be compared directly with *Pakakali*. As noted by Zack [75], in hyaenodonts, the morphology of a deciduous premolar closely resembles the morphology of the permanent tooth in the next position distally. Hence, dP^3^ morphology in this taxon would be expected to more closely approximate that of a permanent P^4^ than that of a permanent P^3^. As such, we compare *Pakakali* to the more restricted record of hyaenodont dP^3^s, and also to a broader sample of Afro-Arabian hyaenodont P^4^s.

Bastl, Nagel, and Peigné [35] presented one of the only comparative descriptions of the “milk tooth” or deciduous dentition of several hyaenodonts. Their study focused on species in the genus *Hyaenodon*, a genus phylogenetically distant from Afro-Arabian hyaenodonts [29, 57]. Despite the phylogenetic distance, dP^3^ in *Hyaenodon* shares several basic features with *Pakakali*. Like *Pakakali*, the infraorbital foramen of *Hyaenodon* is dorsal to the roots of dP^3^. Further, like *Pakakali*, the dP^3^ of *Hyaenodon* has a distinct metacone that is lower than the paracone and the protocone is mesiodistally narrow and projects lingually and nearly perpendicular to the buccal margin of the tooth. The amount the protocone projects lingually varies among *Hyaenodon* species, but in all dP^3^ specimens identified by Bastl and colleagues, and in *Pakakali*, the parastyle is mesiodistally elongate compared to the parastyle of P^3^ and P^4^, giving dP^3^ a “T”-like morphology in occlusal view.

**Fig 3:**
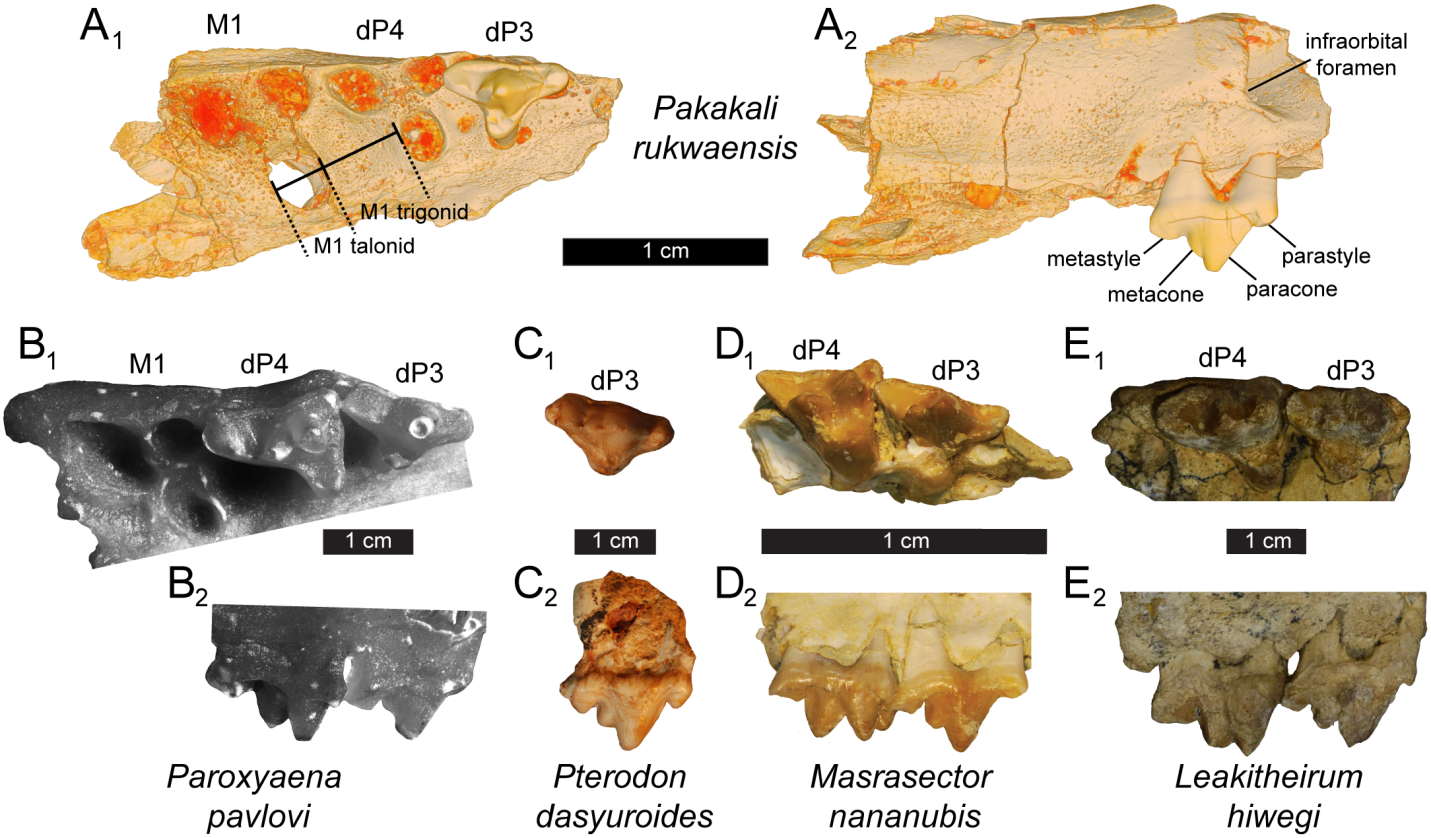
*Pakakali* dP^3^ compared with dP^3^ of other hyainailouroids. (**A**) Digital model of *Pakakali rukwaensis* right rostral fragment (RRBP 09088) with dP^3^ in (**subscript 1**) occlusal view (**subscript 2**) and buccal (lateral) view with measurements used to estimate the size of M_1_ overlaid. Comparative specimens scaled to same size as *Pakakali* with individual scale bars between the occlusal and buccal view of each specimen: (**B**) *Paroxyaena pavlovi* (images of cast of GGM no. Ca-300 and (**C**) *Pterodon dasyuroides* (BSPG 1879 XV 642), a hyainailourines from the late Eocene of Europe; (**D**) *Masrasector nananubis* (DPC 20882), a teratodontine from the late Eocene of Afro-Arabia and (**E**) *Leakitherium hiwegi* (KNM-RU 2949), a hyainailourine from the early Miocene of Afro-Arabia. Note variation in the metacone, paracone, and parastyle in each specimen.

Among hyainailourines, dP^3^ is known in *Leakitherium*, *Pterodon dasyuroides*, and *Paroxyaena* (Fig 3). These three taxa are larger than *Pakakali*, and the dP^3^ in each is more distally inclined with the preparacrista sloping steeply back toward the metastyle. Protocone morphology differs among taxa, with the protocone of *Pterodon* and *Leakitherium* mesiodistally wide, and the protocone of *Paroxyaena* and *Pakakali* mesiodistally narrow. Among these hyainailourines, the metacone of *Pakakali* is the smallest and most fully fused to the paracone. Wear along the metacone may partially explain this morphology though the base of the metacone is wider and more prominent in *Leakitherium*, *Paroxyaena*, and *Pterodon* than it is in *Pakakali*, evidence that the metacone of *Pakakali*, even when unworn, was likely closely appressed to the paracone.

Apterodontinae is the sister clade to Hyainailourinae. Like *Leakitherium* and *Pterodon*, the protocone of dP^3^ in *Apterodon* is mesiodistally wide and the talon basin shallow and not a narrow cusp like the protocone of dP^3^ of *Pakakali*. The metacone of dP^3^ in *Apterodon* is mesiodistally elongate, its base prominent, and its apex distinct from the paracone, all features that depart from the reduced, blade-like metacone of *Pakakali*.

Closer in size to *Pakakali* are several teratodontines known from dP^3^ including *Masrasector*, *Metasinopa*, *Dissopsalis* and *Anasinopa*. *Metasinopa* is closest in size to *Pakakali*. Like *Pakakali*, the parastyle and metastyle of dP^3^ in *Metasinopa* is gracile, though the protocone is relatively wider in *Metasinopa*, and the metacone is a lower, mesiodistally wider cusp than it is in *Pakakali*. *Dissopsalis*, like *Metasinopa*, has a mesiodistally elongate metacone with a prominent base. *Dissopsalis* further differs from *Pakakali* by having a relatively short metastyle that does not form a deep carnassial notch with the metacone. *Anasinopa* is close to the same size as *Pakakali* but has a mesiodistally wider protocone, a mesiodistally shorter parastyle and metastyle, and a lower paracone. Of the teratodontines known from dP^3^, *Masrasector* has the most gracile and *Pakakali*-like protocone. *Masrasector* and *Pakakali* also share a deep carnassial notch formed between the metastyle and metacone. *Masrasector* differs from *Pakakali* by having a paracone that rises significantly higher than the metacone and does not incline distally. Instead, the paracone of *Masrasector* forms a tall, acute triangle in buccal view. The paracone of *Pakakali* is lower than the paracone of *Masrasector* with a wider angle formed by the apex of the paracone.

The deciduous dentition of *Teratodon*, a taxon comparable in size to *Pakakali*, has not been described, but the morphology of *Teratodon*’s P^4^, a massive, block-like structure with difficult to discern cusps, suggests dP^3^ was not as gracile as the dP^3^ of *Pakakali*. Further, the distal alveolus of M^1^ in *Pakakali* supported a mesiodistally extensive metastyle, which contrasts with the mesiodistally short, blunt M^1^ metastyle of *Teratodon*.

The deciduous dentition of *Metapterodon kaiseri*, a hyaenodont from the early Miocene of Namibia and Kenya, is also not known, though the morphology of dP^3^ in *Pakakali* can be compared to the mid-sized hypercarnivorous *Metapterodon* P^4^. The dP^3^ of *Pakakali* has a long parastyle compared to the P^4^ of *Metapterodon* and has a buccolingually narrow metastyle. In buccal view, the paracone on dP^3^ in *Pakakali* is mesiodistally long, forming an equilateral triangle. This differs from the P^4^ paracone of *Metapterodon*, which has a narrower base and mesial and distal margins that meet at a more acute angle, like the apex formed by the dP^3^ paracone of *Masrasector*.

Two other genera of comparable size to *Pakakali* are known from the early Miocene, though neither have had deciduous upper dentition referred to them: *Mlanyama* and *Isohyaenodon*. The holotype of *Mlanyama* has thick, crenulated enamel and closely packed premolars that differ from the thin, smooth enamel of *Pakakali*. Further, the deciduous premolars of *Mlanyama* were likely more robust structures, more similar to dP^3^ of *Leakitherium* than the dP^3^ of *Pakakali*. *Isohyaenodon* is a complex genus, with at least three species: *Isohyaenodon andrewsi*, *Isohyaenodon matthewi*, and *Isohyaenodon pilgrimi*. Both *I*. *andrewsi* and *I*. *matthewi* are in similar size ranges as *Pakakali*. Based on measurements and observations, we support the hypothesis of Holroyd [40] that *I*. *andrewsi* is synonymous with *Metapterodon kaiseri*, or at least part of the same genus (though the *Metapterodon* OTU constructed for this analysis does not include material referred to *I*. *andrewsi*). *Isohyaenodon matthewi* is known from fragmentary rostral and dentary material. KNM-SO 8527 is a maxilla fragment with a portion of M^1^ preserved. The protocone of *I*. *matthewi* is reduced and the root of the protocone is close to the parastylar root. This differs from the lingually projecting M^1^ protocone alveolus of *Pakakali*. Further, the talonid of M_2_ in *I*. *matthewi* is reduced to single cusp, an unlikely morphology to occlude with the more expansive protocones of *Pakakali*. *Isohyaenodon pilgrimi* is smaller than *Pakakali* and *I*. *matthewi*, but shares with *I*. *matthewi* reduced protocones, elongate metastyles, and reduced talonids, all morphology that is not consistent with the molar morphology inferred from *Pakakali*.

### Carnivoran comparisons

Both the fossil record and molecular divergence estimates indicate carnivorans may be present in Afro-Arabia in the late Oligocene and may be preserved as part of the Nsungwe fauna [31, 32]. The oldest fossil evidence of Carnivora in Afro-Arabia is *Mioprionodon hodopeus* from the Nakwai locality in Kenya, a site that is likely early Miocene in age [76]. Molecular divergence estimates of Madagascar’s endemic carnivoran clade, Eupleridae, suggest that lineage diverged from Herpestidae about 25.5 million years ago [31]. The ancestors of euplerids have been suggested to have dispersed from Africa to Madagascar [31], indirect evidence that Carnivora may have been present in Africa at least 25.5 million years ago.

The single carnassial complex in Carnivora forms between dP^3^ and dP_4_ in immature carnivorans and P^4^ and M_1_ in mature carnivorans [8]. In all early Miocene carnivorans, including *Mioprionodon*, *Kanuites*, *Legetetia*, *Africanictis*, and *Stenoplesictis*, the mesiodistal length of P^4^ and M_1_ is greater than the mesiodistal length of M^1^ and M_2_. In each of these taxa, the trigonid of M_1_ is taller than the trigonid of M_2_ and the mesiodistal distance between the protocone of P^4^ and M_1_, the occlusal space for the trigonid of M_1_, is longer than the mesiodistal distance between the protocone of M^1^ and M^2^, the occlusal space for the trigonid of M_2_. Also in each of these carnivorans, the tall trigonid of M_1_ occludes into a deep embayment in the palate that forms distal to the short, mesially oriented protocone of P_4_. Further, the buccal margin of the maxilla distal to P^4^ is medially inflected in these carnivorans, the protocone root of M^1^ is lingually rather than mesially oriented, and the M^1^ parastyle and metastyle have similar mesiodistal lengths. In contrast to the early Miocene carnivorans of Afro-Arabia, the mesiodistal length of dP^4^ (~7.3 mm based on the alveolus to crown regressions presented in S1 Appendix) of *Pakakali* is shorter than M^1^ (7.85 mm: see S1 Appendix). Further, in *Pakakali*, it appears based on alveolar morphology that the protocones of the missing dP^4^ and M^1^ did not frame a space wider than that framed by the protocones of M^1^ and M^2^, indicating the trigonid of M_1_ in *Pakakali* was not significantly larger than the trigonid of M_2_. In Miocene Afro-Arabian carnivorans, the trigonid of M_2_ is mesiodistally shorter and lower than the trigonid of M_1_. The palate of *Pakakali* is concave between the protocone alveoli of dP^4^ and M^1^ and distal to the protocone alveolus of M^1^ (where M_2_ would have occluded), further evidence that the trigonids of M_1_ and M_2_ were both tall and prominent and both were accommodated by the palate, a morphological condition not found on the palate of early Miocene carnivorans. Distal to dP^4^, the buccal margin of the maxilla continues the lateral trend of the maxilla, the protocone alveolus of M^1^ was mesially positioned relative to the parastyle alveolus, and the M^1^ metastyle alveolus is larger than the parastyle alveolus, features consistent with a hyaenodont maxilla.

## Results and discussion

### Phylogenetic results

**Fig 4:**
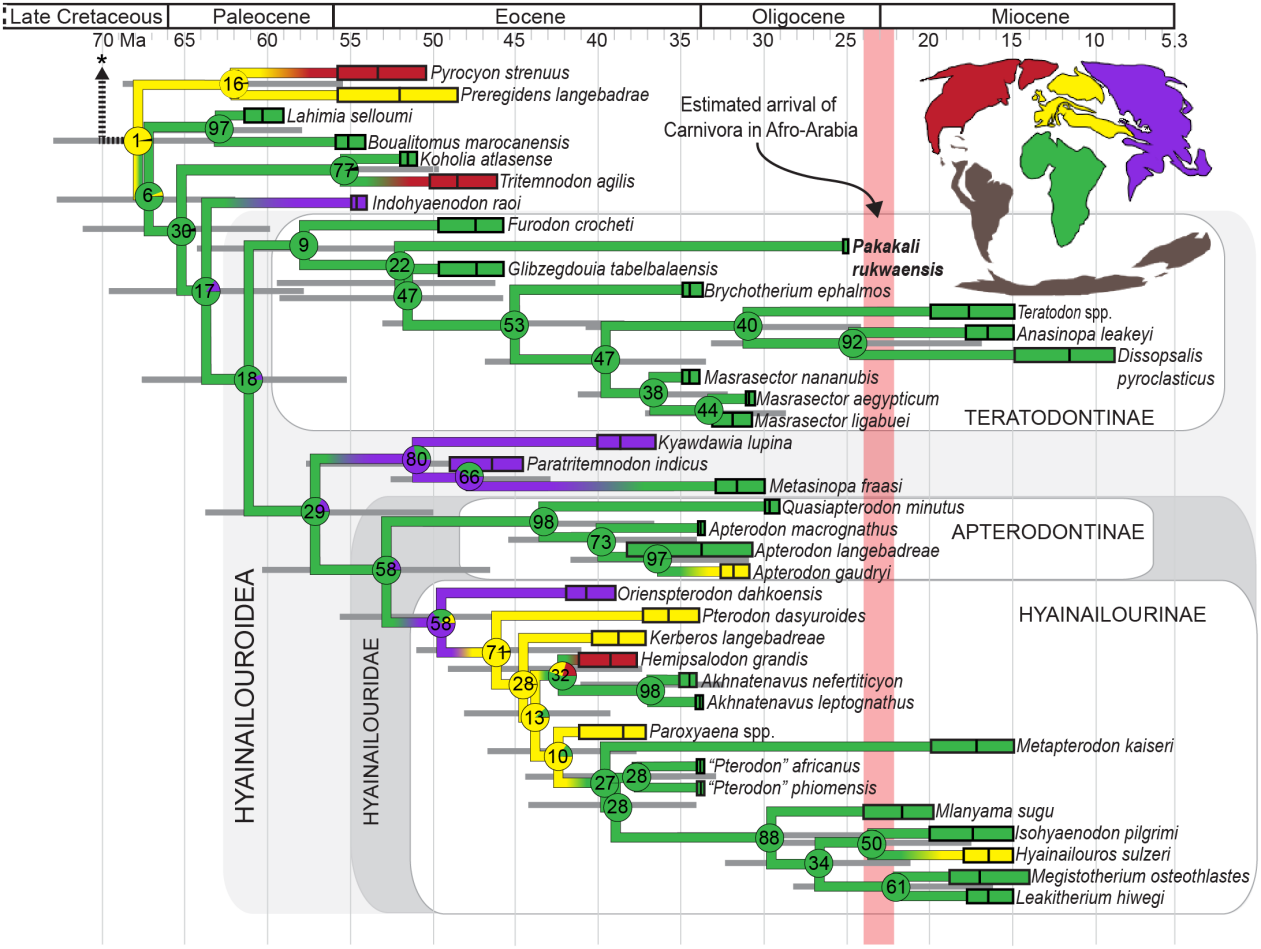
Phylogeny and biogeography of Afro-Arabian Hyaenodonta. The portion of the “allcompat” Bayesian consensus tree containing all Afro-Arabian hyaenodonts included in the Bayesian phylogenetic analysis with BBM biogeographic results summarized in circle graphs over each node. The number in the center of the circle is the posterior probability for each node. The rectangles to the left of the OTU name represent the estimated age of each OTU based on a literature review. Taxa that span a long geological interval reflect either substantial specimen sampling or imprecise dates for localities, see S3 Appendix for details of each OTU. The black vertical line in each OTU rectangle is the median estimated age of the OTU based on the Bayesian analysis. The bar for *Pakakali* is narrow because the age of the Nsungwe 2 is known with great precision. The proportion of the circle filled by each color reflects the probability that the clade originated from the corresponding continental area (**green**, Afro-Arabia; **purple**, Asia; **yellow**, Europe; **red**, North America; **grey** (on map), continents without hyaenodonts). Branches are colored with the most likely origin for each clade and gradients indicate branches that are likely dispersal events. *Pakakali* is nested within Hyainailouroidea and Teratodontinae, both clades that most likely originated in Afro-Arabia. The light red vertical bar illustrates the likely interval when Carnivora dispersed to Afro-Arabia and it crosses the hyaenodont lineages that were likely extant on the continent when carnivorans arrived. S1 Fig. and S1 Table contain information on the expanded phylogeny of Hyaenodonta.

*Pakakali rukwaensis* is recovered within Hyainailouroidea, a clade that includes all Afro-Arabian hyaenodont taxa included in the analysis except *Koholia*, *Lahimia*, and *Boualitomus*. The relationship of *Pakakali* to other Afro-Arabian hyaenodonts is shown in Fig 4, which illustrates the portion of the “allcompat” (majority rule plus compatible groups) tree relevant to the taxonomy of the species at the center of this study and statistics relevant to major Afro-Arabian clades are shown in Table 1. The “allcompat” tree with all OTUs incorporated into the analysis is S1 Fig. and statistics for all branches in the analysis are part of S1 Table. The consensus tree file output from MrBayes is S3 Dataset and the PSTAT file with overall analysis statistics is S4 Dataset. Note that the nodes and tips in these trees are illustrated at the median age of origin. All mean dates in S1 Table are offset by 11.56 Ma and all median dates are offset by 11.37 Ma. In this analysis, the overall mean clock rate is 0.0095 change/Ma and the median clock rate is 0.0059 change/Ma.

**Table 1:** Statistics related to major Afro-Arabian hyaenodont clades.

Clade	Mean Age	Median Age	HPD range	Mean Rate	Median Rate	PP Support	Most likely origin (% probability)	Second-most likely origin (% probability)
Hyaenodonta	70.4	70.1	75.99–64.55	1.77	0.92	100%	Europe (99%)	North America (<1%)
Afro-Arabian Clade	67.52	67.22	72.57–61.78	2.28	0.65	6%	Afro-Arabia (94%)	Europe (5%)
Hyainialouroidea	61.39	61.14	67.45–55.16	2.85	1.29	18%	Afro-Arabia (94%)	Asia (6%)
Teratodontinae	57.83	57.59	64.03–50.19	1.49	0.72	9%	Afro-Arabia (99%)	Asia (<1%)
*Pakakali* origin	52.39	51.95	59.26–46.04	1.17	0.64	22%	Afro-Arabia (100%)	
Hyainailouridae	53.07	52.69	60.19–46.45	2.04	1	58%%	Afro-Arabia (86%)	Asia (13%)
Apterodontinae	43.55	43.14	50.51–36.55	1.05	0.58	98%	Afro-Arabia (99%)	Asia (<1%)
Hyainailourinae	49.66	49.38	55.49–43.41	2.14	0.96	58%	Asia (65%)	Afro-Arabia (24%)
Miocene Hyainailourinae	29.73	29.48	35.47–23.76	0.86	0.54	88%%	Afro-Arabia (100%)	

All ages expressed in Ma. **HPD**, Highest probability density; **PP**, Posterior probability; Biogeographic results from Bayesian Binary MCMC analysis. For complete statistics for all clades and lineages refer to S1 Fig., S1 Table, and S3 Dataset.

Presently, the analysis is limited by the fragmentary nature of the specimen, but provides a starting point for interpreting the phylogenetic position of *Pakakali* in the context of broader hyaenodont relationships examined by Borths et al. [29]. *Pakakali* is here recovered within Hyainailouroidea, a clade defined by the node that represents the common ancestor of *Teratodon* and *Hyainailouros*. Specifically, *Pakakali* is weakly resolved as an early branching node in Teratodontinae, a clade of hyaenodonts that is Afro-Arabian in origin and predominately radiated on that continent. Teratodontinae includes taxa that are mesocarnivorous (e. g. *Masrasector nananubis*, *Glibzegdouia tabelbalaensis*) and a few taxa that are hypercarnivorous (e.g. *Dissopsalis*). The clade is estimated to have originated ~57.6 Ma. *Furodon*, an Eocene teratodontine from Algeria, is the sister taxon of all other teratodontines. After the divergence of the *Furodon* lineage, the *Pakakali* lineage diverges from the rest of Teratodontinae in the early Eocene (~52 Ma). The branch that unites *Pakakali* with all later diverging hyainailourines is weakly supported (22% posterior probability support). Three early Miocene teratodontines were part of the phylogenetic analysis: *Teratodon*, *Anasinopa*, and *Dissopsalis pyroclasticus*. *Teratodon* is weakly supported (40% posterior probability) as the sister taxon of the strongly supported clade *Anasinopa* + *Dissopsalis* (92% posterior probability).

The sister clade of Teratodontinae includes Hyainailouridae, a moderately supported clade (58% posterior probability) that includes Afro-Arabian, European, Asian, and North American taxa. Hyainailouridae likely originated between ~60.19 Ma and ~46.45 Ma. Within Hyainailouridae are the robustly supported clades Apterodontinae (98% posterior probability) and Hyainailourinae (58% posterior probability). Hyainailourinae includes six early Miocene hyainailourines: *Metapterodon*, weakly supported (27% posterior probability) as the sister taxon of a clade of Afro-Arabian OTUs that includes early Oligocene, Afro-Arabian “*Pterodon*”, and early Miocene *Isohyaenodon*, *Hyainailouros*, *Leakitherium*, *Megistotherium*, and *Mlanyama*, a taxon from the early Miocene that has not previously been incorporated into a phylogenetic analysis.

### Biogeographic results

Results of the Bayesian binary MCMC biogeographic analysis of hyaenodonts most closely related to *Pakakali* are depicted in Fig 4, together each clade’s likelihood of having originated from each of the four designated biogeographic areas (Afro-Arabia, Asia, Europe, or North America) visualized as a proportion of the circle plot over each node. Area of origin results are detailed in S1 Table. Probabilities recovered in biogeographic analyses are visualized in S1 Fig.

Based on this analysis, the clade that includes all Afro-Arabian OTUs most likely reflects a dispersal event from Europe to Afro-Arabia between the Late Cretaceous or early Paleogene. After the dispersal of the common ancestor of *Lahimia* and *Hyainailouros* to Afro-Arabia, subsequent dispersals occurred from Afro-Arabia back to the northern continents. In any case, all nodes in Teratodontinae, including the node containing *Pakakali*, are resolved as likely Afro-Arabian in origin.

The most ambiguous biogeographic dispersal in Hyainailouroidea pertains to the series of nodes near the origin of Hyainailourinae. The most likely interpretation of the biogeographic history of that group is that their common ancestor dispersed from Afro-Arabia to Asia or Europe during the early Eocene. Later, the common ancestors of *Akhnatenavus* and the common ancestors of the Miocene hyaenodonts independently dispersed from Europe to Afro-Arabia, though future investigations into the phylogenetic relationships of Hyainailourinae may refine this biogeographic scenario.

### Phylogenetic and biogeographic position of *Pakakali*

The Bayesian phylogenetic analysis resolved *Pakakali* as an early-branching lineage in the clade Teratodontinae, a clade that contains only Afro-Arabian taxa and that most likely originated in Afro-Arabia. Teratodontinae is nested within Hyainailouroidea, a clade that includes all Afro-Arabian hyaenodonts except the oldest hyaenodonts in Afro-Arabia: *Lahimia*, *Boualitomus*, and *Koholia*. Bastl et al. [35] first incorporated deciduous characters into an analysis of hyaenodont systematics with a focus on relationships within the Eurasian and North American taxon *Hyaenodon*. In this study, we expanded on the efforts of Bastl et al. [35]. Notably, even with the inclusion of these new characters, the relationships among Afro-Arabian OTUs found in this study are similar to the results of Borths et al. [29] and Borths and Seiffert [44]. Pertinent to this analysis, the placement of *Pyrocyon/Preregidens* as the sister clade to the group that contains all Afro-Arabian OTUs differs among these analyses, though the relationships of *Pyrocyon* and *Preregidens* are unstable, hence it is not unexpected that these taxa are weakly supported in this analysis. In summary, inclusion of *Pakakali* and the addition of characters describing variation in the upper deciduous dentition do not disrupt recent phylogenetic interpretations of relationships of Afro-Arabian hyaenodonts retrieved by earlier studies (e.g., [29, 44]) except by resolving *Metapterodon kaiseri* as the sister taxon of the clade that includes Fayum “*Pterodon*” and the Miocene hyainailourines, a position that differs from the results of Borths and Seiffert [44].

The only complete tooth preserved in *Pakakali* is dP^3^. As noted by Zack [75], deciduous teeth in carnivorans closely resemble and function in a way homologous to the immediately distal adult tooth. Hence, the dP^3^ of *Pakakali* likely offers insights into the morphology of P^4^. Both dP^3^ and P^4^ are part of the carnassial complex in hyaenodonts with dP^3^ shearing past dP_4_ in sub-adult hyaenodonts and P^4^ shearing past M_1_ in sub-adult and mature hyaenodonts. The dP^3^ of *Pakakali* bears a mesiodistally extensive metastyle and buccolingually compressed paracone, evidence of a well-developed carnassial shearing complex coupled with a well-developed protocone. Teratodontinae contains many Afro-Arabian taxa with extensive shearing crests and expansive protocones and trigon basins, thus the placement of *Pakakali* within this clade is consistent with the morphology preserved on the fragmentary holotype. Precise relationships resolved for *Pakakali* within Teratodontinae should be interpreted with caution until additional anatomical information of the taxon is recovered, particularly the early-branching position of the lineage within both Teratodontinae and Hyainailouroidea. Overall, there are relatively few OTUs in the analysis scored for the dP^3^ characters. Further, as noted by Bastl et al. [35] and Borths et al. [29], deciduous dental morphology can reveal phylogenetic affinities that may place a taxon known only from deciduous material in an earlier-branching phylogenetic position than might otherwise be resolved with the addition of the complete adult dentition.

Teratodontine and Hyainailourinae contain several lineages that persist across the Paleogene-Neogene boundary, an interval coincident with tectonic and climatic changes in Afro-Arabia. Both clades include lineages that diversify in the early Miocene including *Dissopsalis*, *Mlanyama*, *Metapterodon*, *Isohyaenodon*, and *Hyainailouros*. There may be several species of *Hyainailouros* [9] in the Miocene (though the diversity of the genus is contested, see [24]) and at least one, *Hyainailouros sulzeri*, dispersed from Afro-Arabia to Europe and southwestern Asia across the “Gomphothere landbridge” [21]. *Dissopsalis* and *Metapterodon* also dispersed from Afro-Arabia to southwestern Asia [77, 78] during the Miocene. These dispersal events further emphasize that the extinction of Hyaenodonta was not a simple replacement of the incumbent hyaenodonts by the newly-arrived carnivorans. Rather, Miocene hyaenodonts adapted to the changing landscape and were capable of moving into ecosystems on the northern continents previously dominated by carnivorans during the late Oligocene. Further study of *Pakakali* and its close relatives will be important for unraveling how biotic and abiotic factors affected the origination and morphological adaptations of hyainailouroids in Afro-Arabia and what biological factors may have enabled hyainailouroids to persist across three continents nearly to the end of the Miocene.

### Dental development in *Pakakali*

The holotype of *Pakakali* is not a dentally mature individual, as it retains dP^3^. Discussions of hyaenodont deciduous dentition have predominantly focused on *Hyaenodon*, a genus known from Asia, Europe, and North America [33–35, 79]. In *Hyaenodon* the terminal M^2^ is almost fully erupted into occlusion when the last deciduous tooth (dP^3^) is replaced. In Afro-Arabian hyainailouroids, dP^3^ is also replaced late in ontogeny. In many carnivoran lineages, dP^3^ is also the last deciduous tooth replaced [80], though there is variation in the eruption sequence across the clade. Hyaenodonts seem to differ from carnivorans by erupting adult dentition relatively slowly. Most carnivorans entirely replace deciduous teeth within one year [81]. Mellet [79] concluded *Hyaenodon* replaces deciduous teeth at a much slower rate than do carnivorans, allowing *Hyaenodon* to wear M_1_ and M_2_ while dP_4_ and dP_3_ were still present in the dentary. This hypothesis was tested by Bastl and Nagel [34] with a specimen of North American *Hyaenodon*; applying a forensic technique exploring cementum annulation rate, they support the hypothesis that *Hyaenodon* did not fully erupt its adult dentition until it was developmentally older, possibly between three to four years of age. Like *Hyaenodon*, the deciduous teeth of *Pakakali* likely did not erupt in a rapid sequence, a conclusion supported by a sample of hyainailouroid specimens that preserve the deciduous dentition. A specimen of the Afro-Arabian hyainailourine *Leakitherium* (KNM-RU 15182) preserves an unerupted P^4^, yet preserves no evidence of the bud of P^3^ in the maxilla above dP^3^. *Brychotherium ephalmos*, a teratodontine from the late Eocene of Egypt, is represented by a specimen (DPC 17627) that preserves the left dP^4^ along with M^1^–M^2^, and an almost fully erupted M^3^. The right side of the specimen preserves P^4^. This specimen reveals that dP^4^ was still in the process of replacement when M^3^ was almost fully erupted. Based on comparisons with other hyaenodonts, it is likely dP^3^ was present in the tooth row after all the molars erupted and P^4^ was replaced.

The dental eruption sequence and the timing of tooth replacement in *Pakakali* has important implications for the life history of the hyaenodonts that encountered the first Afro-Arabian carnivorans. The carnassial complex in hyaenodonts and carnivorans allows them to slice vertebrate tissues [70]. A functioning carnassial complex requires precise occlusion between the upper carnassial and lower carnassial. In sub-adult carnivorans dP^3^ and dP_4_ form the first carnassial complex, and is lost within the first year of a carnivoran’s life. The adult carnassial complex, formed between P^4^ and M_1_, must last the remainder of an individual’s life. Hyaenodonts also form the first carnassial complex between dP^3^ and dP_4_ and the second is formed between dP^4^ and M_1_. The late retention of dP^3^ and dP^4^ allows hyaenodonts to utilize the earliest two carnassials for a longer interval than carnivorans utilize their deciduous carnassial pair. After utilizing the carnassials formed between dP^3^/dP_4_ and dP^4^/M_1_, hyaenodonts had up to three additional adult complexes to use throughout life: P^4^/M_1_, M^1^/M_2_, and M^2^/M_3_. A dentally immature hyaenodont utilized its deciduous dentition for an extensive period of its behaviorally mature life, making deciduous dentition part of the adult phenotype with implications for the fitness of hyaenodonts on an evolutionary scale.

Carnivore ontogeny features discrete intervals of a juvenile, immature phase characterized by deciduous dentition rapidly followed by an adult, mature phase characterized by adult dentition. In contrast, it appears that hyaenodont ontogeny was a multi-step process of gradual maturation that allowed hyaenodonts to aggressively wear multiple carnassial pairs, extending the utility of the slicing teeth beyond the juvenile stage. Using the individual dental age stages (IDAS) system proposed by Anders et al. [82], hyaenodonts exhibited a long IDAS 2 phase compared to carnivorans. This may explain why taxa like the European proviverrine *Lesmesodon* and the early Miocene hyainailourine *Leakitherium* are apparently overrepresented by juvenile specimens. Prior to this study, only Bastl et al. [35] utilized deciduous dental characters in a phylogenetic analysis of hyaenodonts. Based on our observations of the hyaenodont dental record, we encourage the description of deciduous dentition to further reveal the complex life history and evolution of Hyaenodonta.

### Ecomorphology of Afro-Arabian carnivores from the Paleocene through early Miocene with special reference to *Pakakali*

**Fig 5:**
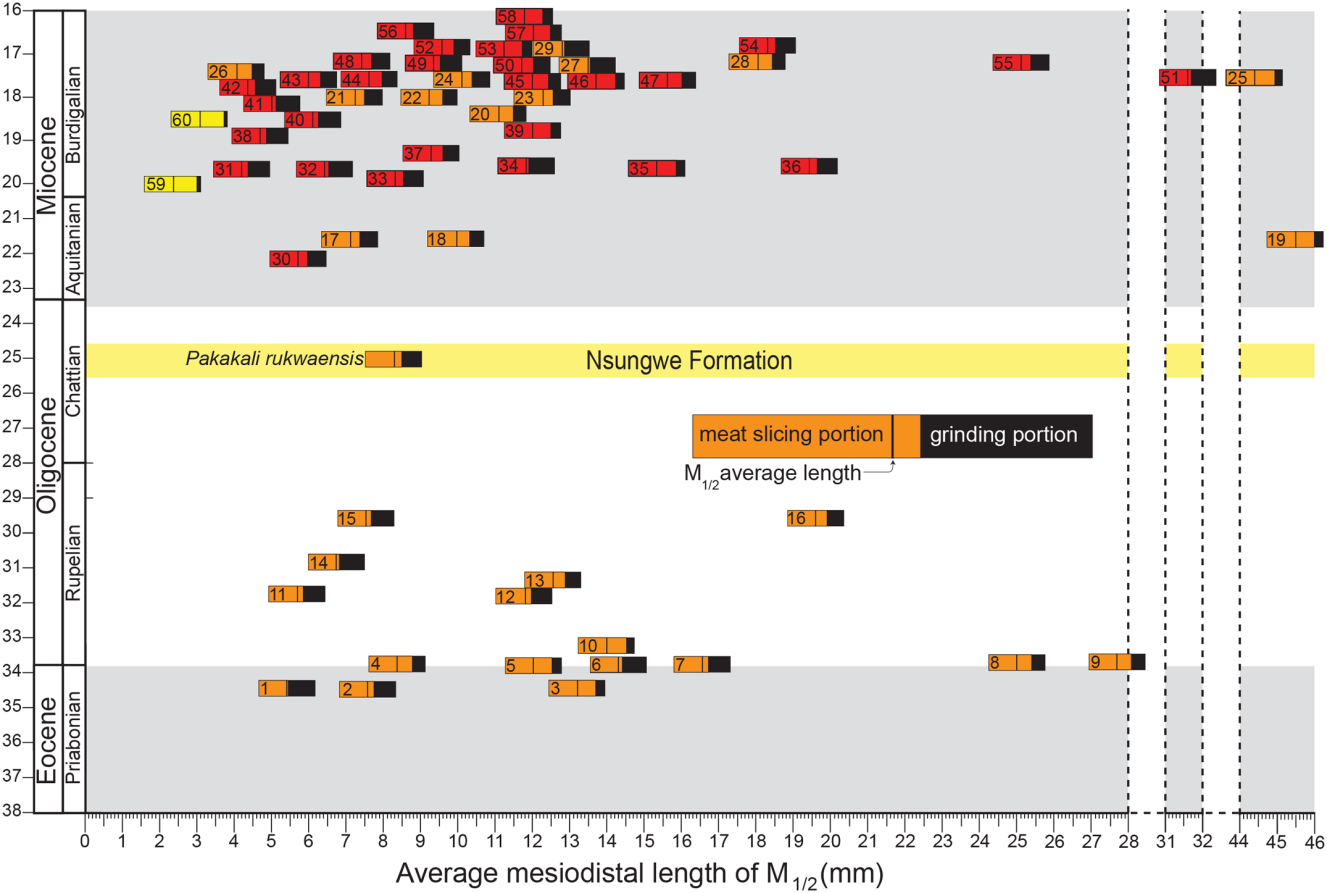
Afro-Arabian Paleogene and early Miocene carnivore morphospace occupation. A comparison the trigonid ratio for the hyaenodont M_2_ and carnivoran M_1_ in Afro-Arabia from the late Eocene through early Miocene. The X-axis is average mesiodistal length (mm) of the molars for each taxon included in the analysis as a proxy for body size. Note the scale is not continuous along the X-axis. The Y-axis is geological time expressed as geological ages and absolute age in millions of years (Ma). The timescale is not proportional in the early Miocene to accommodate the dense taxon sample through this interval. Each hyaenodont taxon included in the phylogenetic analysis is placed at the median age estimated by the tip-dating Bayesian analysis. Carnivorans found in the same localities as hyaenodonts in the analysis are placed in the same temporal range. Some taxa are found at multiple localities and the full age range estimated for each taxon is listed in Table 2 and S2 Appendix. The proportion of the horizontal bar that is colored reflects the proportion of the tooth occupied by the slicing carnassial complex. The proportion of the horizontal bar that is black reflects the proportion of the tooth occupied by the talonid complex. The longer the carnassial complex, the more vertebrate prey was likely incorporated into the diet of the taxon. **Orange**, Hyaenodonta; **Red**, Carnivora. **Yellow**, Prionogalidae. **Hyaenodonta: 1**, *Masrasector nananubis*; **2**, *Brycotherium ephalmos*; **3**, *Akhnatenavus nefertiticyon*; **4**, *“Sinopa” ethiopica*; **5**, *Metapterodon markgrafi*; **6**, *Apterodon langebadreae*; **7**, *Apterodon macrognathus*; **8**, *"Pterodon" phiomensis*; **9**, *"Pterodon" africanus*; **10**, *Akhnatenavus leptognathus*; **11**, *Masrasector ligabuei*; **12**, *Metasinopa fraasi*; **13**, *Metapterodon schlosseri*; **14**, *Masrasector aegypticum*; **15**, *Quasiapterodon minutus*; **16**, *Pterodon syrtos*; **17**, *Teratodon* (Meswa Bridge); **18**, *Mlanyama sugu*; **19**, *Hyainailouros spp*.; **20**, *Isohyaenodon matthewi*; **21**, *Teratodon* (Rusinga); **22**, *Metapterodon kaiseri*; **23**, *Isohyaenodon andrewsi*; **24**, *Buhakia moghraensis*; **25**, *Megistotherium osteothlastes*; **26**, *Isohyaenodon pilgrimi*; **27**, *Anasinopa libyca*; **28**, *Leakitherium hiwegi*; **29**, *Anasinopa leakeyi*. **Carnivora: 30**, *Mioprionodon hodopeus*; **31**, *Mioprionodon pickfordi*; **32**, *Legetetia nandii*; **33**, *Leptoplesictis namibiensis*; **34**, *Kenyalutra songhorensis*; **35**, *Ginsburgsmilus napakensis*; **36**, *Cynelos euryodon*; **37**, *Africanictis schmidtkittleri*; **38**, *Stenoplesictis muhoronii*; **39**, *Afrosmilus turkanae*; **40**, *Kichechia zamanae*; **41**, *Leptoplesictis mbitensis*; **42**, *Leptoplesictis rangwai*; **43**, *Leptoplesictis senutae*; **44**, *Herpestides aegypticus*; **45**, *Luogale rusingensis*; **46**, *Namibictis senuti*; **47**, *Diamantofelis ferox*; **48**, *Herpestides aequatorialis*; **49**, *Ketketictis solida*; **50**, *Moghradictis nedjema*; **51**, *Afrocyon burolleti*; **52**, *Africanictis meini*; **53**, *Africanictis hyaenoides*; **54**, *Amphicyon giganteus*; **55**, *Ysengrinia ginsburgi*; **56**, *Orangictis gariepensis*; **57**, *Namafelis minor*; **58**, *Syrtosmilus syrtensis*. **Prionogalidae: 59**, *Namasector soriae*; **60**, *Prionogale breviceps*.

Results of the size and degree of carnassial specialization in Paleogene and early Miocene hyaenodonts and carnivorans are shown in Fig 5 and listed in Table 2. Specimens measured for this study are listed in S2 Appendix. These calculations aim to place *Pakakali* in temporal and ecological contexts relative to other Afro-Arabian carnivores. The mesiodistal length of a lower carnassial-bearing molar is used as a proxy for body size and the ratio of the mesiodistal length of the carnassial-baring trigonid is used as a proxy for dietary specialization. Larger trigonid ratios are found in taxa that are more specialized for carnivory. Based on the estimated size of M_1_ (8.3 mm), *Pakakali* is closest in size to several early Miocene Afro-Arabian carnivorans including *Herpestes aequatorialis*, *Leptoplesictis namibiensis*, and *Herpestides aegypticus* and the early Eocene hyaenodont *Furodon crocheti* and *Glibzegdouia tabelbalaensis*, and the early Miocene hyaenodonts *Teratodon* and *Metapterodon*. The trigonid ratio of *Pakakali* is 0.65, a ratio similar to several hyaenodonts including early Eocene *Boualitomus*, late Eocene *Brychotherium*, early Oligocene *Metasinopa*, and the early Miocene *Teratodon*. The estimated trigonid ratio of *Pakakali rukwaensis* is also similar to several early Miocene carnivorans including the viverrid *Ketketictis solida*, the herpestids *Leptoplesictis rangwai*, *Leptoplesictis mbitensis*, and *Legetetia nandii*, and the amphicyonid *Amphicyon giganteus*.

**Table 2:** Tooth size and carnassialization in Afro-Arabian carnivora and Hyaenodonta.

**Carnivora**							
**Taxon**	**Code**	**m1 mesiodistal total length**	**m1 trigonid ratio**	**Age**	**Country**	**Locality**	**Family**
*Africanictis hyaenoides*	53	11.3	0.77	20–17 Ma	Kenya, Namibia	Arrisdrift, Sperrgebiet	Viverridae
*Africanictis meini*	52	9.56 (0.4)	0.71	20–17 Ma	Kenya, Namibia	Arrisdrift, Sperrgebiet	Viverridae
*Afrocyon burolleti*	51	31.6 (2.4)	0.57	19–15 Ma	Libya	Gebel Zelten	Amphicyonidae
*Amphicyon giganteus*	54	18.3	0.64	19–15 Ma	Libya, Namibia	Kipsaraman, Ngorora Member	Amphicyonidae
*Africanictis schmidtkittleri*	37	*9*.*28*	*0*.*72*	20–17 Ma	Kenya, Namibia	Chamtwara, Legetet	Viverridae
*Afrosmilus turkanae*	39	12.18	0.83	20–15.4 Ma	Kenya, Namibia	Muruotot	Barbourofelidae
*Cynelos euryodon*	36	19.5 (0.9)	0.64	20.5–16 Ma	Kenya, Uganda	Mfwangano, Songhor,	Amphicyonidae
*Diamantofelis ferox*	47	15.62	0.76	17.5–17 Ma	Namibia	Arrisdrift, Sperrgebiet	Felidae
*Ginsburgsmilus napakensis*	35	*15*.*36*	0.85	20–19 Ma	Uganda	Napak	Barbourofelidae
*Herpestides aegypticus*	44	7.61	0.73	18–10 Ma	Egypt	Wadi Moghra	Viverridae
*Herpestides aequatorialis*	48	7.43 (0.64)	0.68	18–10 Ma	Kenya, Ethiopia	Rusinga	Viverridae
*Kenyalutra songhorensis*	34	11.7	0.55	20–19 Ma	Kenya	Songhor	Mustelidae
*Ketketictis solida*	49	9.34	0.62	18–10 Ma	Egypt	Wadi Moghra	Viverridae
*Kichechia zamanae*	40	6.1 (0.4)	0.60	20.5–16.4 Ma	Kenya, Uganda	Moruorot, Rusinga	Herpestidae
*Legetetia nandii*	32	6.08 (0.36)	0.61	20–19 Ma	Kenya	Legetet, Koru, Songhor	Herpestidae
*Leptoplesictis mbitensis*	41	5.07	0.61	20.5–16.4 Ma	Kenya	Rusinga	Herpestidae
*Leptoplesictis namibiensis*	33	8.3	0.65	20.5–19 Ma	Namibia	Langental	Herpestidae
*Leptoplesictis rangwai*	42	4.36 (0.6)	0.63	20.5–16.4 Ma	Kenya	Legetet, Rusinga	Herpestidae
*Leptoplesictis senutae*	43	6	0.72	20.5–16.4 Ma	Namibia	Grillental, Sperrgebiet	Herpestidae
*Luogale rusingensis*	45	12	0.79	18–17 Ma	Kenya	Rusinga	Mustelidae
*Mioprionodon hodopeus*	30	5.89	0.74	23–22 Ma	Kenya	Nakwai	Viverridae
*Mioprionodon pickfordi*	31	5.03 (0.22)	0.72	20–19 Ma	Kenya	Songhor	Viverridae
*Moghradictis nedjema*	50	11.73	0.74	18–17 Ma	Egypt	Wadi Moghra	Viverridae
*Namafelis minor*	57	11.91	0.80	17.5–17 Ma	Namibia	Arrisdrift, Sperrgebiet	Felidae
*Namibictis senuti*	46	13.7 (1.5)	0.71	17.5–17 Ma	Namibia	Arrisdrift, Sperrgebiet	Mustelidae
*Orangictis gariepensis*	56	8.59	0.64	17.5–17 Ma	Namibia	Arrisdrift, Sperrgebiet	Viverridae
*Stenoplesictis muhoronii*	38	4.65	0.81	20–17 Ma	Kenya	Rusinga, Songhor	Viverridae
*Syrtosmilus syrtensis*	58	11.78	0.83	19–15 Ma	Libya	Gebel Zelten	Barbourofelidae
*Ysengrinia ginsburgi*	55	25.1 (2.4)	0.67	20–17 Ma	Namibia	Arrisdrift, Sperrgebiet	Amphicyonidae
**Hyaenodonta**							
**Taxon**		**m2 mesiodistal total length**	**m2 trigonid ratio**	**Age**	**Country**	**Locality**	**Family**
*Anasinopa leakeyi*	29	12.8 (1.0)	0.6	17.8–15 Ma	Kenya	Rusinga, Karugu, Mfanganu	Teratodontinae
*Anasinopa libyca*	27	13.53	0.55	19–15 Ma	Libya	Gebel Zelten	Teratodontinae
*Akhnatenavus leptognathus*	10	14	0.86	33.9–33.7 Ma	Egypt	Quarry A (Fayum)	Hyainailourinae
*Akhnatenavus nefertiticyon*	3	14.54 (0.82)	0.79	35–33.9 Ma	Egypt	Quarry L-41 (Fayum)	Hyainailourinae
*Apterodon langebadreae*	6	14.33	0.57	37–33 Ma	Libya	Dur At-Talah	Apterodontinae
*Apterodon macrognathus*	7	16.55 (1.21)	0.61	33.9–33.7 Ma	Egypt	Quarry A (Fayum)	Apterodontinae
*Boualitomus marocanensis*		3.9	0.62	55.8–54 Ma	Morocco	Grand Daoui (Ouled Abdoun Basin)	"Koholiinae"
*Brychotherium ephalmos*	2	8.41 (1.04)	0.62	35–33.9 Ma	Egypt	Quarry L-41 (Fayum)	Teratodontinae
*Buhakia moghraensis*	24	10.12	0.68	18–16.8 Ma	Egypt	Wadi Moghra	Hyainailourinae
*Dissopsalis pyroclasticus*		16.48 (0.9)	0.67	15–9 Ma	Kenya	Kaboor, Fort Ternan, Maboko, Moroto, Napak	Teratodontinae
*Furodon crocheti*		8.3	0.55	49.3–45.7 Ma	Algeria	HGL 50 (Glib Zegdou Fm)	Teratodontinae
*Glibzegdouia tabelbalaensis*		7.7	0.48	49.3–45.7 Ma	Algeria	HGL 10, HGL 50 (Glib Zegdou Fm)	Teratodontinae
*Hyainailouros spp*.	19	45.5 (0.2)	0.84	22–15 Ma	Kenya, Uganda, Namibia	Wadi Moghra, Maboko, Arrisdrift, Nakwai	Hyainailourinae
*Isohyaenodon andrewsi*	23	12.26	0.71	20–15 Ma	Kenya	Songhor, Rusinga	Hyainailourinae
*Isohyaenodon matthewi*	20	*11*.*57*	*0*.*8*	20–15 Ma	Kenya	Rusinga	Hyainailourinae
*Isohyaenodon pilgrimi*	26	4.07 (0.51)	0.8	20–15 Ma	Kenya, Uganda	Rusinga, Napak	Hyainailourinae
*Koholia atlasense*		8.4	0.65	51.8–51 Ma	Algeria	El Kohol	"Koholiinae"
*Lahimia selloumi*		5.53 (0.09)	0.64	61.6–59.2 Ma	Morocco	Ouled Abdoun Basin	"Koholiinae"
*Leakitherium hiwegi*	28	17.46	0.8	17.8–15 Ma	Kenya, Uganda	Rusinga, Napak	Hyainailourinae
*Masrasector aegypticum*	14	6.98	0.55	31–30.6 Ma	Egypt	Quarry G (Fayum)	Teratodontinae
*Masrasector ligabuei*	11	5.7	0.61	33–30.6 Ma	Oman	Taqah	Teratodontinae
*Masrasector nananubis*	1	5.44 (0.37)	0.52	35–33.9 Ma	Egypt	Quary L-41 (Egypt)	Teratodontinae
*Megistotherium osteothlastes*	25	44.38	0.87	19–14 Ma	Libya, Egypt	Gebel Zelten, Wadi Moghra	Hyainailourinae
*Metapterodon kaiseri*	22	*9*.*22*	*0*.*78*	20–15 Ma	Namibia, Kenya	Elisabethfeld, Rusinga	Hyainailourinae
*Metasinopa fraasi*	12	11.8	0.63	33–30 Ma	Egypt	Quarry A (Fayum)	Teratodontinae
*Mlanyama sugu*	18	9.94	0.74	23–21 Ma	Kenya	Nakwai	Hyainailourinae
*Metapterodon markgrafi*	5	*12*.*05*	*0*.*84*	33.9–33.7 Ma	Egypt	Quarry A (Fayum)	Hyainailourinae
*Metapterodon schlosseri*	13	12.55 (0.78)	0.72	31–31 Ma	Egypt	Quarry V (Fayum)	Hyainailourinae
*"Pterodon" africanus*	9	27.67 (0.13)	0.75	33.9–33.7 Ma	Egypt	Quarry A (Fayum)	Hyainailourinae
*Pakakali rukwaensis*		*8*.3*	*0*.*65*	24.95 Ma	Tanzania	Nsungwe 2	Hyainailourinae
*"Pterodon" phiomensis*	8	25.0 (1.41)	0.76	33.9–33.7 Ma	Egypt	Quarry A (Fayum)	Hyainailourinae
*Pterodon syrtos*	16	*19*.*59*	*0*.*7*	30–28.5 Ma	Egypt	Quarry M (Fayum)	Hyainailourinae
*Quasiapterodon minutus*	15	7.52 (0.93)	0.59	30–28.5 Ma	Egypt	Quarry M (Fayum)	Apterodontinae
*“Sinopa” ethiopica*	4	8.39	0.77	33.9–33.7 Ma	Egypt	Quarry A (Fayum)	Hyainailourinae
*Teratodon spp*.	21	7.0 (0.23)	0.64	20–15 Ma	Kenya	Songhor, Rusinga	Teratodontinae
*Teratodon spp*.	17	8.22	0.57	23–21 Ma	Kenya	Meswa Bridge	Teratodontinae
*Tinerhodon disputatus*		1.64 (0.16)	0.55	56.5–55.8 Ma	Morocco	Ouarzazate Basin	Outgroup
Prionogalidae							
**Taxon**		**m2 mesiodistal total length**	**m2 trigonid ratio**	**Age**	**Country**	**Locality**	**Family**
*Namasector soriae*	59	2.37	0.93	21–20 Ma	Namibia	Elisabethfeld	Prionogalidae
*Prionogale breviceps*	60	3.07 (0.29)	0.94	20–15 Ma	Kenya, Uganda	Songhor, Rusinga, Legetet, Chamtwara, Napak	Prionogalidae

Code used in Fig 5. Specimens measured for study listed in S2 Appendix. Values in parentheses are standard deviation. Italicized numbers indicate size of lower dentition inferred from the upper dentition using regression equations calculated in S1 Appendix.

* *Pakakali* M_1_ estimated rather than M_2_ thus size and carnassialization are likely underestimated.

The smallest carnivorans in the sample are the herpestid *Leptoplesictis rangwai* and the viverrid *Stenoplesictis muhoronii*. The largest carnivorans in the sample are the amphicyonids *Afrocyon burolleti* from Libya and *Ysengrinia ginsburgi* from Namibia. The smallest hyaenodonts in the sample are *Tinerhodon* (if the taxon is confirmed as a hyaenodont with additional fossils) and *Boualitomus* from the early Eocene and *Isohyaenodon pilgrim* from the early Miocene. The largest hyaenodonts and carnivores in the sample are *Hyainailouros* and *Megistotherium* from the early Miocene with *Pterodon africanus* from the early Oligocene occupying a slightly smaller size range than the massive early Miocene hyaenodonts.

Body mass estimations for *Pakakali* (see S1 Appendix for calculations) suggest that this hyaenodont was between 5.8 kg and 10.1 kg, comparable in size to *Lynx rufus* (bobcat) and *Cryptoprocta ferox* (fosa). Building upon dietary categories and trigonid ratios generated for extant small-bodied carnivorans described by Friscia et al. [83], the inferred trigonid ratio of *Pakakali* is similar to the trigonid ratio of many carnivorous-to-omnivorous carnivorans including *Ichneumon albicauda* (0.64; white-tailed mongoose), a sub-Saharan insectivore to omnivore; *Galidia elegans* (0.65; ring-tailed mongoose), a Malagasy carnivore; and *Urocyon cinereoargenteus* (0.61; gray fox), a North American carnivore. Body mass estimates of *Pakakali* are well below 21.5 kg, a body size range occupied by carnivorans that mostly feed on prey that is 45% or less of their body mass [84].

The Nsungwe Formation is reconstructed as seasonal, wetland ecosystem [27, 30] with aquatic and semi-aquatic vertebrates such as freshwater fish, frogs, and anthracotheres, and arboreal taxa like the primates *Nsungwepithecus* and *Rukwapithecus* [37]. The presence of the *Pakakali* holotype with little evidence of postmortem transport in this depositional context suggests the carnivore could have inhabited this complex environment much like a modern gray fox (*Urocyon cinereoargenteus*) or ringtail (*Bassariscus astutus*), pursuing small vertebrate prey across any number of substrate/habitat types.

### Paleogene-Neogene carnivore transition in Afro-Arabia

The arrival of Carnivora in Afro-Arabia is documented by fossil evidence from the earliest Miocene of Kenya [76], and potentially by the divergence of Malagasy Eupleridae from the rest of Carnivora based on molecular clock estimates [31]. The full diversity of the endemic Afro-Arabian hyaenodont fauna that preceded the arrival of Carnivora is best observed in the Fayum Depression of Egypt. The Afro-Arabian record of Hyaenodonta in the Paleocene and early to middle Eocene is improving [65, 85], but it is not until the extensive late Eocene deposits from the Fayum (Egypt) that the record reveals a complex hyaenodont fauna with a range of body sizes and dental specializations, from small, mesocarnivorous *Masrasector* to large, hypercarnivorous “*Pterodon*” *africanus* [29, 40]. The arrival of Carnivora in the late Oligocene or early Miocene is a natural ecological experiment in adaptation and niche replacement as the diverse hyaenodont fauna is injected with ecologically similar carnivorans. Examinations of carnivore competition, adaptation, or replacement (i.e. [72, 86, 87]) are feasible because, unlike omnivorous or herbivorous taxa that use soft tissue strategies in conjunction with dental adaptations to break down food, mammalian carnivory is relatively simple to extrapolate purely from teeth, which conveniently form most of the carnivorous mammal fossil record. Vertebrate muscle and bone is a material constant through the Cenozoic and multiple mammalian lineages evolved the same strategies for slicing muscle and breaking bone [70]. Using body size and the degree of carnassial specialization, both recorded in teeth, it is possible to bluntly model the niche occupied by a mammalian carnivore in modern or extinct ecosystems. The ecological niches occupied by carnivorans and hyaenodonts in Afro-Arabia can be approximated by dental size and specialization and possible interaction between carnivore communities can be explored using dental morphology. Unfortunately, the diversity of the hyaenodont fauna first encountered by carnivorans is not directly known. Before the discovery of *Pakakali* from the Nsungwe Formation, there was a substantial (~8MY) gap in the Afro-Arabian record from the late Rupelian through the Chattian (late Oligocene). The hyaenodont fauna encountered by invading Carnivora was inferred mainly from the presence of multiple hyaenodont lineages that appear to persist from the Paleogene into the Neogene. *Pakakali rukwaensis* is the first carnivorous hyaenodont described from the late Oligocene gap in Afro-Arabia. Until the recovery of a more complete record of late Oligocene hyaenodonts, this information on the relative size and inferred dental specialization of *Pakakali* affords a first step in examining adaptive trends in the Afro-Arabian carnivore community as two convergent carnivore groups encountered one another.

*Pakakali* is a relatively small carnivore (<10 kg) that likely consumed small vertebrate and invertebrate prey [80]. Hyaenodonts with similar body masses and trigonid ratios are known from the Fayum, such as *Brychotherium ephalmos* [29] and *Masrasector aegypticum* [88]. Direct comparisons are more difficult to draw between the *Pakakali* niche and early Miocene hyaenodont niches. *Teratodon* is close to *Pakakali* in body size and trigonid ratio and would seem to be an extension of the *Pakakali* niche into the Miocene if only the molars are examined. The premolars of *Teratodon* are globular and massive and their bizarre morphology reveal that this taxon was exploiting a very different niche than *Pakakali*, possibly as a durophagous carnivore [9, 89]. The other early Miocene hyaenodonts in the *Pakakali* size range, *Mlanyama* and *Metapterodon kaiseri*, are hypercarnivores with large carnassials and reduced talonid basins. Smaller *Isohyaenodon pilgrimi* is a specialized hypercarnivore like *Metapterodon*, but of the Afro-Arabian hyaenodonts it most similar in size to *Boualitomus*, a mesocarnivorous taxon from much older Eocene deposits [90]. Among early Miocene hyaenodonts, only *Anasinopa* has premolars and a trigonid ratio comparable to *Pakakali*, but at ~20 kg, *Anasinopa* is a much larger taxon.

Few hyaenodonts in the early Miocene are ecologically comparable to *Pakakali*, although many early Miocene carnivorans share broadly similar body size and carnassial specializations with *Pakakali*, including *Kichechia zamanae*, *Leptoplesictis namibiensis*, and *Herpestides aegypticus*. An abundance of small-bodied, mesocarnivorous carnivorans in African early Miocene faunas suggest that carnivorans rapidly dominated this niche soon after dispersal to the continent. Indeed, the oldest Afro-Arabian carnivoran known, *Mioprionodon hodopeus* [76], was a relatively small carnivore with a well-developed talonid basin. The precise relationships among early Miocene Afro-Arabian carnivorans and the number and timing of dispersal events to Afro-Arabia from the northern continents has not been placed within a larger phylogenetic context of carnivoran evolution, making it difficult to determine whether multiple lineages from Europe and Asia took advantage of small-bodied, mesocarnivorous niche space, or if there was a rapid endemic radiation of Afro-Arabian carnivores in the early Miocene seeded by relatively few dispersal events. Regardless, the early Miocene African carnivore fauna was diverse and populated by many small carnivorans with large talonid basins.

Hyaenodonts appear to have found other niches in the crowded early Miocene by assuming more hypercarnivorous roles. Borths et al. [29] noted a trend towards hypercarnivory among multiple lineages of Miocene hyaenodonts, but it was difficult to determine whether this trend had initiated prior to the arrival of Carnivora, or whether it occurred in response to the appearance of a new lineage of carnivorous mammals. The discovery of *Pakakali* reveals that small-bodied hyaenodonts with relatively diverse diets persisted up to the very close of the Paleogene. This suggests that the trend towards hypercarnivory may have coincided with the arrival of Carnivora, such that small-bodied mesocarnivorous carnivores helped to propel hyaenodonts toward hypercarnivory. Notably, Hyaenodonta also apparently accommodated the arrival of Carnivora by moving into novel body size niches. *Isohyaenodon* is one of the smallest meat-eaters from the early Miocene and is also among the most hypercarnivorous. At the other end of the scale, lineages that led to both *Megistotherium* and *Hyainailouros* became colossal and hypercarnivorous. Early Miocene amphicyonids like *Afrocyon burolleti* and *Ysengrinia ginsburgi* are also gigantic, yet these taxa are mesocarnivores compared to the large hyainailourines.

This model of the early Miocene carnivore community, characterized by hypercarnivorous hyaenodonts and mesocarnivorous carnivorans, is not a complete picture. Hypercarnivorous barbourofelids (e.g., *Syrtosmilus*, *Afrosmilus*, and *Ginsburgsmilus*) and felids (e.g., *Diamantofelis*, *Namafelis*) also found niche space in the early Miocene carnivore fauna of Africa. In addition, Morales et al. [23] noted that the reduction of hyaenodont diversity in the early Miocene of Namibia is coincident with the aridification of southwestern Africa. They propose that hyaenodonts were adapted to more densely forested environments than carnivorans and drier, more open localities would be expected to have more carnivorans than hyaenodonts. Future studies are needed to explore continent-wide patterns among environmental variables and carnivore faunal compositions through time.

## Conclusions

The Oligo-Miocene interval was a period of substantial climatic, tectonic, and evolutionary change throughout Afro-Arabia, with faunal evolution taking place against the backdrop of environmental changes driven by the closing of the Tethys Seaway and the fragmentation of the landscape by the East African Rift System. In the early Oligocene, Hyaenodonta was the most diverse carnivorous lineage on the landscape. By the early Miocene, hyaenodonts were restricted to hypercarnivorous niches alongside a broad range of carnivoran ecomorphs. The Nsungwe Formation in the Rukwa Rift Basin offers the only late Oligocene glimpse of terrestrial ecosystems on Africa south of the equator, from a landscape newly affected by these tectonic and climatic changes. *Pakakali*, a small-bodied, mesocarnivorous teratodontine, reveals that late Oligocene hyaenodonts were not limited to hypercarnivory or durophagy in habitats that were becoming more open and seasonally dry. But based on early Miocene finds throughout eastern Africa, the *Pakakali* niche appears not to have persisted very long for hyaenodonts. Although the trend may have begun before the arrival of Carnivora, the presence of mesocarnivorous Carnivorans likely helped to constrain niche space available to emerging hyaenodont morphotypes over time. Indeed, hyaenodonts occupied novel morphospace for the clade during the Miocene, with extreme body size variation ranging from the tiny (*Isohyaenodon*) to the enormous (*Megistotherium*), yet most assumed apex hypercarnivorous dietary niches excepting *Teratodon* and *Apterodon*. Apex carnivores in terrestrial ecosystems are particularly reliant on ecosystem stability, as the energetic requirements for these niches are substantial [36]. As such, hyaenodonts may have been more vulnerable to extinction than the invasive carnivorans, being limited from exploiting more generalized diets by their specialized hypercarnivorous dentition. The ultimate extinction of hyaenodonts in Afro-Arabia was likely not caused by direct competition with better adapted carnivorans, but rather the result of hyaenodonts adapting to specialized niches in response to the arrival of Carnivora, niches that were ultimately unstable and unsustainable on a rapidly changing landscape. Understanding how carnivores respond to invasive species and changing climatic conditions is vital for developing conservation goals for modern carnivores, and the Nsungwe Formation offers important insight into the starting point of a natural, continent-wide experiment in carnivore competition, adaptation, and extinction.

## Supporting information

S1 Appendix Body mass estimation and carnassial correlation. The body mass calculations and correlation studies performed to examine the correlation between tooth alveolus size and crown size and the correlation between upper carnassial dimensions and lower carnassial dimensions. The regression equations derived from this study were used to reconstruct the dentition of *Pakakali* and other Afro-Arabian carnivores known only from upper carnassial material.
(PDF)

S2 Appendix Character descriptions. Descriptions of characters and character states used in the phylogenetic analysis of *Pakakali*.
(DOCX)

S3 Appendix Hyaenodonta date and specimen data. Each OTU in the phylogenetic analysis with specimens used to score the character-taxon matrix, sources for the geological age ranges used in the tip-dating Bayesian analysis, and absolute age estimates.
(DOCX)

S4 Appendix *Pakakali* holotype RRBP 09088. The holotype of *Pakakali rukwaensis* embedded in a 3D PDF file. The digital model is also available for download at www.morphosource.org.
(PDF)

S1 Dataset *Pakakali* character matrix. The character-taxon matrix used in this study, formatted in the phylogenetics program Mesquite.
(NEX)

S2 Dataset *Pakakali* MrBayes input. MrBayes input file with all parameters used in the tip-dating Bayesian analysis.
(NEX)

S3 Dataset *Pakakali* allcompat consensus tree. The “allcompat” consensus tree output by MrBayes after completing the tip-dating Bayesian analysis. It is illustrated with biogeographic reconstructions in S1 Fig. and abbreviated to show the Afro-Arabian OTUs in Fig 4. The calculations necessary to adjust the timescale and % change/Ma are included in the manuscript.
(TRE)

S4 Dataset *Pakakali* PSTAT file. The PSTAT MrBayes output file for the analysis, which includes the median and mean clock rates for the analysis used to calculate the absolute % change/Ma reported in the results section, in Table 1, and S1 Table.
(PSTAT)

S1 Fig Complete consensus tree figure with biogeography. The complete phylogenetic analysis, showing the consensus with all OTUs, rather than only the Afro-Arabian OTUs shown in Fig 4 with BBM biogeographic results over each node.
(PDF)

S1 Table Complete tip-dating Bayesian statistics and biogeography results. The most important statistical results for the entire analysis. The node code corresponds to S1 Fig. The table includes support values for each clade, divergence age estimates that have been properly adjusted, and evolutionary rates for every lineage in the analysis.
(XLSX)
