# Supplementary material for: The first hyaenodont from the late Oligocene Nsungwe Formation of Tanzania: Paleoecological insights into the Paleogene-Neogene carnivore transition
Source: PLoS One. 2017 Oct 11;12(10):e0185301. doi: 10.1371/journal.pone.0185301 (PMC5636082; doi:10.1371/journal.pone.0185301)
Supplement: S1 Appendix — The body mass calculations and correlation studies performed to examine the correlation between tooth alveolus size and crown size and the correlation between upper carnassial dimensions and lower carnassial dimensions. The regression equations derived from this study were used to reconstruct the dentition of Pakakali and other Afro-Arabian carnivores known only from upper carnassial material. (PDF) [file pone.0185301.s001.pdf]

S1 Appendix: Body Mass Estimation and Carnassial Correlation

Introduction

The holotype specimen of *Pakakali rukwaensis* (RRBP 09088) is a fragment of the left rostrum that preserves the crown of dP^3 and the alveoli of dP^4 and M^1. To reconstruct the body mass and likely diet of the taxon, it is necessary to infer the dimensions of the lower molars from the alveoli of dP^4 and M^1. A series of correlation studies were conducted to test two hypotheses:

1) The dimensions of the alveoli of dP^4, P^4, and M^1 in hyaenodonts correspond to the dimensions of the crown, making it possible to assess crown dimensions based on alveolar dimensions in hyaenodonts.

2) The dimensions of the lower carnassial, particularly trigonid and talonid mesiodistal lengths, correspond to the dimensions of the upper carnassial. It is particularly important for this study to confirm that the occlusal space between the protocone of the upper carnassial and protocone of the next distal tooth corresponds with trigonid length of the occluding lower carnassial, and the mesiodistal length of the protocone corresponds with the mesiodistal length of the talonid (Fig 3 in the main text).

There are more specimens of the lower carnassials than the upper carnassials in the African carnivore fossil record. If both of these hypotheses are supported, then *Pakakali* can be placed in a larger comparative framework with other African carnivores based on inferences drawn from the alveoli of the upper carnassials, and the body mass of *Pakakali* can be estimated using the of equations developed by Van Valkenburgh (1990).

Materials and Methods

Hypothesis 1) Alveolar measurements correlate with crown measurements

To test Hypothesis 1, a sample of hyaenodont specimens was assembled that preserve the crown of P^4 or M^1 on one side of the rostrum and preserve the alveoli of P^4 or M^1 on the opposite side of the rostrum (S1 Appendix Table 1). With this sample, the dimensions of the alveoli to the dimension of the crown can be compared directly in the same individual. To assess the correspondence between alveolar dimensions and crown dimensions we collected three measurements from the alveoli and the same three measurements from the opposing crown (S1 Appendix Fig 1).

Measurement 1: Buccal length. The mesiodistal length along the buccal margin of the missing or complete crown (mesiodistal length of the mesial and distal alveoli and mesiodistal length of the buccal margin of the opposing tooth preserved with its roots and crown).

Measurement 2: Protocone length. Mesiodistal length of the protocone alveolus or root and the mesiodistal length of the protocone where the cusp occludes with the talonid.

Measurement 3. Tooth width. Buccolingual width from the buccal margin of the alveolus or crown to the lingual-most point of the protocone.

The linear measurements of the alveoli dimensions and crown dimensions were collected in ImageJ (Schneider et al., 2012). These measurements were imported into Past 3.04 (Hammer et al., 2001) and log-transformed. We used a linear, bivariate model to assess the significance of the correlation between the alveolar measurements and the crown measurements and calculate the regression equation that expresses the relationship between alveolar dimensions and crown dimensions. We hypothesize the linear dimensions of the crown can be directly inferred from the dimensions of the alveoli. Thus, along with a significant correlation between the measurements, we expect the slope of the linear best-fit model to be close to 1.

Hypothesis 2) Upper carnassial dimensions correlate with lower carnassial dimensions

To test the hypothesis that the dimensions of the lower carnassial can be directly inferred from the dimensions of the upper carnassial, we collected a sample of specimens of carnassial-bearing mammals with associated upper and lower dentitions (S1 Appendix Table 2).

Carnassial sample

Carnivora: The largest portion of this sample is composed of modern carnivorans: 66 specimens collected from 54 species. The carnivorans represent a broad sample of taxonomic and dietary diversity within Carnivora, including mesocarnivores (e. g. *Herpestes ichneumon*, *Genetta genetta*) and hypercarnivores (e. g. *Lycaon pictus*, *Panthera onca*). Carnivorans bear only one pair of carnassials on each side of the jaw and upper carnassial measurements were collected from P^4 and lower carnassial measurements were collected from M_1.

Hyaenodonta: Nine hyaenodont specimens with associated upper and lower dentition were examined as part of this study ranging in size from the weasel-sized *Thereutherium* to the bear-sized *Hemipsalodon*. Because hyaenodonts have up to three carnassial pairs on each side of the jaw, each pair was treated as a separate incidence, with the occlusal space between P^4 and M^1 correlated with the carnassial dimensions of M_1, the occlusal space between M^1 and M^2 correlated with the carnassial dimensions of M_2, and the occlusal space between M^2 and M^3 (if it is preserved) correlated with the carnassial dimensions of M_3.

Oxyaenidae: One oxyaenid specimen was examined with associated upper and lower carnassial material: *Oxyaena lupina*. Oxyaenids are united by the absence of M_3 and the presence of only two carnassial pairs on each side of the jaw. The dimensions associated with M_1 and M_2 are treated as separated incidences.

Dasyuromorphia: Dasyuromorphia is one of several metatherian lineages that converged with meat-eating eutherians in the development of carnassial dentition. The two dasyuromorphians examined here, *Sarcophilius* (Tasmanian devil) and *Thylacinus* (Tasmanian tiger), each have four carnassial pairs on each side of the jaw. As with the hyaenodonts and *Oxyaena*, each carnassial pair is treated as a separate specimen for the purposes of this study.

Measurements

To slice vertebrate prey efficiently, the carnassial complex is a structure with precise occlusion. The postparacristid and preprotocristid on the lower carnassial must shear past the upper metastylar blade and postmetacrista to allow a carnassial-bearing taxon access to animal-derived calories (Van Valkenburgh, 2007). In many hypercarnivorous taxa, the trigonid dominates the lower carnassial, and the talonid, which occludes with the upper protocone, is relatively reduced. In mesocarnivorous taxa, the talonid and protocone are mesiodistally expansive, allowing the carnassial-bearing taxon access to other food sources that require grinding, such as fruits and seeds (Friscia et al., 2007).

A series of measurements (S1 Appendix Fig 2) were collected from the sample of carnassial-bearing mammals using ImageJ (Schneider et al., 2012) to test the correlation between upper and lower occlusal portions of the carnassials.

Measurements from the **upper carnassial** (abbreviations used in figures, tables, and discussion):

UOL: *Upper occlusion length.* The total mesiodistal length from the distolingual face of the upper carnassial protocone to the distal face of the protocone of the next distal tooth (in carnivorans this is measurement from the protocone of P^4 across the protocone of M^1). This measurement reflects the points where the entire lower carnassial-bearing tooth occludes with the upper dentition. This measurement is hypothesized to have a correlation coefficient with LML near 1.

UTO: *Upper trigonid occlusion.* The mesiodistal length from the distolingual face of the upper carnassial protocone to the mesial face of the next, distal protocone. This is the space where the trigonid occludes when a carnassial-bearing mammal closes its jaws. This measurement is hypothesized to have a correlation coefficient with LTrL near 1.

UPL: *Upper protocone length.* The mesiodistal length of the protocone, from the mesial to distal margin of the cusp. This is the space where the talonid of the lower carnassial occludes with the upper dentition. Note that in hyaenodonts and dasyuromorphians this is a relatively simple measurement across the lingual portion of the tooth. In carnivorans, expanded lingual cingula and hypocones may be present on the lingual portion of the upper molars. This measurement is hypothesized to have a correlation coefficient with LTaL near 1.

Measurements from the **lower carnassial** (abbreviations used in figures, tables, and discussion):

LML: *Lower molar length*. The entire mesiodistal length of the lower carnassial-bearing tooth, from the mesial-most point of the trigonid to the distal-most point of the hypoconulid. This measurement is hypothesized to have a correlation coeffcint with UOL near 1.

LTrL: *Lower trigonid length*. The mesiodistal length of the trigonid, from its mesial-most point to the cristid obliqua and the origin of the talonid cusps. This measurement is hypothesized to have a correlation coefficient with OTO near 1.

LTaL: *Lower talonid length*. The mesiodistal length of the talonid measured from the cristid obliqua to the distal-most point of the hypoconulid. This measurement is hypothesized to have a correlation coefficient with UPL near 1.

The linear measurement of each of these dimensions was log transformed in Past 3.04 (Hammer et al., 2001) and a linear, bivariate model was used to compare each of the measurements hypothesized to be highly correlated. The hypothesis is supported if the correlation is significant and if the slope of the linear trend line has a slope near 1. If each of the correlated measurements meets these criteria, the supported hypothesis can be used to reconstruct the lower carnassial dimensions of *Pakakali* and other African carnivores known only from upper carnassial material.

Estimating the body mass of *Pakakali*

Van Valkenburgh (1990) correlated the body mass of extant carnivorans to the mesiodistal length of the carnassial-bearing M_1 and applied these correlation equations to hyaenodonts to estimate body mass in extinct specimens. If the above hypotheses are supported, we can estimate the mesiodistal length of M_1 in *Pakakali* by measuring UOL between the alveoli of dP^4 and M^1 and use the length of M_1 to estimate the body mass of *Pakakali*.

Van Valkenburgh (1990) described different sets of correlation equations for different ranges in carnivore body size. Here we use the equation for carnivorans between six and ten kilograms:

$$\text{body mass} = 10^\wedge(0.36*\log10(M_1 \text{ length})+0.43))$$

We also use the equation for carnivorans between ten and one hundred kilograms:

$$\text{body mass} = 10^\wedge(1.19*\log10(M_1 \text{ length})-0.09))$$

Among carnivorans, M_1 is the only carnassial tooth and it is actively used throughout the individual's life (Nowak, 2005; Ungar, 2010). The extinct meat eating hyaenodonts exhibited up to three carnassial-bearing lower molars as adults (Rose, 2006). Among Afro-Arabian hyaenodonts, the mesiodistal length of the lower molars increases distally. The first molar is often heavily worn over the entire occlusal surface of the tooth, indicating it would not efficiently slice vertebrate prey late into an adult lifespan. The wear facets on M_2 and M_3 of a hyaenodont more closely resemble the wear facets on M_1 of a modern carnivoran, making these teeth functionally homologous to a carnivoran's M_1 (Borths et al., 2016).

Based on the preservation of the Nsungwe specimen, we can only infer M_1 length, hence our calculations are likely to slightly underestimate the body mass of *Pakakali*, but provide general context for the potential body size niche of this late Oligocene form.

Results

Relationship between hyaenodont alveolar and crown measurements

The results of the regression analysis of alveolar dimensions on crown dimensions are shown log-transformed and in graph form in S1 Appendix Fig 3 and the regression statistics, including correlation coefficient (*r*), standard error, significance (p-value), and the regression equation are shown in S1 Appendix Table 3.

Each alveolar measurement was found to be significantly correlated with each crown measurement and the trend of each correlation was found to be close to 1. The regression equations derived from each measurement were used to calculate the likely crown dimensions of dP^4 and M^1 in *Pakakali*. The raw measurements of the alveoli are shown in S1 Appendix Table 3 and the crown measurements based on the regression equations are also presented in S1 Appendix Table 3. The buccal length of the crown of dP^4 is reconstructed as 7.3 mm long, the protocone is reconstructed as 2.3 mm long, and the buccolingual width of the tooth is 5.6 mm wide. The buccal length of the crown of M^1 is reconstructed as 8.5 mm long, the protocone is reconstructed as 2.9 mm long, and the buccolingual width of the tooth is 8.3 mm wide.

Relationship between upper carnassial and lower carnassial measurements and the body mass of *Pakakali*

The regression analyses of upper and lower carnassial measurements were performed with three different sample sets. The first sample set included all carnassials measured as part of the analysis and the results are shown in S1 Appendix Fig 4 and Table 4. The second sample set included only the hyaenodont carnassials and the results are shown in S1 Appendix Fig 5 and Table 5. The third sample set included only the carnivoran carnassials and the results are shown in S1 Appendix Fig 6 and Table 6.

For all sample sets, all upper and lower measurements were found to be highly correlated ($r > 0.9$) and significant ($p\text{-value} < 0.001$). The most highly correlated dimensions for each sample set were the relationship between upper occlusion length (UOL) and lower molar length (LML) ($r > 0.99$). The relationship between the space where the trigonid occludes (UTO) and the length of the trigonid (LTrL) is the second-most highly correlated in all sample sets ($r > 0.96$). The relationship between upper protocone length (UPL) and talonid length (LTaL) was the least tightly correlated ($r > 0.9$).

Using the regression equations derived from each sample set and each pairing of correlated measurements, the measurements from the specimen of *Pakakali* were used to calculate the dimensions of M_1 in *Pakakali*. The measurements are shown in Tables 4–6 as are the inferred dimensions of the lower carnassial for *Pakakali* (after the results of the regression equation were converted from log-transformation). Across all three sample sets, the total length of M_1 for *Pakakali* is estimated between 8.2 mm and 8.3 mm; the length of the trigonid is

between 5.35 and 5.4 mm, and the length of the talonid is between 2.7 mm and 2.9 mm. Using the Van Valkenburgh equations, *Pakakali* had an estimated body mass between 5.8 and 10.1 kg.

Discussion and Conclusion

Based on these analyses, we demonstrate there is high correlation between the size of the alveoli or dental roots of the upper dentition and the size of the crown. It is possible to use the regression equations presented here to reconstruct the size of the crown before it was worn, broken, or lost. Researchers may also simply measure the alveoli or roots and be confident that the raw measurement is likely within a few millimeters of the size of the complete crown. The largest variation in alveoli to crown dimensions examined here was the measurement of the buccal alveoli length to the crown buccal length. The metastyle in several specimens was longer than the distal root that supports that portion of the premolar or molar. In these cases, we advise the regression equations be used, or if the next distal crown is present, the metastyle may be assumed to abut the distal parastyle and the raw buccal measurement of the space used (though this method depends on the preservation of some complete crown material in the rostrum).

We also demonstrate there is a high correlation between the size of the upper carnassial and the lower carnassial in carnassial-bearing carnivores (UOL to LML). The total length of the lower carnassial (LML) is highly correlated the upper carnassial (UOL) with a low standard error, evidence this overall measurement is useful for inferring either upper or lower dimensions. The size of the trigonid (LTrL) can also be reliably reconstructed with minor error from the space in which it occludes between the protocones of the upper carnassial and the tooth distal to the carnassial (UTO).

Reconstructing the length of the talonid (LTaL) from the mesiodistal length of the protocone (UPL) includes greater error and a lower correlation coefficient than either of the other carnassial correlations. This is likely due to a number of factors, including error introduced when measuring the protocone from digital pictures. The protocone tends to be a tall, narrow structure and the cusp may be wider or narrower than expected if the protocone is slightly out of plane with the camera used to capture the image. The specimens with the highest residuals for the talonid measurement (e. g. specimen 32 = *Crocuta crocuta*; specimen 1, 2, 3 = *Acinonyx jubatus*; specimen 54 = *Panthera onca*; specimen 71 = *Hyaenodon dubius*) are all hypercarnivores with miniscule talonids that are essentially thin distal cingulids. Based on the variation in the talonid regression and the strength of the strong correlation of total carnassial length and trigonid length, we recommend the length of the talonid, when inferred from upper dentition, be calculated by the subtracting trigonid length (UTO and LTrL) from total lower carnassial length (UOL and LML).

In conclusion, despite the fragmentary preservation of RRBP 09088, the holotype of *Pakakali rukwaensis*, the strong correlation between alveolar size and crown size enables us to reconstruct the dimensions of dP^4 and M^1, two teeth only known from their alveoli in the only specimen of *Pakakali*. The strong correlation between upper carnassial dimensions and lower carnassial dimensions in carnassial-bearing mammals also allow us to reconstruct the size and proportions of M_1 in *Pakakali rukwaensis*. Measurements based on these highly significant correlations can be used to place *Pakakali* and other Afro-Arabian carnivores known only from upper dentition into the larger sample of Afro-Arabian carnivores only known from lower

carnassials. Using carnassial proportions and tooth size, we can ultimately track changes in body size and diet in the Afro-Arabian carnivore fauna through the fossil record and into the present.

References

Borths MR, Holroyd PA, Seiffert ER. Hyainailourinae and Teratodontinae cranial material from the late Eocene of Egypt and the application of parsimony and Bayesian methods to the phylogeny and biogeography of Hyaenodonta (Placentalia, Mammalia). PeerJ. 2016;4: e2639. doi: 10.7717/peerj.2639

Friscia AR, Van Valkenburgh B, Biknevicius AR. An ecomorphological analysis of extant small carnivorans. Journal of Zoology. 2007;272: 82–100. doi: 10.1111/j.1469-7998.2006.00246.x

Schneider CA, Rasband WS, Eliceiri KW. NIH Image to ImageJ: 25 years of image analysis. Nat Methods. 2012;9: 671-675. doi:10.1038/nmeth.2089

Hammer Ø, Harper DAT, Ryan PD. PAST: Paleontological statistics software package for education and data analysis. Palaeontologia Electronica. 2001;4(1): 9. Available: http://palaeo-electronica.org/2001_1/past/issue1_01.htm

Nowak RM. Walker's Carnivores of the World. Baltimore: Johns Hopkins University Press; 2005.

Rose K. The Beginning of the Age of Mammals. Baltimore: Johns Hopkins University Press; 2006.

Ungar PS. Mammal Teeth. Baltimore: Johns Hopkins University Press; 2010.

Van Valkenburgh B. Skeletal and dental predictors of body mass in carnivores. In: Damuth J, MacFadden BJ, editors. Body Size in Mammalian Paleobiology: Estimation and Biological Implications. New York: Cambridge University Press; 1990. pp. 181–206.

Van Valkenburgh B. Déjà vu: the evolution of feeding morphologies in Carnivora. Integrative and Comparative Biology. 2007;47:147–163. doi: 10.1093/icb/icm016

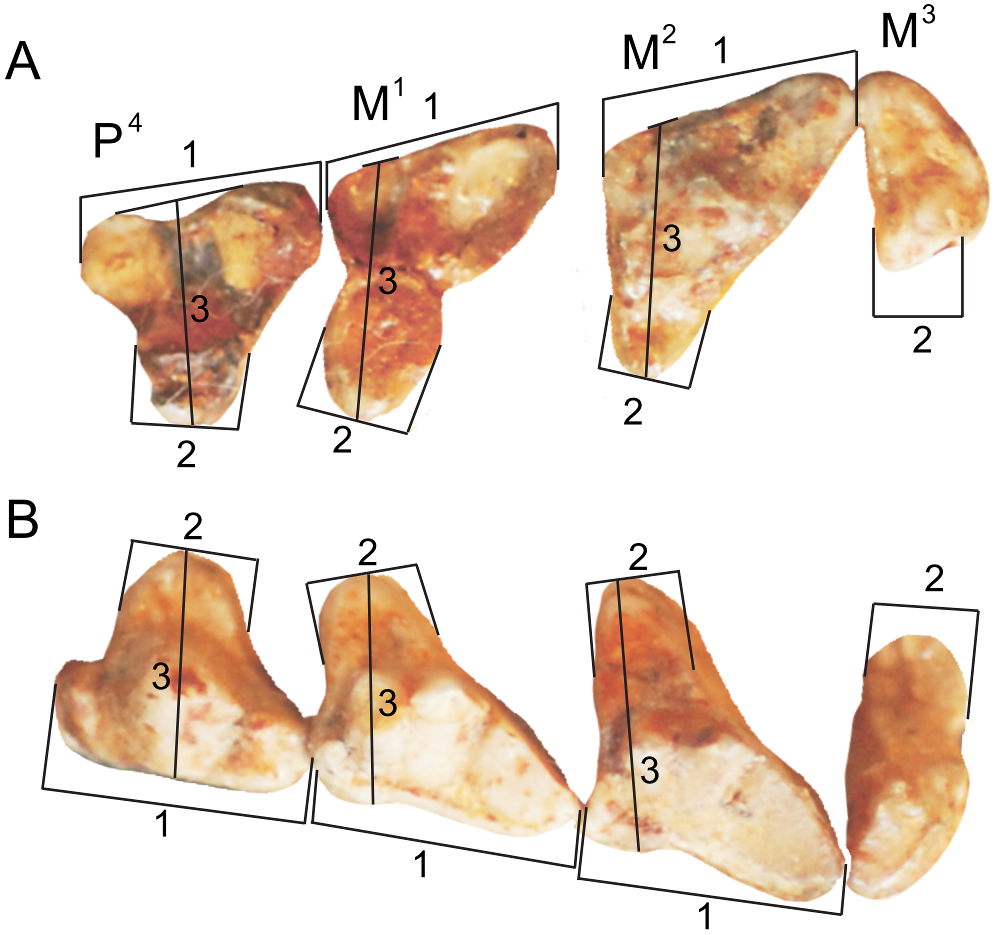

S3 Appendix Fig 1. Measurements used to compare alveoli to crown dimensions
Measurements described in detail in S3 Appendix methods section, illustrated on the upper dentition of MCZ 8901 (*Cynohyaenodon cayluxi*). **A**, Measurements on alveoli of left P4 and M1 and the heavily abraded M2; **B**, Measurements on complete right P4–M3. Raw measurements in S3 Appendix Table 1, correlation statistics between alveoli and crowns in S3 Appendix Table 3. **1**, buccal length; **2**, protocone length; **3**, tooth width.

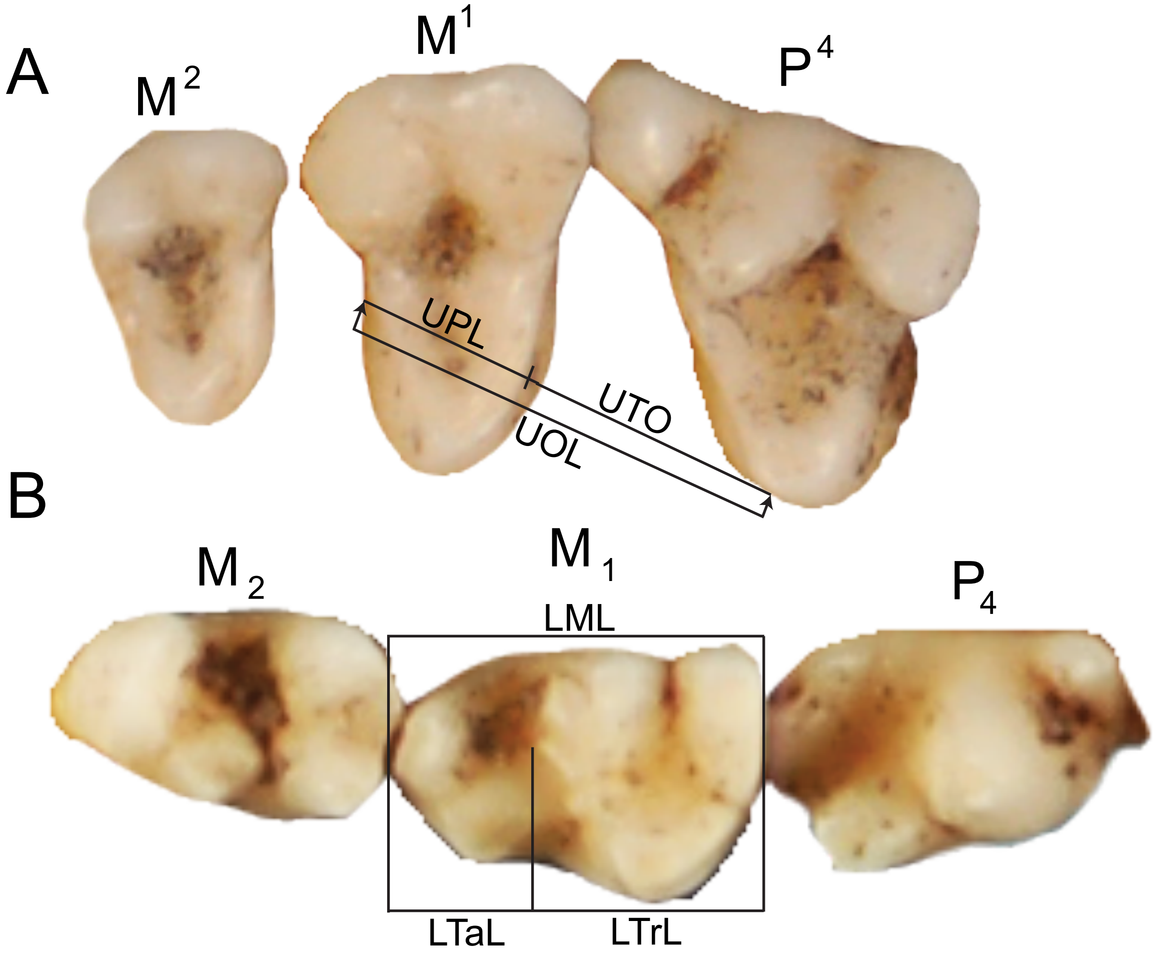

S3 Appendix Fig 2. Upper and lower carnassial measurements

Measurements shown on upper and lower right dentition of *Mungos mungo* (AMNH 83643). **A**, **UOL**, Upper occlusion length; **UTO**, Upper trigonid occlusion; **UPL**, Upper protocone length; **B**, **LML**, Lower molar length; **LTrL**, Lower trigonid length; **LTaL**, Lower talonid length.

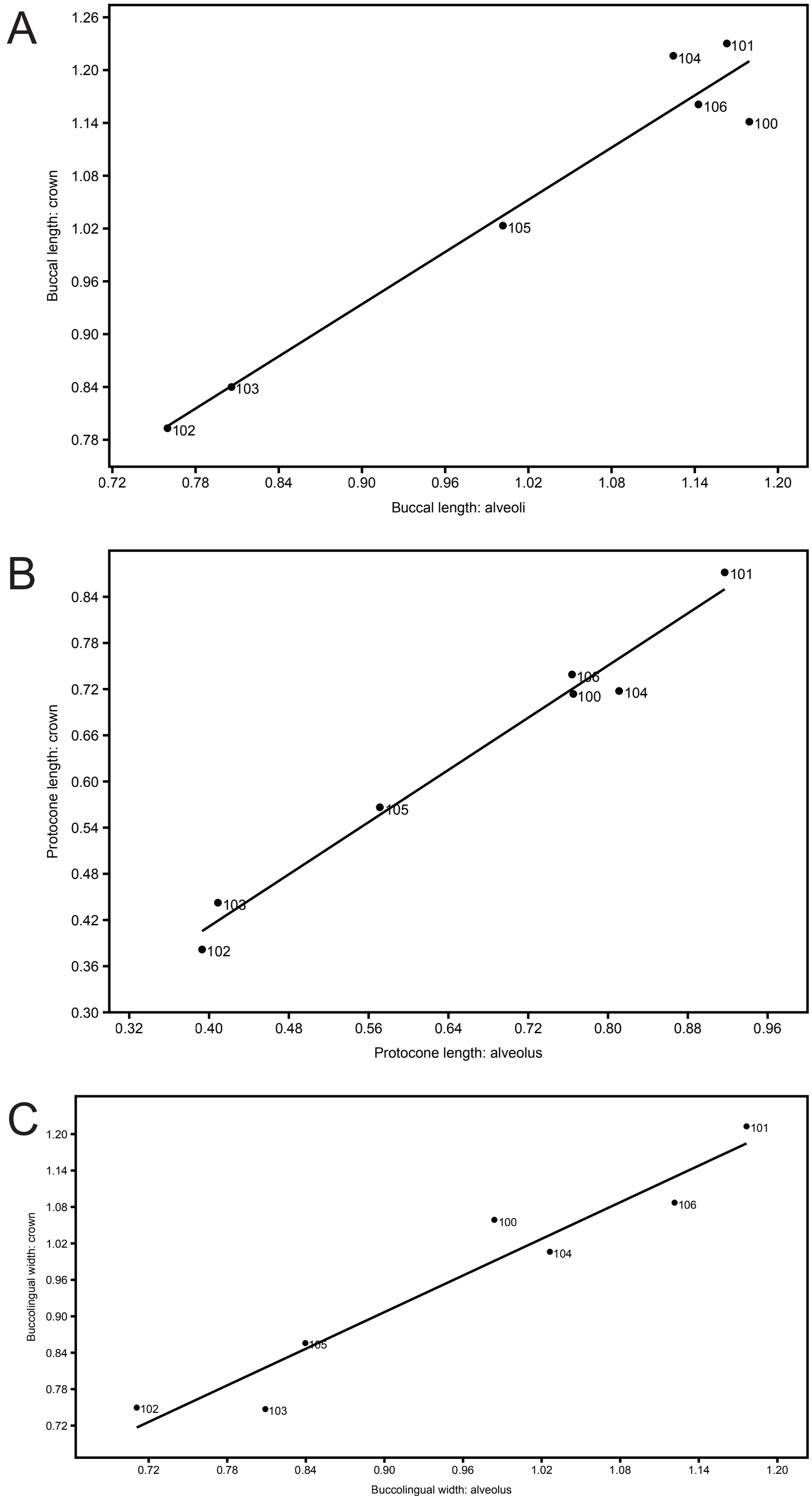

S3 Appendix Fig 3. Alevoli to crown measurement regression

Regression of alveolar dimensions to crown dimensions. Numbers refer to specimens in S3 Appendix Table 2. All measurements log transformed. Alveolar measurements on horizontal axis, crown measurements on vertical axis. Regression statistics for equations in S3 Appendix Table 3. A, buccal mesiodistal length ($r = 0.97$); B, protocone mesiodistal length ($r = 0.99$); C, buccolingual width ($r = 0.96$).

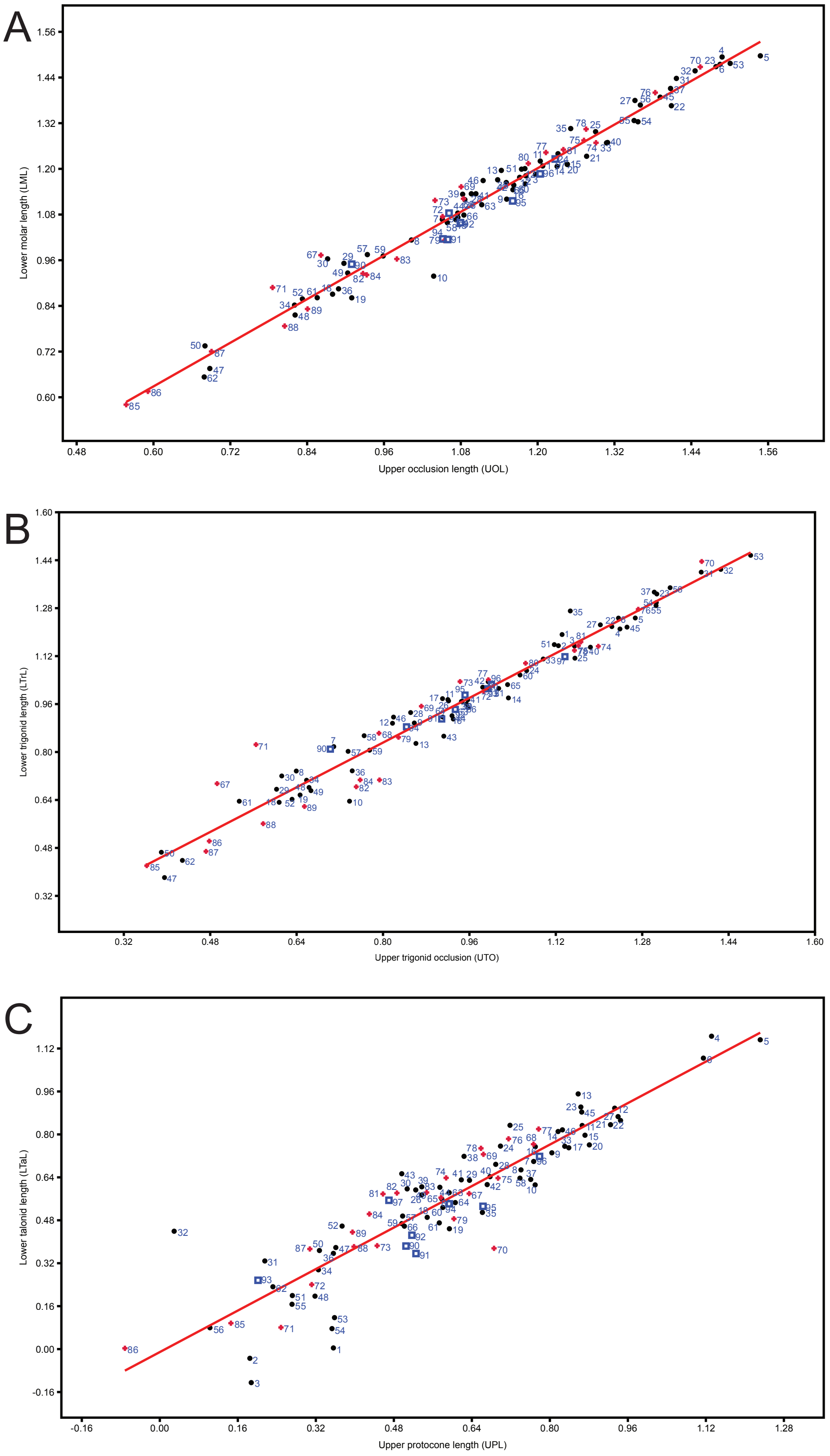

S3 Appendix Fig 4. All specimens carnassial regression

Regression of upper carnassial dimensions on lower carnassial dimensions. Numbers refer to specimens in S3 Appendix Table 2. All measurements log-transformed. Regression statistics in S3 Appendix Table 4. **Black dot**, Carnivora; **Red plus**, Hyaenodonta; **Blue square**, Dasyuromorphia. **A**, Regression of carnassial total length (UOL to LML, r = 0.99); **B**, Regression of trigonid dimensions (UTO to LTrL, r = 0.98); **C**, Regression of talonid dimensions (UPL to LTaL, r = 0.91). **UOL**, Upper occlusion length; **UTO**, Upper trigonid occlusion; **UPL**, Upper protocone length; **LML**, Lower molar length; **LTrL**, Lower trigonid length; **LTaL**, Lower talonid length.

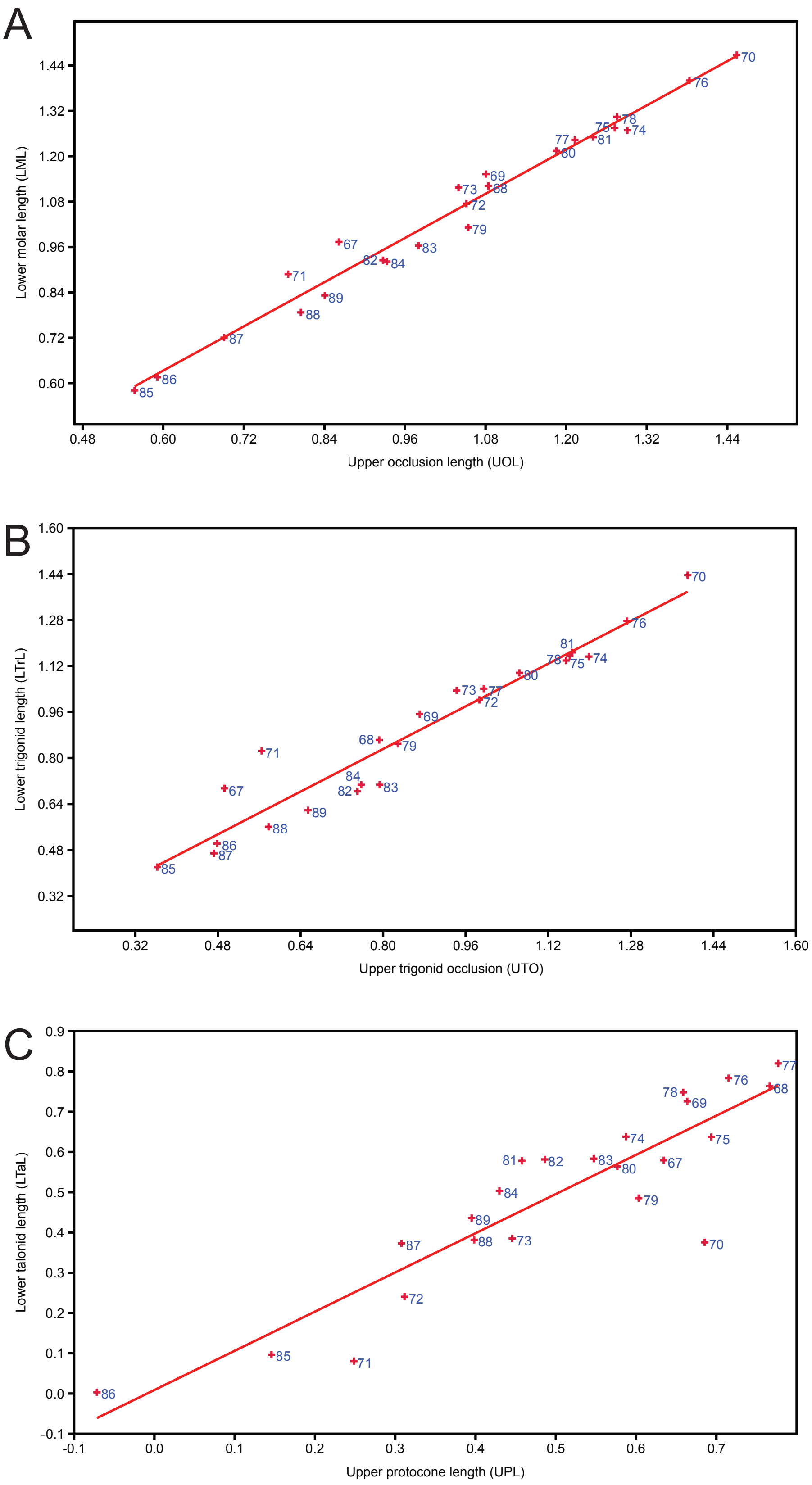

S3 Appendix Fig 5. Hyaenodonta carnassial regression

Regression of upper carnassial dimensions to lower carnassial dimensions for hyaenodont specimens only. Numbers refer to specimens in S3 Appendix Table 2. All measurements log-transformed. Regression statistics in S3 Appendix Table 5. **Red plus**, Hyaenodonta. **A**, Regression of carnassial total length (UOL to LML, r = 0.99); **B**, Regression of trigonid dimensions (UTO to LTrL, r = 0.96); **C**, Regression of talonid dimensions (UPL to LTaL, r = 0.90). **UOL**, Upper occlusion length; **UTO**, Upper trigonid occlusion; **UPL**, Upper protocone length; **LML**, Lower molar length; **LTrL**, Lower trigonid length; **LTaL**, Lower talonid length.

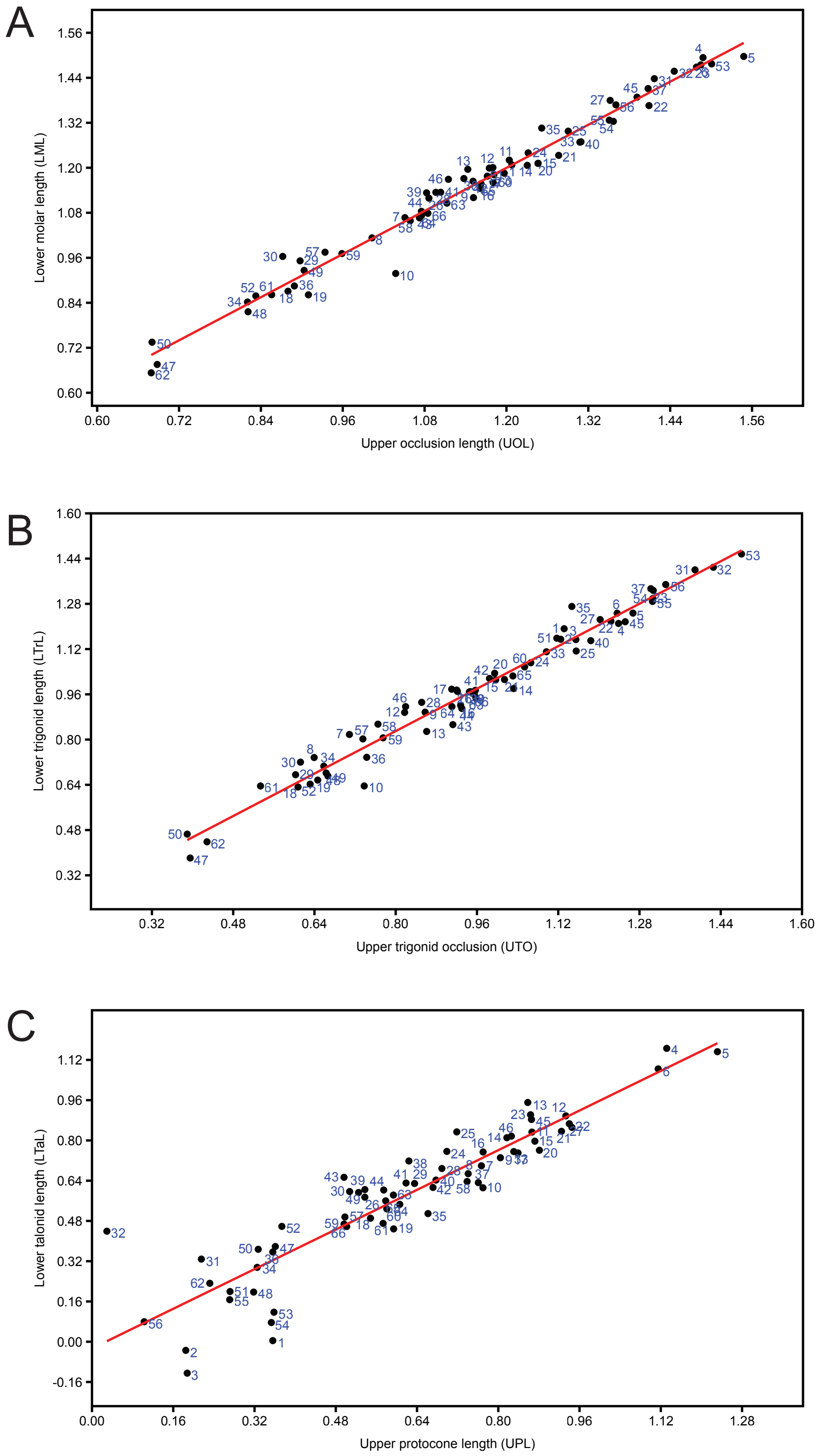

S3 Appendix Fig 6. Carnivora carnassial regression

Regression of upper carnassial dimensions to lower carnassial dimensions for carnivoran specimens only. Numbers refer to specimens in S3 Appendix Table 2. All measurements log-transformed. Regression statistics in S3 Appendix Table 6. **Black dot**, Carnivora. **A**, Regression of carnassial total length (UOL to LML, r = 0.99); **B**, Regression of trigonid dimensions (UTO to LTrL, r = 0.99); **C**, Regression of talonid dimensions (UPL to LTaL, r = 0.91). **UOL**, Upper occlusion length; **UTO**, Upper trigonid occlusion; **UPL**, Upper protocone length; **LML**, Lower molar length; **LTrL**, Lower trigonid length; **LTaL**, Lower talonid length.

S3 Appendix Table 1. Hyaenodonta alveolar mesurements to crown measurements

Specimen number	Taxon	Specimen number	Tooth locus	Alveolus side	Alveoli 1. Buccal Length	Alveoli 2. Protocone Length	Alveoli 3. Width	Whole tooth side	Whole 1. Buccal Length	Whole 2. Protocone Length	Whole 3. Width
98	*Apterodon altidens*	DPC 4486	P4	right	15.11	5.82	9.64	left	13.85	5.17	11.45
99	*Apterodon altidens*	DPC 4486	M2	right	14.56	8.26	15.01	left	16.99	7.44	16.32
100	*Cynohyaenodon cayluxi*	MCZ 8901	P4	left	5.75	2.47	5.14	right	6.21	2.41	5.62
101	*Cynohyaenodon cayluxi*	MCZ 8901	M1	left	6.4	2.56	6.44	right	6.92	2.77	5.59
102	*Hyaenodon horridus*	UCMP 22788	M1	right	13.32	6.47	10.62	left	16.45	5.22	10.14
103	*Limnocyon verus*	AMNH 12155	M1	left	10.04	3.73	6.91	right	10.55	3.69	7.18
104	*Pterodon dasyuroides*	MNHN Qu 8304	M1	right	13.89	5.81	13.23	left	14.49	5.48	12.22

Caption. Specimen number corresponds to numbers in S3 Appendix regression figures. Measurements shown in S3 Appendix Fig 1

S3 Appendix Table 2. Upper and lower carnassial measurements

Specimen number	Taxon	Specimen Number	side	UOL	UTO	UPL	LML	LTrL	LTaL
1	*Acinonyx jubatus*	AMNH 27897	right	16.14	13.54	2.27	16.13	15.56	1.01
2	*Acinonyx jubatus*	AMNH 90255	left	15.19	13.33	1.53	15.2	14.28	0.924
3	*Acinonyx jubatus*	AMNH 119657	left	15.73	14.28	1.54	15.33	14.24	0.75
4	*Ailuropoda melanleuca*	AMNH 89028	left	30.77	17.33	13.54	31.18	16.24	14.65
5	*Ailuropoda melanleuca*	AMNH 110451	right	35.308	18.5	17.04	31.4	17.668	14.209
6	*Ailuropoda melanleuca*	AMNH 110452	left	30.545	17.219	13.029	29.818	17.656	12.139
7	*Ailurus fulgens*	AMNH 35387	right	11.25	5.115	5.845	11.669	6.577	5.002
8	*Ailurus fulgens*	AMNH 119474	Right	10.067	4.359	5.505	10.307	5.452	4.654
9	*Aonyx capensis*	AMNH 51849	right	14.179	7.21	6.37	13.195	7.887	5.387
10	*Arctictis binturong*	AMNH 87388	right	10.905	5.47	5.886	8.282	4.324	4.091
11	*Arctonyx collaris*	AMNH 57118	right	16	8.32	7.35	16.61	9.44	6.81
12	*Arctonyx collaris*	AMNH 57373	right	15.14	6.57	8.57	15.87	7.87	7.9
13	*Arctonyx collaris*	AMNH 117412	Right	13.91	7.26	7.212	15.7	6.74	8.93
14	*Atelocynus microtis*	AMNH 76031	Left	17	10.77	6.56	16.087	9.56	6.468
15	*Atelocynus microtis*	AMNH 95284	right	17.63	9.928	7.448	16.28	10.26	6.265
16	*Atelocynus microtis*	AMNH 95285	right	14.502	8.468	5.89	13.97	8.339	5.676
17	*Atelocynus microtis*	AMNH 98639	left	14.856	8.132	6.9	15.052	9.509	5.626
18	*Bassariscus astutus*	AMNH 35886	right	7.581	4.052	3.535	7.426	4.287	3.097
19	*Bassariscus astutus*	AMNH 90115	right	8.122	4.43	3.926	7.264	4.536	2.808
20	*Canis adustus*	AMNH 27725	right	17.638	9.88	7.603	16.292	10.828	5.766
21	*Canis aureus*	AMNH 27735	right	18.903	10.333	8.403	17.103	10.284	6.857
22	*Canis latrans*	AMNH 141158	left	25.64	16.74	8.81	23.22	16.536	7.109
23	*Canis lupus*	AMNH 98227	left	30.101	20.28	7.304	29.414	21.246	7.979
24	*Canis mesomelas*	AMNH 27734	right	17.054	11.639	4.997	17.367	11.793	5.706
25	*Canis simensis*	AMNH 81001	left	19.523	14.301	5.227	19.844	12.974	6.821
26	*Cerdocyon thous*	AMNH 23568	right	12.198	8.344	3.445	13.154	9.344	3.753
27	*Chrysocyon brachyurus*	AMNH 71179	left	22.491	15.94	8.707	23.97	16.765	7.35
28	*Civettictis civetta*	AMNH 51803	left	12.487	7.098	4.886	13.632	8.544	4.88
29	*Conepatus fueillei*	AMNH 235512	left	7.897	4.007	4.317	8.95	4.74	4.255
30	*Conepatus mesoleucus*	AMNH 136415	left	7.447	4.097	3.216	9.195	5.25	3.953
31	*Crocuta crocuta*	AMNH 52059	right	26.107	24.508	1.642	27.393	25.13	2.13
32	*Crocuta crocuta*	AMNH 52064	right	27.922	26.641	1.07	28.671	25.665	2.749
33	*Dusicyon australis*	AMNH 235204	right	20.307	12.495	6.769	18.547	12.894	5.698
34	*Genetta genetta*	AMNH 80741	left	6.612	4.554	2.115	6.95	5.08	1.975
35	*Gulo gulo*	AMNH 37432	left	17.851	14.022	4.588	20.224	18.65	3.23
36	*Herpestes ichneumon*	AMNH 54241	left	7.746	5.533	2.27	7.67	5.46	2.273
37	*Hyaena brunnea*	AMNH 83589	left	25.563	20.06	5.764	25.776	21.553	4.287
38	*Lontra canadensis*	AMNH 182561	right	13.727	8.813	4.207	14.836	9.303	5.227
39	*Lutra longicaudis*	AMNH 205902	right	12.103	8.847	3.446	13.59	9.323	4.024
40	*Lycalopex culpaeus*	AMNH 67089	left	20.381	15.28	4.757	18.587	14.117	4.39
41	*Lycalopex griseus*	AMNH 41504	right	12.693	9.055	4.153	13.633	9.425	4.273
42	*Lycalopex gymnocercus*	AMNH 205772	right	14.159	9.652	4.693	14.59	10.378	4.102
43	*Lycalopex sechurae*	AMNH 46531	right	11.817	8.176	3.135	11.651	7.127	4.502
44	*Lycalopex vetulus*	AMNH 70091	right	11.893	8.514	3.753	12.129	8.129	4.008
45	*Lycaon pictus*	AMNH 114249	right	24.626	17.859	7.334	24.448	16.47	7.643
46	*Meles meles*	AMNH 88698	left	13.028	6.599	6.695	14.766	8.251	6.561
47	*Mungos mungo*	AMNH 83643	left	4.875	2.485	2.296	4.737	2.405	2.39
48	*Nandinia binotata*	AMNH 51469	right	6.625	4.603	2.081	6.547	4.806	1.575
49	*Nasua nasua*	AMNH 95295	left	8.005	4.64	3.349	8.44	4.689	3.918
50	*Nasuello olivacea*	AMNH 33049	left	4.791	2.452	2.125	5.431	2.921	2.33
51	*Neofelis nebulosa*	AMNH 22916	right	14.958	13.098	1.87	15.818	14.402	1.583
52	*Otocyon megalotis*	AMNH 42969	right	6.801	4.281	2.365	7.215	4.389	2.873
53	*Panthera leo*	AMNH 30244	left	31.679	30.257	2.282	29.994	28.593	1.31
54	*Panthera onca*	AMNH 36949	left	22.737	20.236	2.255	21.073	19.894	1.192
55	*Panthera pardus*	AMNH 52022	left	22.422	20.212	1.867	21.228	19.428	1.469
56	*Panthera tigris*	AMNH 35482	left	22.932	21.463	1.267	23.319	22.296	1.201
57	*Paradoxurus hermaphroditus*	AMNH 101472	left	8.591	5.437	3.148	9.436	6.342	3.131
58	*Procyon cancrivorous*	AMNH 214734	right	11.454	5.822	5.478	11.446	7.152	4.333
59	*Procyon lotor*	AMNH 150290	left	9.094	5.959	3.135	9.36	6.398	2.934
60	*Speothos venaticus*	AMNH 76035	right	15.157	11.317	3.806	14.465	11.402	3.371
61	*Spilogale putorius*	AMNH 135962	left	7.174	3.418	3.743	7.269	4.323	2.953
62	*Suricata suricatta*	AMNH 81756	right	4.778	2.682	1.706	4.5	2.744	1.707
63	*Urocyon cinereoargenteus*	AMNH 1298	left	12.964	9.072	3.922	12.757	8.875	3.827
64	*Vulpes chama*	AMNH 148759	left	11.898	8.141	4.037	11.822	8.253	3.515
65	*Vulpes fulva*	AMNH 17519	right	14.544	10.732	3.787	14.37	10.591	3.629
66	*Vulpes lagopus*	AMNH 19481	right	12.157	9.007	3.174	11.983	9.115	2.871
67	*Apterodon langebadraeae*	BMNH M85298	m1	7.27	3.11	4.31	9.403	4.95	3.797
68	*Apterodon langebadraeae*	BMNH M85298	m2	12.14	6.202	5.84	13.23	7.29	5.8
69	*Apterodon langebadraeae*	BMNH M85298	m3	12.04	7.43	4.61	14.22	8.97	5.319
70	*Hemipsalodon grandis*	AMNH	m3	28.447	24.558	4.847	29.363	27.277	2.374
71	*Hyaenodon dubius*	BMNH M2346	m1	6.109	3.671	1.772	7.731	6.681	1.204
72	*Hyaenodon dubius*	BMNH M2346	m2	11.26	9.693	2.049	11.873	10.05	1.739
73	*Hyaenodon horridus*	AMNH 75704	m1	10.958	8.763	2.791	13.099	10.842	2.428
74	*Hyaenodon horridus*	AMNH 75704	m2	19.552	15.802	3.866	18.558	14.217	4.345
75	*Kerberos langebadraeae*	MNHN	m2	18.722	14.517	4.94	18.831	14.289	4.336
76	*Kerberos langebadraeae*	MNHN	m3	24.193	18.737	5.191	25.134	18.894	6.074
77	*Oxaena lupina*	AMNH 107	m1	16.329	9.889	5.983	17.497	10.994	6.607
78	*Oxaena lupina*	AMNH 107	m2	18.867	14.274	4.557	20.155	13.773	5.6
79	*Pterodon dasyuroides*	MNHN Qu 8301	m1	11.332	6.74	4.011	10.27	7.065	3.058
80	*Pterodon dasyuroides*	MNHN Qu 8301	m2	15.326	11.588	3.773	16.372	12.481	3.667
81	*Pterodon dasyuroides*	MNHN Qu 8301	m3	17.386	14.67	2.869	17.804	14.702	3.785
82	*Sinopa rapax*	AMNH 13142	m1	8.457	5.629	3.064	8.414	4.831	3.815
83	*Sinopa rapax*	AMNH 13142	m2	9.553	6.216	3.526	9.19	5.092	3.832
84	*Sinopa rapax*	AMNH 13142	m3	8.571	5.724	2.69	8.347	5.092	3.187
85	*Thereutherium thylacodes*	MNHN Qu 8580	m1	3.609	2.304	1.399	3.807	2.635	1.249
86	*Thereutherium thylacodes*	MNHN Qu 8580	m2	3.902	3.008	0.848	4.126	3.182	1.007
87	*Masrasector nananubis*	DPC 13285	m1	4.905	2.966	2.031	5.251	2.941	2.361
88	*Masrasector nananubis*	DPC 13285	m2	6.381	3.785	2.502	6.124	3.638	2.408
89	*Masrasector nananubis*	DPC 13285	m3	6.928	4.513	2.484	6.791	4.153	2.728
90	*Sarcophilius harrisii*	AMNH 65673	m1	8.121	5.041	3.198	8.905	6.451	2.421
91	*Sarcophilius harrisii*	AMNH 65673	m2	11.47	8.104	3.353	10.338	8.132	2.27
92	*Sarcophilius harrisii*	AMNH 65673	m3	12.004	8.587	3.289	11.443	8.754	2.657
93	*Sarcophilius harrisii*	AMNH 65673	m4	11.525	9.868	1.59	12.124	10.26	1.805
94	*Thylacinus cynocephalus*	AMNH 35866	m1	11.273	6.974	3.926	10.374	7.659	3.478
95	*Thylacinus cynocephalus*	AMNH 35866	m2	14.488	8.951	4.601	13.055	9.773	3.405
96	*Thylacinus cynocephalus*	AMNH 35866	m3	15.996	10.018	6.01	15.377	10.606	5.223
97	*Thylacinus cynocephalus*	AMNH 35866	m4	16.887	13.705	2.951	16.823	13.129	3.585

Specimen numbers correspond to numbers in S3 Appendix regression figures. **UOL**, Upper occlusion length; **UTO**, Upper trigonid occlusion, **UPL**, Upper protocone length; **LML**, Lower molar length; **LTrL**, Lower trigonid length; **LTaL**, Lower talonid length. 1–66, Carnivora; 67–89, Hyaenodonta; 90–97, Dasyuromorphia. All measurements in mm.

S3 Appendix Table 3. Alveoli to crown regression statistics

Measurement	Regression slope	Std Error of slope	*r*	*p*	Regression equation	*Pakakali* dP4 alveoli	*Pakakali* M1 alveoli	*Pakakali* dP4 crown	*Pakakali* M1 crown
1. Buccal length	0.99	0.104	0.97	<0.001	y=0.044+0.99x	6.73	7.83	7.30	8.49
2. Protocone length	0.85	0.056	0.99	<0.001	y=0.072+0.85x	2.23	2.90	2.33	2.92
3. Tooth width	1.00	0.125	0.96	<0.001	y=0.003+1.00x	5.55	8.20	5.59	8.25

Measurements defined in Hypothesis 1 methods. *r*, correlation coefficient between alveolus and crown measurements. *p*, p-value. *Pakakali* alveolar measurements from specimen. *Pakakali* crown measurements based on alveoli from from regression equations

S3 Appendix Table 4. Correlation of upper and lower carnassials for all specimens

All Specimens	Regression slope	Std Error of slope	*r*	*p*	Regression equation	*Pakakali* Upper	*Pakakali* Lower
UOL to LML	0.96	0.016	0.99	<0.001	y=0.055+0.96x	7.91	8.26
UTO to LTrL	0.93	0.2	0.98	<0.001	y=0.086+0.93x	4.9	5.35
UPL to LTaL	0.97	0.45	0.91	<0.001	y=-0.011+0.97x	2.9	2.78

S3 Appendix Table 5. Correlation of upper and lower carnassials for only hyaenodont specimens

Hyaenodonta	Regression slope	Std Error of slope	*r*	*p*	Regression equation	*Pakakali* Upper	*Pakakali* Lower
UOL to LML	0.97	0.034	0.99	<0.001	y=0.048+0.97x	7.91	8.3
UTO to LTrL	0.93	0.058	0.96	<0.001	y=0.09+0.93x	4.9	5.4
UPL to LTaL	0.97	0.103	0.9	<0.001	y=0.009+0.97x	2.9	2.91

S3 Appendix Table 6. Correlation of upper and lower carnassials for only Carnivora specimens

Carnivora	Regression slope	Std Error of slope	*r*	*p*	Regression equation	*Pakakali* Upper	*Pakakali* Lower
UOL to LML	0.96	0.019	0.99	<0.001	y=0.05+0.96x	7.91	8.17
UTO to LTrL	0.94	0.02	0.99	<0.001	y=0.079+0.94x	4.9	5.35
UPL to LTaL	0.98	0.055	0.91	<0.001	y=-0.027+0.98x	2.9	2.71

Std Error, standard error. ***r***, correlation coefficient. ***p***, p-value. ***Pakakali* Upper**, measurements from specimen. ***Pakakali* Lower**, dimensions of lower carnassial based on regression equation. **UOL**, Upper occlusion length; **UTO**, Upper trigonid occlusion; **UPL**, Upper protocone length; **LML**, Lower molar length; **LTrL**, Lower trigonid length; **LTaL**, Lower talonid length. All measurements in mm.
