# Supplementary material for: The first hyaenodont from the late Oligocene Nsungwe Formation of Tanzania: Paleoecological insights into the Paleogene-Neogene carnivore transition
Source: PLoS One. 2017 Oct 11;12(10):e0185301. doi: 10.1371/journal.pone.0185301 (PMC5636082; doi:10.1371/journal.pone.0185301)
Supplement: S3 Appendix — Each OTU in the phylogenetic analysis with specimens used to score the character-taxon matrix, sources for the geological age ranges used in the tip-dating Bayesian analysis, and absolute age estimates. (DOCX) [file pone.0185301.s003.docx]

**Hyaenodonta**

***Akhnatenavus leptognathus***

Formation: Jebel Qatrani Formation

Locality: Quarry A, lower Sequence

Geological Age: Rupelian, Oligocene

Absolute Age: 33.9–33.7 Ma

Country: Fayum, Egypt

Citation: Seiffert, 2010; Holroyd, 1999

Specimens observed: AMNH 13263 (Holotype), dentary with P_3_–M_3_

***Akhnatenavus nefertiticyon***

Formation: Jebel Qatrani Formation

Locality: L-41

Geological Age: late Priabonian, latest Eocene

Absolute Age: 35–33.9 Ma

Country: Egypt

Citation: Holroyd, 1994; Seiffert, 2010; this study

Specimens observed: CGM 83735 (Holotype), cranium with C, P^2^–M^3^; DPC 13518, maxilla with M^1^; DPC 18242, cranium with P^2^–M^2^; DPC 7765, dentary with P_2_–M_3_; DPC 15250, dentary with P_2_, P_3_, M_2_

***Allopterodon torvidus***

Formation: Fissure fill, between Bouxwiler and Lissieu levels zone

Locality: Egerkingen

Geological Age: MP 14, Lutetian

Absolute Age: 42.6–42.8

Country: Switzerland

Citation: Hartenberger, 1970; Lange-Badré, 1984; Polly and Lange-Badré, 1993; Becker, Rauber, & Scherler, 2013

Specimens observed: NMB En 522 (Holotype), rostrum with P^3^–M^3^; NMB En 172, dentary with M_2_–M_3_ (erupting); UCMP 140644 (cast of Egerkingen specimen), dentary with P_1_–M_3_; NMB En. 167, maxilla with P^4^–M^3^; NMB Em. 16, partial cranium with P^4^–M^3^; NMB Bchs 482, palate with P^4^–M^3^; NMB Eh 557, dentary with P_4_–M_3_

***Altacreodus magnus* (*Cimolestes magnus*, Lillegraven 1969)**

Formation: Ravenscrag Formation, Frenchman Formation, Hell Creek Formation, Scollard Formation, Lance Formation

Locality: Saskatchewan, Alberta, Montana, Wyoming

Geological Age: Late Cretaceous (Lancian)

Absolute Age: 70–65 Ma

Country: North America

Citation: Lillegraven, 1969; Kielan-Jaworowska et al., 2004; Moore et al., 2014; Fox, 2015

Specimens from Fox, 2015: UALVP 620 (Holotype), dentary with P_4_–M_3;_ UALVP 3793, maxilla with P^4^–M^3^; UALVP 3267, left M^2^; UALVP 3791, dentary with P_3_, P_4_, M_1_–M_3_; UALVP 3754, dentary with P_1_, P_3_, P_4_, M_1_–M_3_;

Specimens from Lillegraven, 1969, Fig. 35 (UALVP 4085b, dP^4^; UALVP 3795, canine; UALVP 2997, P^4^; dP^3^, dP^4^; UALVP 3736, M^3^, UALVP 3793, maxilla with P^4^–M^3^), Fig. 36 (UALVP 3676, lower canine; UALVP 3152, P_1_; UALVP 3791, P_1_–M_3_), Fig. 37 (UALVP 3267, M^2^; UALVP 3781, P^4^; UALVP 3754, P_1_, P_3_–M_3_)

***Anasinopa leakeyi***

Formation: Hiwegi Formation and Kulu Formation

Locality: Rusinga Island, Lake Victoria (Maboko Island, Rousing Site 106, Karugu, Mfwanganu Island)

Geological Age: Burdigalian, Miocene

Absolute Age: 17.8–15 Ma

Country: Kenya

Citation: Savage, 1965; Werdelin, 2010

Specimens observed: BMNH M 19081a (Holotype), maxilla with P^4^, M^1^; BMNH M19081b (Holotype), maxilla with M^1^–M^3^; BMNH M 19081c (Holotype), dentary with P_1_–M_3_; BMNH M 19081d (Holotype), dentary with M_2_, M_3_; BMNH M19081e, dentary with canine and P_4_; KNM-FT 3357, maxilla with dP^3^–dP^4^; KNM-FT 15092, isolated M^1^; KNM-WK 17061, dentary with P_2_–M_3_; KNM-WK 16992, dentary with M_2_; KNM-WK 18197, isolated teeth P^4^, M^3^, M_2_, femoral head, long bone shaft; KNM-RU 52250, dentary with I_1_–M_3_; KNM-RU 2935, dentary with M_1_–M_3_; KNM-RU 2928, M^2^; KNM-RU 2929, right M^2^; KNM-FT 3658, M_1_

***Apterodon gaudryi (=A. flonheimensis; =A. intermedius)***

Formation: Quercy, Mainz Basin, Weisselter Basin

Locality: Espenhain

Geological Age: Late Rupelian (MP22), Early Oligocene

Absolute Age: 32.6–30.9 Ma

Country: Germany

Citation: Lange-Badré and Böhme, 2005; Grohé et al., 2012

Specimens observed: AMNH 12391 (Cast of Holotype), P_4_–M_3_; BSPG 2008.43 (*Apterodon intermedius* holotype), P_4_–M_2_; BMNH M55a, maxilla with M^1^–M^2^

***Apterodon langebadreae***

Formation: Idam Unit, Sarir Tibisti Basin

Locality: 68.19, 69.53, 25, Dur At-Talah

Geological Age: Late Bartonian

Absolute Age: 39 if Late Bartonian (Grohé et al. 2012); Likely 33 and Rupelian (Seiffert, 2010); range used: 37–33 Ma

Country: Libya

Citation: Grohé et al., 2012; Seiffert, 2010

Specimens observed: BMNH M 85297 (Holotype), dentary with C–M_3_; BMNH M 85298 (Holotype), detnary with C, P_2_–M_3_; BMNH M 85300 (Holotype), maxilla with C, P^1^, P^4^–M^3^; BMNH M 85301, maxilla with I^2^–P^2^; BMNH M 85303, maxilla with P^3^; BMNH M 85304, frontal fragment; BMNH M 85307, squamosal fragment; BMNH M 85308, squamosal fragment; BMNH M 85309, sagittal crest; BMNH M 85310, cranial fragment; BMNH M 85312, occipital fragment; BMNH M 85313, cervical vertebra; BMNH M 85315, femur; BMNH M 85315, distal femur; BMNH M 85317, proximal femur; BMNH M 85318, humerus; BMNH M 85319, proximal tibia; BMNH M 85320, distal tibia; BMNH M 85321, distal radius; BMNH M 85322, ulna; BMNH M 85323, proximal radius; BMNH M 85324, metacarpal IV; BMNH M 85325, distal metacarpal IV; BMNH M 85327, phalanx I; BMNH M 85328, phalanx II; BMNH M 85329, phalanx II, BMNH M 85330, rib; BMNH M 85331, distal fibula; BMNH M 85332, innominate fragment; BMNH M 85333, innominate fragment

***Apterodon libyca***

Locality: Gebel Zeltan

Geological Age: Burdigalian, middle Miocene

Absolute Age: 19–14 Ma

Country: Libya

Citation: Morales et al., 2010

Specimens: BMNH M82375, right dentary with M_1_, M_2_, M_3_; BMNH M82376, right dentary with P_3_; BMNH M82378, left dentary with dP_3_, dP_4_; BMNH M82377, right dentary with alveoli and symphysis

***Apterodon macrognathus***

Formation: Qasr el Sagha Formation, Jebel Qatrani Formation

Locality: Quarry A

Geological Age: Early Rupelian

Absolute Age: 33.9–33.7 Ma

Country: Fayum, Egypt

Citation: Holroyd, 1994; Lewis and Morlo, 2010; Seiffert, 2010

Specimens observed: CGM 8982 (Holotype), dentary with P_2_–M_2_; AMNH 13236, cranium with I^1^–M^3^; AMNH 13237, cranium with I^1^–M^3^; CGM 29916, dentary with P_2_–M_3_; DPC 7731, maxilla with canine–M^3^; UCMP 154454, dentaries with canine–M_3_; DPC 7731, maxilla with C–M3; DPC 0959, humerus; DPC 11347, dentary with M_1_–M_2_; SMNS 12643, dentary with canine–M_3_; SMNS 11950 (Holotype), dentary P_4_–M_3_; SMNS 47724, palate with canine–M^3^; SMNS 11267a, dentary with P_4_–M_3_; SMNS 11267b, dentary with M_3_; SMNS 43466, astragalus; SMNS 43467, radius; SMNS 47729, distal humerus, ulnae, tibia; BSPG 1905 XIII 510, dentary with Canine, P_2_, P_4_–M_3_; BSPG 1905 XIII 9, maxilla with P^4^–M^3^; YPM 18160, dentary with canine, P_4_–M_3_; AMNH 13240, dentary with canine–M_3_; AMNH 13247, tibia; AMNH 92794, calcaneum; AMNH 13241, dentary with P_2_–M_3_; BMNH M 8441, astragalus; DPC 1143, M^1^; DPC 2557, dentary with M_1_–M_3_; DPC 4126, maxilla fragment with dP^3^

***Arfia gingerichi***

Formation: Tienen Formation

Locality: Dormaal

Geological Age: MP7, very close to PETM

Absolute Age: 55.6–55.4 Ma

Country: Belgium

Citation: Smith and Smith, 2001; Smith, Rose & Gingerich, 2006

Specimens observed: IRSNB M1275 Holotype, M_3_; IRSNB M1301, P_3_; IRSNB M1302, DP_4_; IRSNB M1303, P_4_; IRSNB M1304, M_1_; IRSNB M1305, M_2_; IRSNB M1306, M_3_; IRSNB M1307, P^3^; IRSNB M1358, DP^4^; IrSNB M1308, P^4^; IRSNB M1309, M^1^; IRSNB M1310, M^2^; IRSNB M1311, M^3^

***Arfia opisthotoma***

Formation: Willwood Formation

Locality: Clarks Fork Basin, Bighorn Basin, Powder River Basin, Piceance Creek Basin

Geological Age: Early Eocene, early to middle Wasatchian (Sandcouleean Wa2 to late Graybullian Wa5)

Absolute Age: 55–53 Ma

Country: USA

Citation: Matthew, 1901; Ivy, 1993; Gunnell, 1998

Specimens observed: AMNH 99, UM 78996, dentary with M_1_–M_3_; UM 69949, dentary with canine, P_2_–M_3_

Specimens from literature: UA 8271, rostrum with canine–M^2^ (Gingerich and Deutsch, 1989)

***Arfia shoshoniensis***

Formation: Willwood Formation

Locality: Clarks Fork Basin, Bighorn Basin, Powder River Basin

Geological Age: Early Eocene, early to middle Wasatchian (early Sandcouleean Wa1 to early Graybullian Wa3)

Absolute Age: 55–53 Ma

Country: USA

Citation: Matthew, 1915; Ivy, 1993; Gunnell, 1998

Specimens observed: AMNH 16158 (Holotype), UM 85935, rostrum with P^1^–M^3^; UM 75186, dentaries with canine, P_2_–M_3_; UM 80487, dentary with P_1_–M_3_; UM 69474, partial skeleton with humeri, vertebral elements, tibia, femora, astragali, calcaneum, metapodials, innominate; UM 75383, radius

Specimens from literature: UM 65502, M^1^–M^2^, M_2_–M_3_ (Gingerich and Deutsch, 1989)

***Boritia duffaudi***

Formation: ?

Locality: La Borie

Geological Age: Early Eocene, MP8 and MP9

Absolute Age: 54–51 Ma

Country: France

Citation: Solé et al., 2014a

Specimen from literature: MHNT.PAL.2010.19.1 (Holotype), P_3_, P_4_, M_1_–M_3_

***Boualitomus marocanensis***

Formation: Sidi Daoui and Recette 4 quarries, lowermost bed I

Locality: Grand Daoui, Ouled Abdoun Basin

Geological Age: earliest Ypresian

Absolute Age: 55.8–54 Ma

Country: Morocco

Citation: Gheerbrant et al., 2006; Seiffert, 2010

Specimens observed from a cast of the holotype: OCP DEK/GE 306 (Holotype), dentary with canine, P_2_–M_3_ (thanks to E. Gheerbrant for creating the cast)

***Brychotherium ephalmos***

Formation: Jebel Qatrani Formation

Locality: L-41

Geological Age: late Priabonian, latest Eocene

Absolute Age: 35–33.9 Ma

Country: Egypt

Citation: Holroyd, 1994; Seiffert, 2010; Present study

Specimens studied: CGM 83750 (Holotype), dentary with C–M_3_; DPC 17627, dentary with P_4_–M_3_; DPC 11990, rostrum with P^4^M^3^; DPC 11569A, dentary with C, P_2_–M_3_; DPC 11569B, dentary with P_2_, P_3_, M_1_–M_3_

***Buhakia moghraensis***

Formation: Moghra Formation

Locality: Wadi Moghra

Geological Age: early Miocene (Burdigalian)

Absolute Age: 18 Ma – 16.8 Ma

Country: Egypt

Citation: Morlo, Miller & El-Barkooky, 2007

Specimens: DPC 8974, dentary with dP_4_, M_1_, M_2_

***Cynohyaenodon cayluxi***

Formation: Quercy, Egerkingen

Locality: Quercy, Egerkingen

Geological Age: MP14, MP18–MP20, Priabonian, Late Eocene

Absolute Age: 48–34 Ma

Country: France

Citation: Solé, 2013; Solé et al., 2014b

Specimens observed: MCZ 8901, cranium with P^3^–M^3^; MCZ 8902, dentary with P_3_–M_3_; UCMP 140651, dentary with P_2_–M_3_ (cast); UCMP 140652, dentary with P_2_–M_2_ (cast); UCMP 140653, dentary with P_2_–M_3_; MNHM Qu 8562, cranium with P^2^–M^3^; MNHM Qu 8566, dentary with P_4_–M_3_; MNHM Qu 8564, dentary with canine–M_2_; MNHM Qu unnumbered, maxilla with P^4^–M^3^; BMNH M 9612, dentary P_2_–M_3_

***Dissopsalis pyroclasticus***

Formation: Ngorora Formation (Locality 2/56) and Kaboor

Locality: Tentatively in (Lewis and Morlo discussion) Kaboor, Fort Ternan, Maboko, Moroto, Napak

Geological Age: Middle Miocene

Absolute Age: 15–9 Ma

Country: Kenya

Citation: Savage, 1965; Barry, 1988; Lewis and Morlo, 2010; Werdelin, 2010

Specimens observed: BMNH M. 19082, dentary with P_4_–M_1_ (Holotype); KNM-MB 25305, maxilla fragment with M^2^; KNM-MJ 13, maxilla fragment with P^4^–M^2^; KNM-FT 13770, M_3_; KNM-MB 8432, M^2^; KNM-FT 15092, M^1^; KNM-BN 1191, M^2^; KNM-FT 3562, dentary with P_2_–P_4_, M_1_–M_2_

***Eomaia scansoria***

Formation: Yixian Formation

Locality: Liaoning Province

Geological Age: Early Cretaceous, middle Barremian

Absolute Age: 129.7–122.1 Ma

Country: China

Citation: Kielan-Jaworowska et al., 2004; Chang et al., 2009; Beck and Lee, 2014

Specimen scored from Kielan-Jaworowska et al., 2004

***Eoproviverra eisenmanni***

Formation: Rians

Locality: Rians

Geological Age: earliest Eocene, MP7

Absolute Age: 55.8–48.6 Ma

Country: France

Citation: Godinot, 1981; Solé et al., 2014a

Specimen scored from Literature: From Godinot, 1981: MNHN.F.RI 400, M_2_; MNHN.F.RI 203, M_3_; MNHN.F.RI 401, M^1^; MNHN.F.RI 362, M^2^; MNHN.F.RI 2014, dentary with M_1_–M_2_

***Eurotherium matthesi***

Formation: Wittenberg Formation (among others)

Locality: Geiseltal (Lutetian, MP11; Germany), Egerkingen y, a+B (Lutetian, ?MP13–Mp14; Switzerland), La Défense (Lutetian, MP13; France), Issel (Lutetian, MP14; France), Aigues-Vives 2 (Lutetian, ?MP13; France)

Geological Age: Lutetian, MP11–MP14

Absolute Age: 48.6–40.4 Ma

Country: Germany, France, Switzerland

Citation: Polly and Lange-Badré, 1993; Solé, Falconnet, and Yves, 2014

Specimens observed: UCMP 140638, dentary with P_1_–M_3_ (Cast, original specimen number not recorded); UCMP 140639, maxilla with P^2^–M^3^ (Cast, original specimen number not recorded); UCMP 140635, astragalus (cast of GMH XIV 3614); UCMP 140634, calcaneum (cast of GMH XIV 2364); GMH XIV 224, P_1_–M_3_; GMH XI-1-1954 (Holotype), dentary with P_2_–M_3_; GMH XIV-3419-1956, cranium with P^2^–M^3^; GMH XIV-1357-1955, cranium with P^2^–M^3^; GMH XIV-3332-1956, dentary with P_3_–M_3_; GMH XIV-3614-1956, astragalus; GMH XIV-2364-1954, calcaneum

***Eurotherium theriodis***

Formation: unnamed karst infillings

Locality: Egerkingen y, a+B (Lutetian, ?MP13–Mp14; Switzerland), Aigues-Vives 2 (Lutetian, ?MP13; France)

Geological Age: Lutetian, MP13

Absolute Age: 48.6–40.4 Ma

Country: France, Switzerland

Citation: Polly and Lange-Badré, 1993; Solé, Falconnet, & Yves, 2014

Specimens observed: UCMP 140647, dentary with P_2_–M_3_ (Cast, original specimen number not noted); NMB Em. 12, cranium with P^1^–M^1^ (Holotype), NMB Em. 14a, cranium with P^3^–P^4^, humerus (Egerkingen, 1915 on card); NMB Em. 14b (Paratype), dentary with M_2_–M_3_; NMB Em. 193 (Paratype), M^2^; NMB En. 247, M^2^; NMB En. 120, dentary with P_3_–M_3_; NMB En. 106, P^4^; NMB En. 140, M^1^; NMB Eh. 536, dentary with M_2_–M_3_

Observed in Solé et al. (2015): MNHN.F.ERH427, right dentary with canine–M_3_; MNHN.F.ERH428, dentary with P_1_, P_2_, P_4_

***Furodon crocheti***

Formation: Glib Zegdou Formation

Locality: HGL 50 and HGL 50 bis, Gour Lazib, Tindouf Province

Geological Age: late Ypresian or middle Lutetian

Absolute Age: 49.3–45.7 Ma

Country: Algeria

Citation: Solé et al., 2014b; Coster et al., 2012

Observed casts of material at MNHM thanks to F. Solé: HGL 50bis-56 (Holotype), dentary with canine base, P_1_–P_3_ alveoli and P_4_–M_3_; HGL 50-410, M_1_; HGL 50-404, M^1^; HGL 50-405, M^1^; HGL 50-407, M^1^

***Galecyon chronius***

Formation: Willwood Formation

Locality: Bighorn Basin, Clarks Fork Basin

Geological Age: Wa-6

Absolute Age: 53–50.3 Ma

Country: USA

Citation: Zack, 2011

Observed from Zack (2011): USNM 487920 (Holotype), petrosal, P^3^ (left), P^4^–M^1^; USNM 511004, dentary with P_2_, P_4_–M_3_; YPM 23341, dentary with P_2_–P_4_; USNM 51190, P_4_; USGS 8769, dentary with M_1_–M_2_; USGS 10284, M_2_; USNM 487920, dentary with C, P_2_–P_3_, M_1_–M_3_; USGS 15956, M^2^

Observed from Zack and Rose (2015): USNM 511004, humerus, ulna, scaphoid, lunate, tibia, astragalus, calcaneum, cuboid

***Galecyon mordax***

Formation: Willwood Formation

Locality: Bighorn Basin, Clarks Fork Basin

Geological Age: Wa-5, Wa-1/2 to Wa-3

Absolute Age: 55.4–53 Ma

Country: USA

Synonym: *Prolimnocyon robustus*

Citation: Zack, 2011

Specimens observed: UM 85887, dentary with canine, P_2_–M_2_; AMNH 16157 (Holotype), canine, P_2_–M_3_

Observed from Zack (2011): USNM 490637, dentary with canine, P_1_–M_1_

Observed from Gingerich and Deutsch (1989): UM 76227, dentary with M_1_–M_3_

Observed from Zack and Rose (2015): USNM 1125, glenoid of left scapula; USNM 1125, innominate; USNM 1125, femur, fibula

***Galecyon morloi***

Formation: Tienen Formation

Locality: Dormaal

Geological Age: MP7, very close to PETM

Absolute Age: 55.6–55.4 Ma

Country: Belgium

Citation: Smith and Smith, 2001; Smith, Rose & Gingerich, 2006

Specimens observed: IRSNB M1314, M_1_; IRSNB M1312, DP_4_; IRSNB M1313, P_4_; IRSNB M1315, M_2_; IRSNB M916, M_3_; IRSNB M1316, P_4_; IRSNB M1317, M^1^ or M^2^

***Galecyon peregrinus***

Formation: Willwood Formation

Locality: Bighorn Basin, Clarks Fork Basin, Powder River Basin

Geological Age: early Wasatchian (Sandcouleean), Wa-0 to Wa-1/2

Absolute Age: 55.4–53 Ma

Country: USA

Citation: Zack, 2011

Observed from Zack (2011): USNM 509676 (Holotype), dentary with P_3_–M_3_; UCMP 217129, M_2_; UCMP 217128, M_1_

***Gazinocyon whitiae***

Formation: Green River Basin, Bighorn Basin, Wind River Basin

Locality: Green River Basin, Bighorn Basin, Wind River Basin

Geological Age: Lostcabianian subage, Wasatchian NALMA, early Eocene

Absolute Age: 53–50.3 Ma

Country: Wyoming, USA

Synonym: *Sinopa vulpecula, Prototomus vulpeculus, Gazinocyon vulpecula*

Citation: Matthew, 1915; Gingerich and Deutsch, 1989; Polly, 1996; Rana et al., 2014

Specimens observed: UCMP 137216, dentary with M_3_, calcaneum, radius, atlas, axis dens, innominate, ulna, humerus, femur, astragalus, M^2^; AMNH 15606, dentary with P_2_–M_3_

Observed from Gingerich and Deutsch (1989): USNM 19347, maxilla with P^2^–M^3^

***Glibzegdouia tabelbalaensis***

Formation: Glib Zegdou Formation

Locality: HGL 10 and HGL 50, Gour Lazib, Tindouf Province

Geological Age: late Ypresian or middle Lutetian

Absolute Age: 49.3–45.7 Ma

Country: Algeria

Citation: Solé et al., 2014b; Coster et al., 2012

Observed casts of material at MNHM thanks to F. Solé: GZC 35 (Holotype), M_2_; HGL 10-15, M^2^; HGL 50-411, M_1_; HGL 50-406, P^3^; HGL 50-408, P^3^

***Hemipsalodon grandis***

Formation: Cypress Hills Formation, Clarno Formation, ?Chadron Formation

Locality: Saskatchewan, Oregon, Wyoming, Texas

Geological Age: Late Duchesnean, Bartonian (“late Eocene–early Oligocene” in Mellett)

Absolute Age: 41.2–37.8 Ma

Country: USA

Citation: Mellet, 1969; Solé et al., 2015

Specimens observed: AMNH 95735 (cast of OMSI 619), cranium with I^2^–M^3^ and dentary with canine, P_3_–M_3_; AMNH 95736, maxilla with canine, P^3^–M^3^; AMNH 10636 (cast of NMC 6497, Holotype), dentary with M_3_; AMNH 95780 (cast of SDSM 6333), dentary with P_3_–M_1_

***Hyaenodon horridus***

Formation: White River Formation, South Dakota (many, see Mellet, 1977)

Locality: Saskatchewan, Wyoming, Montana, Colorado, North Dakota, South Dakota

Geological Age: late Chadronian to late Orellan

Absolute Age: 38–33.3 Ma

Country: USA, Canada

Synonym: *Neohyaenodon horridus, Hyaenodon cruentus*

Citation: Mellet, 1977

Specimens observed: AMNH 39438, cranium with I^1^–M^2^; AMNH 39439, cranium with I^2^, I^3^, dC, P^1^, P^2^ (erupting), dP^3^, dP^4^, M^1^, M^2^ (erupting), dentary with canine, P_2_, dP_3_, dP_4_, M_1_, M_2_; UM 6792, humerus; UM 6786, humerus; AMNH 75704, I^1^–M^2^, dentary with I_1_–M_3_; AMNH 1488, cranium with I^2^–M^2^; AMNH 9809, innominate, sacrum, lumbar vertebrae, femur, tibia, fibula, metapodials, astragalus, ; AMNH 1381, humerus, astragalus, calcaneum; AMNH 75701, humerus, scapula; AMNH 1381, humerus, ulna; AMNH 1175, ulna; MCZ 17395, cranium with I^1^–M^2^, mandible with I_1_–M_3_ (dentary occluded to cranium); MCZ 4739, cranium with I^1^–M^2^ and mandible (occluded) with I_1_–M_3_; UCMP 22788, cranium with P^1^–M^2^; UCMP 158793, cranium with I^3^–M^2^; AMNH unnumbered specimen, cranium, dentaries, skeleton; AMNH 8775, cranium and dentaries (occluded), cervical vertebrae, thoracic vertebrae (T1–T4); YPM 10010, cranium with canine, P^2^–M^2^, mandible with I_2_–M_3_; YPM 10916, articulated pes; YPM 10996, humerus; YPM 11035, ulna; YPM 12656 (*Hyaenodon (Neohyaenodon)* holotype), cranium with I^2^–M^2^ and mandible (occluded) with I_2_–M_3_

**Ergiliyn Dzo *Hyaenodon***

Formation: Ergiliyn Dzo Formation

Locality: Khoer Dzan and Ergiliyn Dzo localities (?)

Geological Age: early Oligocene

Absolute Age: 34–32 Ma

Country: Mongolia

Citation: Lavrov, 1999; Morlo and Nagel, 2006

Specimens observed: PIN 3110-5785, dentary with I_1_–M_3_; PIN 3109-283, maxilla with P^2^–P^3^; PIN 3110-578a, cranium with I^1^–M^2^, humerus, tibia, femur, radius; PIN 3109-83, maxilla with P^2^–P^3^

***Hyaenodon exiguus***

Formation: Euzet-les-Bains (Gard): lower Ludien

Locality: Quercy

Geological Age: late Eocene, Euzet Level Zone, Priabonian

Absolute Age: 37.2–33.9 Ma

Country: France

Citation: Lange-Badré, 1979

Specimens observed: MNHM Qu 8364, dentary with canine, P_4_–M_3_; MNHM Qu 8593, basicranial fragment with auditory bulla; MNHM Qu 8425, P_4_–M_3_; MNHM Qu 8647, maxilla fragment with P^3^–M^2^; MNHM Qu 17662, cranium with erupting teeth exposed dP^2^, dP^3^, dP^4^, P^2^, P^3^, P^4^, M1, M^2^

***Hyaenodon minor***

Formation: Euzet-les-Bains (Gard): lower Ludien

Locality: Fons 4 (Gard); Quercy; Roc de Santa, Spain; Hordle, lower and upper Headon Beds, England; Gosgen Kanal, Switzerland

Geological Age: late Eocene, Euzet Level Zone, Priabonian

Absolute Age: 37.2–33.9 Ma

Country: France, Spain, England, Switzerland

Citation: Lange-Badré, 1979

Specimens observed: MNHM Qu 8649, cranium with P^1^–M^2^; MNHM Qu 8461, maxilla fragment with P^1^; MNHM QU 8470, maxilla with P^4^–M^2^; MNHM Qu 8407, M^2^; MNHM Qu 8406, M^1^; MNHM Qu 8471, dentary with P_3_–M_3_; MNHM Qu 8429, dentary with P_3_–M_3_; MNHM Qu 8557, dentary with canine–P_3_; MNHM Qu 8450, dentary with P_2_–P_4_; MNHM Qu 8419, dentary with M_1_–M_3_; MNHM Qu 8329, rostrum with P^2^–M^2^; MNHN Qu 9981, astragalus

***Hyaenodon neimongoliensis***

Formation: Ulantatal Formation

Locality: Ulantatal, Alxa Zuoqi, Nei Mongol

Geological Age: early Oligocene, Hsandagolian

Absolute Age: 33.9–23.03 Ma

Country: China

Citation: Huang et al., 2002; Rodrigues et al., 2014

Specimens observed: IVPP V12438 (Holotype), dentary with canine, P_2_–M_3_; IVPP V12439, maxilla with P^4^–M^2^; IVPP V12440, dentary with canine, P_2_, P_3_; IVPP V12441, P_2_

***Hyainailouros sulzeri***

Formation:

Locality:

Geological Age: MN 3–MN5

Absolute Age: 18–15 Ma

Country: France, Switzerland, Germany

Citation: Ginsburg, 1980; Morlo, Miller & El-Barkooky, 2007; Solé et al., 2015

Specimens observed: SMNS 1926 I 12, P^4^–M^1^ (Cast of Holotype); BMNH M14000, P^4^ (Cast of specimen from Mösskirsch, Baden); BMNH M13999, M^2^ (Cast of specimen from Mösskirsch, Baden)

Specimens scored from literature: Illustrated in Ginsburg, 1980

***Hyainailouros spp.***

Locality: Meswa Bridge

Geological Age: Early Miocene

Absolute age: 22–15 Ma

Country: Kenya, Uganda, Namibia

Specimens observed: “*Hyainailouros fourtaui*” from Wadi Moghra cast at DPC; KNM-ME-22, M_2_; KNM-ME 13, M_2_; KNM-MB 14051, M_2_; “*Hyainailouros sulzeri*” AD 100’96, M_1_, M_3_ (crypt)

***Indohyaenodon raoi***

Formation: Cambay Shale Formation

Locality: Vastan open-cast lignite mine

Geological Age: lower Eocene, Ypresian, Bumbanian Asian Land Mammal Age

Absolute Age: 54.5 Ma

Country: India

Citation: Rose et al., 2014; Rana et al., 2015

Observed from figures in Rana et al., 2015: GU 1680, rostrum with P^2^–M^3^; GU 767, dentary with P_4_–M_3_ (canine–P_3_); GU 1721, M^1^ or M^2^; GU 321, M_1_; GU 1631, M_1_; GU 652, dentary with P_4_, M_2_, alveoli for canine, P_2_, P_3_; GU 1630, dentary with alveoli for canine, P_2_, P_4_–M_1_; GU 740, ulna; GU 741, tibia; GU 273, tibia; GU 807, calcaneus

***Isohyaenodon andrewsi***

Locality: Muruarot, Ombo, Rusinga, Songhor

Geological Age: Burdigalian, early Miocene

Absolute Age: 20–15 Ma

Country: Kenya

Citation: Savage, 1965; Lewis and Morlo, 2010; Werdelin, 2010

Specimens observed: KNM-SO 1104, dentary with M_3_; KNM-RU 2947 and KNM-RU 2948, M_2_, M_3_; KNM-MO 25, dentary with P_3_, P_4_, M_2_, M_3_

Note on *Isohyaenodon andrewsi*: Van Valen (1967) and Morales et al. (2007) both suggested *Isohyaenodon andrewsi* represents the lower and *Leakitherium* *hiwegi* represents the upper dentition of the same taxon. Holroyd (1999) argued *Isohyaenodon andrewsi* represents the lower dentition of *Metapterodon kaiseri*. The protocones of the *Leakitherium* holotype suggest larger talonid basins are expected on the lower dentition than the very reduced talonids retained on the molars of *Isohyaenodon*. We support the speculation that *Isohyaenodon andrewsi* and *Metapterodon* represent the lower and upper dentition of the same taxon based on the size of the occluding carnassial portions of both the upper and lower dentition. The likely lower dentition of *Leakitherium* is known from Rusinga Island, but remains undescribed. The material is publically accessioned at the KNM.

***Isohyaenodon matthewi***

Formation: Hiwegi Formation, Kulu Formation

Locality: Rusinga Island

Geological Age: Burdigalian, early Miocene

Absolute Age: 20–15 Ma

Country: Kenya

Citation: Savage, 1965; Lewis and Morlo, 2010; Werdelin, 2010

Specimens observed: KNM-SO 8527, M^2^, M^3^; KNM-CA 2792, dentary with M_3_

***Isohyaenodon pilgrimi***

Formation: Hiwegi Formation, Kulu Formation

Locality: Rusinga Island (Kavirondo Gulf), Napak

Geological Age: Burdigalian, early Miocene

Absolute Age: 20–15 Ma

Country: Kenya, Uganda

Citation: Savage, 1965; Lewis and Morlo, 2010; Werdelin, 2010

Specimens observed: BMNH M. 19100a (Holotype), dentary with P_2_–M_3_; BMNH M. 19100b (Holotype), dentary with P_3_–M_3_; BMNH M. 19100c (Holotype), cervical vertebrae; KNM-RU 259, dentary with P_4_–M_3_; KNM-RU 2945, dentary with P_2_, P_4_, M_1_; KNM-RU 2943, P_4_, M_2_, M_3_; KNM-RU 5415, astragalus; KNM-RU 8404, rostrum; KNM-SO 1105, dentary with M_2_; KNM-SO 1668, maxilla fragment with P^4^–M^1^; KNM-SO 5395, M_2_; KNM-SO 5671, dentary with M_1_–M_3_; KNM-SO 8420, dentary with P_4_

***Kerberos langebadrae***

Formation: ?

Locality: Lautrec, Montespieu

Geological Age: MP16, middle Eocene, Bartonian

Absolute Age: 40.4–37.2 Ma

Country: France

Citation: Solé et al., 2015

Specimens observed: MNHN.F.EBA 517 (Holotype), cranium with I^2^–I^3^, P^1^–M^3^; MNHN.F.EBA 518a, dentary with canine, M_2_–M_3_; MNHN.F.EBA 518b, dentary with P_2_–M_3_; MNHN.F.EBA 520, fibula; MNHN.F.EBA 521, astragalus; MNHN.F.EBA 522, calcaneus; MNHN.F.EBA 523, metatarsal 1; MNHN.F.EBA 524, metatarsal II; MNHN.F.EBA 525, metatarsal III; MNHN.F.EBA 526, metatarsal II; MNHN.F.EBA 527, middle phalanx; MNHN.F.EBA 528, middle phalanx

***Koholia atlasense***

Formation: ?

Locality: El Kohol, Saharan Atlases

Geological Age: Ypresian, late early Eocene

Absolute Age: 51.8–51 Ma

Country: Algeria

Citation: Crochet, 1988; Solé et al., 2009; Coster et al., 2012

Observed from Fig. 1 and Fig. 2 in Crochet, 1988

***Kyawdawia lupina***

Formation: ‘Upper Member’ of the Pondaung Formation

Locality: Kdw7, Pondaung area

Geological Age: latest middle Eocene

Absolute Age: 40.1–36.7 Ma

Country: Myanmar

Citation: Egi et al., 2005, Zaw et al., 2014

Specimens observed as casts: AMNH 133542 (cast of holotype NMMP-KU 0042), rostrum with I^2^–I^3^, canine, P^1^, P^4^–M^3^; NMMP-KU 0042 (cast at UCMP), canine cast; NMMP-KU 0784, P_4_; NMMP-KU 0043, M_3_; KMMP-KU 1288, I^3^, canines, P_1_, P_4_, M_1_, M_3_, P^2^, P^3^, M^2^, dentary fragments

Specimens observed from Egi et al., (2005): NMMP-KU 0042, cranium with zygomatic arch fragments; NMMP-KU 0044, I^2^–I^3^; NMMP-KU 1661, I^3^, dentary with P_3_, P_4_, M_2_–M_3_; NMMP-KU 0785, humerus, femoral head, tibia, vertebra, jugal (all fragmentary); NMMP-KU 1288, pisiform, phalanx

***Lahimia selloumi***

Formation: local Thanetian bed IIa, Sidi Chennane quarries

Locality: Ouled Abdoun Basin

Geological Age: ?middle Paleocene–late Paleocene, Selandian

Absolute Age: 61.6–59.2 Ma

Country: Morocco

Citation: Solé et al., 2009; Kocsis et al., 2014

Specimens observed from casts created by E. Gheerbrant for E. Seiffert of OCP DEK/GE 443 (Holotype), dentary with alveoli for canine, P_2_–P_4_, M_1_–M_3_; MNHN PM 56, dentary with M_2_–M_3_; MNHN PM 57, dentary with P_3_ roots, M_1_–M_3_; OCP DEK/GE 442, dentary with M_2_–M_3_ and alveoli of P_2_–M_1_

***Leakitherium hiwegi***

Formation: Hiwegi Formation, Kulu Formation

Locality: Rusinga Island (Kavirondo Gulf), Napak

Geological Age: Burdigalian, early Miocene

Absolute Age: 17.8–15 Ma

Country: Kenya, Uganda

Citation: Savage, 1965; Werdelin, 2010

Specimens observed: BMNH M. 19083 (Holotype), maxilla with M^1^–M^2^; KNM-RU 3119, dentary with P_4_–M_3_, isolated M^1^, M^2^, P_1_– P_4_, canines, cranial fragments including the sagittal crest, occipital region, and zygomatic arches; KNM-RU 2949, maxilla with dP^3^, dP^4^; KNM-RU 17244, astragalus; KNM-RU 4389, P^4^; KNM-RU 8390, P_4_; KNM-RU 17243, M_3_; KNM-RU 17343, humerus; KNM-RU 15182, maxilla with dP^3^, dP^4^

Note on *Leakitherium* *hiwegi*: Van Valen (1967) and Morales et al. (2007) both suggested *Isohyaenodon andrewsi* represents the lower and *Leakitherium* *hiwegi* represents the upper dentition of the same taxon. Holroyd (1999) argued *Isohyaenodon andrewsi* represents the lower dentition of *Metapterodon kaiseri*. The protocones of the *Leakitherium* holotype suggest larger talonid basins are expected on the lower dentition than the very reduced talonids retained on the molars of *Isohyaenodon*. We support the speculation that *Isohyaenodon andrewsi* and *Metapterodon* represent the lower and upper dentition of the same taxon based on the size of the occluding carnassial portions of both the upper and lower dentition. The likely lower dentition of *Leakitherium* is known from Rusinga Island, but remains undescribed. The material is publically accessioned at the KNM.

***Leonhardtina gracilis***

Formation: Geiseltal

Locality: Geiseltal

Geological Age: MP12–MP13

Absolute Age: 48.6–40.4 Ma

Country: Germany

Citation: Solé et al., 2014a

Specimens observed: GMH VI-42-1949 (10237) Holotype, dentary with P_2_–M_3_; GMH VI-712-1951, dentary with I_1_–canine, P_1_ alveoli, P_2_–P_4_, M_2_–M_3_, palate with P^3^–M^3^, astragalus; GMH I-786-1949 (10038), cranium with P^2^–M^3^ and petrosal; GMH 2802, calcaneum; GMH VI-343, dentary with P_3_–M_3_; GMH I-78a-10038, dentary with P_3_, P_4_, M_3_; GMH LVIII-23-1982, dentary with P_2_–P_4_, M_2_–M_3_; GMH XXXV-15-1962, maxilla with P^2^–P^4^

***Lesmesodon behnkeae***

***Lesmesodon edingeri***

Formation: Messel

Locality: Messel (Hessen, Germany)

Geological Age: early middle Eocene (MP 11)

Absolute Age: 48.6–40.4 Ma

Country: Germany

Citation: Morlo and Habersetzer, 1999

Specimens observed: SMF-ME 3843, skeleton with P^3^, dP^4^, M^1^, M^2^ exposed; SMF-ME 1465a, upper skeleton with canine–P_3_, dP_4_, M_1_–M_2_

Specimens also scored from observations made in Morlo and Habersetzer (1999)

***Limnocyon verus***

Formation: Bridger Formation

Locality: Bighorn Basin, Bridger Basin, Uinta Basin

Geological Age: Br1, Br2, Br3, middle Eocene, early to late Bridgerian

Absolute Age: 50.3–46.2 Ma

Country: USA

Citation: Ivy, 1993; Morlo and Gunnell, 2003

Specimens observed from observation: YPM 13095 (Holotype), I^1^–I^3^, P^1^, P^4^–M^2^; YPM 11796, cranium with P^3^–M^2^; AMNH 12155, cranium with I^1^–I^3^ (alveoli), canine, P^1^–M^2^, dentary with canine–M_2_, ulna, radius, humerus, femur, tibia, fibula, astragalus, calcaneum, metapodials

***Maelestes gobiensis***

Formation: Djadokhta Formation

Locality: Ukhaa Tolgod, Mongolia

Geological Age: Campanian, Late Cretaceous

Absolute Age: 75–71 Ma

Country: Mongolia

Citation: Wible et al., 2007; Wible et al., 2009

Specimens observed from literature in Wible et al., 2007 and Wible et al., 2009

***Masrasector aegypticum***

Formation: Jebel Qatrani Formation

Locality: Quarry G

Geological Age: Rupelian

Absolute Age: 31–30.6 Ma

Country: Egypt

Citation: Simons and Gingerich, 1974; Seiffert, 2010

Specimens observed: CGM 30978, dentary with P_3_, M_1_–M_3_ (casts of holotype also observed at UCMP 66312, YPM 20943, AMNH 129736); YPM 30030, maxilla with P^3^–P^4^; YPM 20944, dentary with dP_4_–M_1_

***Masrasector ligabuei***

Formation: Ashawq Formation

Locality: Taqah, Dhofar Province, Sultanate of Oman

Geological Age: Priabonian, latest Eocene; Rupelian, early Oligocene

Absolute Age: 33–30.6 Ma

Country: Oman

Citation: Crochet et al., 1990; Seiffert, 2006

Specimens observed in Crochet et al., 1990: TQ 13 (Holotype), M^1^; TQ 14, M_3_

***Masrasector nananubis***

Formation: Jebel Qatrani Formation

Locality: L-41

Geological Age: late Priabonian, latest Eocene

Absolute Age: 35–33.9 Ma

Country: Egypt

Citation: Borths and Seiffert, in press

Specimens observed: CGM 83736 (Holotype), right dentary with canine, P_2_–M_3_;

CGM field number 96-161, rostrum fragment with P^3^–M^2^; CGM field number 95-281, dentary with P_4_–M_1_; CGM field number 95-109, isolated M^2^; DPC 7704, left dentary with P_2_–M_3_; DPC 8276, rostrum and palate with P^3^–M^3^; DPC 9274, right dentary with canine, P_1_, alveoli for P_2_, P_3_–P_4_, alveoli for M_1_, M_2, 3_; DPC 10358, left dentary with dP_3_, dP_4_, M_1, 2_; DPC 11383, right dentary with C, alveoli for P_1_, P_2_–M_3_; DPC 11359, right dentary with canine, P_1–4_, M_3_; DPC 11990, cranium with P^2^–M^3^; DPC 12157, cranium with alveoli for P^1,^ ^2^, P^3^–M^3^; DPC 12330, right dentary with P_3_–M_1, 2_; DPC 12524A, right dentary with alveoli for P_2, 3_, P_4_–M_3_; DPC 13285, rostral fragment with P^2^–M^3^; DPC 15211, left dentary with C, P_1_–M_3_; DPC 15742, right dentary with canine, P_2_–M_3_; DPC 10831, left distal humerus; DPC 15436, left distal humerus; DPC 11670, left distal humerus, DPC 13837, right maxilla dP^3^, dP^4^, M^1^; DPC 20882, maxilla with dP^3^-dP^4^

***Matthodon tritens***

Formation: Geiseltal

Locality: Geiseltal

Geological Age: MP11

Absolute Age: 48.6–40.4 Ma

Country: Germany

Citation: Solé et al., 2014a

Specimens observed: GMH XIV-1-Franzke 6, dentary with canine, P_2_–M_3_, GMH XIV-739-1957, dentary with P_1_–M_3_; GMH XIV 5107-Franzke 9, maxilla with P^2^–P^4^; GMH XIU-3820, humerus; GMH XIV-2832-1956, dorsal cranium fragment

***Megistotherium osteothlastes*** Savage 1973

Locality: Gebel Zeltan, Wadi Moghra

Geological Age: Burdigalian, middle Miocene

Absolute Age: 19–14 Ma

Country: Libya, Egypt

Citation: Savage, 1973; Werdelin, 2010

Specimens observed: BMNH M26173 (Holotype), cranium with alveoli for I^1^–M^2^ with partial crowns of P^2^, M^2^, M^3^; BMNH 9117, astragalus; DPC 6611, dentary with P_4_–M_3_; BMNH M26518, maxilla with alveoli for M^1^–M^3^; BMNH UB 20580, Metatarsal IV; BMNH L17, metatarsal III; BMNH X87, metatarsal III; BMNH UB 20576, distal humerus; BMNH M26516, premaxilla fragment with I^1^ alveoli; BMNH M21902, atlas; BMNH M26515, cranial fragment with parietals, occipital condyles, basicranium; BMNH M92922, maxilla with roots of P^2^–P^3^

***Metapterodon kaiseri***

Formation: Elisabethfeld

Locality: Elisabethfeld, Rusinga Island

Geological Age: early Miocene, middle Burdigalian

Absolute Age: 20–15 Ma

Country: Namibia, Kenya

Citation: Holroyd, 1999; Werdelin, 2010

Specimens observed: BSPG 1926-X-1 (Holotype), maxilla with P^3^–M^2^; KNM-RU 2951, mandibular symphysis with P_1_, P_2_; KNM-KA 77, maxilla fragment with P^3^–M^3^; KNM-RU 8369, M^2^; KNM-RU 29509, maxilla fragment with P^4^–M^2^

***Metapterodon markgrafi***

Formation: Jebel Qatrani Formation

Locality: Quarry A

Geological Age: early Oligocene, Rupelian

Absolute Age: 33.9–33.7 Ma

Country: Egypt

Citation: Holroyd, 1999

Specimens observed: AMNH 14452, maxilla with P^4^, M^1^, M^2^

***Metapterodon schlosseri***

Formation: Jebel Qatrani Formation

Locality: Quarry V

Geological Age: early Oligocene, Rupelian

Absolute Age: 31–30 Ma

Country: Egypt

Citation: Holroyd, 1999

Specimens observed: AMNH 13262, dentary with P_3_, P_4_, M_1_, M_2_, M_3_; DPC 5664, dentary with P_3_, P_4_, M_1_, M_2_, M_3_; DPC 5431, maxilla with P^4^, M^1^, M^2^

***Metasinopa spp.***

Formation: Jebel Qatrani Formation

Locality: Type locality unknown beyond “upper sequence of Jebel Qatrani”

Geological Age: Rupelian, Fayum A? (V is Where *M. osborni* comes from)

Absolute Age: 33–30 Ma

Country: Egypt

Citation: Holroyd, 1994; Lewis and Morlo, 2010; Seiffert, 2010

Specimens observed: AMNH 14453 (Holotype), dentary with canine, P_2_–M_3_; DPC 10199, maxilla with dP^3^, dP^4^, M^1^

***Minimovellentodon russelli***

Formation: Messel

Locality: Mutigny, France

Geological Age: MP 8 and MP 9, early Eocene

Absolute Age: 55.8–48.6 Ma

Country: France

Citation: Solé et al., 2014a

Specimen observed from Solé et al., 2014a: MHNL 20269998 (Holotype), dentary with P_3_–M_3_

***Mlanyama sugu***

Formation: Unnamed

Locality: Nakwai

Geological Age: latest Oligocene to early Miocene

Absolute Age: 24–20 Ma

Country: Kenya

Citation: Rasmussen and Gutierrez, 2009

Note: Rasmussen and Gutierrez (2009) originally described the holotype as retaining P_1_–M_1_ with P_4_ retained as a highly modified molariform tooth. For the purposes of this study, the tooth position identified by Rasmussen and Gutierrez (2009) as P_4_ is considered dP_4_.

Specimens observed: KNM-NW 46832 (Holotype), dentary with canine alveolus P_2_–M_1_; KNM-NW 46828, P_4_; KNM-NW 46824, P^4^; KNM-NW 46829, P_3_; KNM-NW 46830, P_3_; KNM-NW 46831, M^1^; KNM-NW 46909, M_3_; KNM-NW 46913, M_1_; KNM-NW 46914, M_3_

***Morlodon vellerei***

Formation: ?

Locality: MP8+9, Saint Agnan (Paris Basin, France), MP8+9, Avenay, Condé-en-Brie (France)

Geological Age: Early Eocene, MP8+9, Wasatchian

Absolute Age: 55.8–48.6 Ma

Country: France

Citation: Solé, 2013

Specimens observed: StA 741-L (Holotype), dentary with P_2_–M_2;_ StA 326, M^2^ trigonid

Specimens observe from Solé, 2013: MNHN Condé 65, maxilla fragment with P^4^–M^3^

***Pakakali rukwaensis***

Formation: Nsungwe Formation

Locality: Nsungwe 2

Geological Age: late Oligocene

Absolute Age: 24.95 Ma

Country: Tanzania

Citation: Stevens et al., 2013

Specimen observed: RRBP 09088, maxilla fragment with dP^3^ crown, dP^4^–M^1^ alveoli

***Orienspterodon dahkoensis***

Formation: Rencun Member, lower part of Heti Formation in China, Upper member of Pondaung Formation in Myanmar

Locality: Lunan Basin, Yunan Province, southern China; Eastern side of Pondaung Range, central Myanmar

Geological Age: late middle Eocene

Absolute Age: 42–39 Ma

Country: China, Myanmar

Citation: Egi et al., 2007

Specimens observed: PGM V1297, dentary with P_2_–M_3_; AMNH 122028 (cast of IVPP specimen), M_3_; NMMP-KU 0261 (cast observed at UCMP), dentary with P_2_–M_1_; NMMP-KU 0262, M_1_ and M_2_ trigonids; NMMP-KU 0304 (cast observe at UCMP), maxilla with roots of P^3^–P^4^, M^1^

Specimens observed in Egi et al., 2007: NMMP-KU 1628, proximal metacarpal II, metatarsal III, metatarsal IV

***Oxyaenoides bicuspidens***

Formation: Geiseltal

Locality: Geiseltal

Geological Age: MP11

Absolute Age: 48.6–40.4 Ma

Country: Germany

Citation: Solé et al., 2014a; Solé, 2015

Specimens observed: GMH XIV-2848-1955 (Holotype, cast also available as UCMP 140637), dentary with P_2_–M_3_; GMH XV-1143-1957, calcaneum; GMH XIV-2944-Franzke 9, maxilla with alveoli for P^1^–P^4^, crowns of M^1^–M^2^; GMH XIV-2909-1954, dentary with M_1_–M_3_; GMH XIV-2910-Franzke 12, premaxilla fragment and maxilla fragment with M^1^–M^2^; GMH XIV-291, P_4_; GMH XXXVII-174-1970, femur; GMH XIV-2810-1954, humerus; GMH XIV-456-1956, femur

***Oxyaenoides lindgreni***

Formation: Cuis, Mancy

Locality: Cuis, Mancy

Geological Age: MP10

Absolute Age: 51–48.6 Ma

Country: France

Citation: Solé et al., 2014a; Solé, 2015

Specimens observed: MNHN.F.L-49-MA (Holotype), dentary with P_4_, M_2_, M_3_ (casts at MCZ 21254, UCMP 107092); MNHN MA 14826, dentary with P_3_–P_4_; MNHN.F.L-23-Cuis, M^1^; MNHN.F.MA.14833, M_1_

Observed in Solé et al., 2014a

***Paratritemnodon indicus***

Formation: Subathu Formation, upper part

Locality: Outer Himalaya, near Village Fiji on the Metka-Mohgala Road, Rajauri District

Geological Age: Late Early to early Middle Eocene

Absolute Age: 49–44.5 Ma

Country: India

Citation: Kumar, 1992; Rana et al., 2015

Observed from Fig. 2 (WIF/A 1103, palate with canine–M^3^) and Fig. 3 (WIF/A 1102, dentary with P_3_–M_3_) in Kumar (1992)

***Paroxyaena galliae***

Formation: Quercy

Locality: Quercy, France

Geological Age: middle Eocene, Bartonian

Absolute Age: 41.2–37.8 Ma

Country: France

Citation: Lange-Badré, 1979; Solé et al., 2015

Specimens observed: MNHM Qu 8735, dentary with P_4_–M_3_; BSPG 1879-XV-33, maxilla with M^1^, M^2^

Specimens observed observed from Lange-Badré, 1979

***Paroxyaena pavlovi***

Formation: Specific locality unknown

Locality: Quercy, France

Geological Age: late Eocene, Priabonian, MP16

Absolute Age: 40.4–37.2 Ma

Country: France

Citation: Lavrov, 2007; Solé et al., 2015

Observed from cast of GGM Ca-300 courtesy of A. Lavrov

Observed from literature in Figs. 1–4, GGM Ca-300, cranium with dP^3^, dP^4^, M^1^

***Parvagula palulae***

Formation: ?

Locality: Palette, Provence, Bouches-du-Rhone; Fornes, Minervois, Hérau

Geological Age: Early Eocene (not more specific in Solé et al., 2015, Ypresian age used here)

Absolute Age: 56–47.8 Ma

Country: France

Citation: Godinot et al., 1987; Solé et al., 2015

Specimens observed: UM/PAT 4 (Holotype, cast observed at MNHM), dentary with P_2_–M_1_

Specimens observed in Solé et al., 2015: UM/FNR 52, trigonid of M_1_; UM/FNR 53, dentary fragment with P_4_; UM/FDN 153, trigonid of M_1_

***Preregidens langebadrae***

Formation: Argiles rutilantes d’Issel et de Saint-Papoul Formation

Locality: Saint-Papoul, Aude, Languedoc-Roussillon

Geological Age: early Eocene, Ypresian, MP8 and MP9

Absolute Age: 55.8–48.6 Ma

Country: France

Citation: Solé et al., 2015

Specimens observed in Solé et al. (2015): MNHN.F.SPPXX1, dentary with P_1_, P_2_, P_3_ alveoli, P_4_, M_1_– M_3_

***Prolimnocyon atavus***

Formation: Willwood Formation

Locality: Clark’s Fork Basin, Bighorn Basin, Powder River Basin, Piceance Creek Basin, San Juan Basin, Washakie Basin

Geological Age: early Eocene, Wasatchian, Wa3 to Wa6

Absolute Age: 53–50 Ma

Country: USA

Citation: Gebo and Rose, 1993; Gunnell, 1998

Specimens observed: DPC 5364, maxilla with P^4^–M^3^, dentary with P_1_–M_3_, scapula fragment, humerus, radius, ulna, ungula phalanx, innominate, femur, tibia, fibula fragment, calcaneum, astragalus, cuboid, metatarsals, vertebrae

Also referenced illustrations in Gebo and Rose (1993)

***Prolimnocyon chowi***

Formation: Nomogen Formation

Locality: Bayan Ulan, Inner Mongolia

Geological Age: late Paleocene

Absolute Age: 57–55.3 Ma

Country: China

Citation: Meng et al., 1998

Specimens observed from figures in Meng et al. (1998)

***Propterodon morrisi***

Formation: Irdin Manha Beds, Iren Dabasu Basin

Locality: “23 miles south of Iren Dabasu”

Geological Age: middle middle Eocene, Irdinmanhan Age

Absolute Age: 46–43 Ma

Country: Mongolia

Citation: Matthew and Granger, 1924

Specimens observed: 19160 (Holotype), dentary with P_2_, dP_4_, M_2_; AMNH 21553, dentary with P_2_, M_1_–M_3_; AMNH 95776, dentary with canine, P_2_–M_2_; AMNH 96384, dentary with P_2,_ roots of P_3_, P_4_–M_2_; AMNH 95777, dentary with P_2_–M_3_ PIN 71-73, P_3_, M_2_, M_3_ trigonid, dP_4_, M^2^

***Propterodon tongi***

Formation: Hedi Formation, Yuli member

Locality:Huoshipo, Guojia Village, Wangmao Town, Yuanqu, Shanxi

Geological Age: middle middle Eocene, Irdinmanhan Age

Absolute Age: 46–43 Ma

Country: China

Citation: Liu et al., 2002

Specimens observed: IVPP V12612 (Holotype), dentary with P_1_–M_3_

***Prototomus minimus***

Formation: Tienen Formation

Locality: Dormaal

Geological Age: MP7

Absolute Age: 55.8–48.6 Ma

Country: Belgium

Citation: Smith and Smith, 2001

Specimens observed: IRSNB M1287 (Holotype), M_1_; IRSNB M 1286, P_4_; IRSNB M 1288, M_2_; IRSNB M 1289, M_3_; IRSNB M 1290, P_4_; IRSNB M 1291, M^1^; IRSNB M 1292, M^2^; IRSNB M 1293, M^3^; IRSNB M 1294, edentulous dentary; IRSNB M 1295, edentulous dentary

***Prototomus phobos***

Formation: Many (Willwood Formation, Bridger Formation etc.)

Locality: Clarks Fork Basin, Bighorn Basin, Powder River Basin

Geological Age: Early to Middle Eocene, earliest Wasatchian Wa0 to earliest Bridgerian BR0

Absolute Age: 55–46 Ma

Country: USA, Europe

Citation: Gingerich and Deutsch, 1989; Ivy, 1993; Zack, 2011; Solé et al., 2014a

Specimens observed: YPM-PU 13019 (Holotype), cranium with I^1^–I^2^, P^1^–P^2^, P^4^–M^3^ and dentaries with C–M_3_; UM 68075, dentary with C, P_3_, and portions of M_1_–M_3_; UM 74134, maxilla with M^1^–M^3^, astragalus, calcaneum, humerus

***Proviverra typica***

Formation: Geiseltal level XXXVI

Locality: Geiseltal, Germany

Geological Age: Geiseltalian, Lutetian, Middle Eocene

Absolute Age: 46–41 Ma

Country: Germany

Citation: Solé et al., 2014a

Specimens observed: NMB Em 18 (Holotype), cranium with alveoli of canine, roots of P^1^, P^4^, M^1^, M^2^ (protocone), M^3^; NMB Ek 30 (cast also viewed: UCMP 140643), maxilla with P^4^–M^3^; NMB Eh 561 (cast also viewed: UCMP 140642), dentary with P_4_, M_2_–M_3_; GMH XXXVI-519 (cast also viewed: UCMP 140641), dentary with P_4_–M_2_; GMH XXXVII-136-1964, dentary with P_2_–P_3_, fragments of P_4_, M_1_, fragments of M_2_–M_3_; GMH XXXVI-20-1962, dentary with P_3_–P_4_, M_2_ (talonid)–M_3_; GMH XLI-309-1968, dentary with P_4_–M_1_; NMB 162, maxilla with P^4^–M^2^; NMB Eh. 191, P_3_, M_1_–M_3_

**“*Pterodon*” *africanus*** Andrews 1903

Formation: Jebel Qatrani Formation

Locality: Quarry A, lower sequence, Fayum Depression

Geological Age: early Oligocene

Absolute Age: 33.9–33.7 Ma

Country: Egypt

Citation: Holroyd, 1994; Holroyd, 1999; Seiffert, 2010

Specimens observed: BSPG 1905-XIII-8 (Holotype), dentary with canine–M_3_; AMNH 13251, maxilla with P^2^–P^3^, fragmentary P^4^–M^2^; CGM 8897 (cast at BMNH M8887), femur; CGM 8898, humerus; UCMP 41475, maxilla with M^2^; SMNS 43470, calcaneum; SMNS 11575, cranium with incisor alveoli, canine, P^2^–M^3^, posterior portion of cranium very heavily reconstructed; SMNS 43471, tibia; BMNH M8503, dentary with P_2_–M_3_; BMNH 21897, palate with P^2^–M^2^; BMNH M8445, proximal femur; BMNH M9475, astragalus; BMNH M9473, axis fragment; BMNH M9472, axis; BMNH 9472, atlas; BMNH 9472, cervical vertebra; BMNH 8446, vertebra

***Pterodon dasyuroides*** de Blainville 1839

Formation: Quercy

Locality: Paris Basin

Geological Age: MP18–MP20, Priabonian, Late Eocene

Absolute Age: 37.2–33.9 Ma

Country: Paris

Citation: Lange-Badré, 1979; Solé et al., 2014a; Solé et al. 2015

Specimens observed: MNHM 1903-20.Qu.8652 (Holotype), cranium with Canine–M^3^; MNHM 1893-11.Qu 8301, dentary with I_3_, P_1_–M_3_ and cranium with I^2^, canine, P^2^–M^3^;MNHM 1893-11.Qu 8304, cranium with P^2^–M^3^; MNHM 1882-18.Qu 8803, dentary with P_4_–M_3_; MNHM 1903-80.Qu 8669, rostrum with I^1^–I^2^, canine, P^2^–M^3^; MNHM 1903-20.Qu 8631, basicranium; MNHM 1893-11.Qu 8736, dentary with canines, P_2_ –M_3_; MNHM Qu 8734, maxilla with canine–M^3^; MNHM 1875-931.Qu 8787, maxilla with P^3^–M^3^; MNHM 1903-20.Qu 10071, calcaneum; MCZ 8912, dentary with P_2_–M_3_; MCZ 8911, maxilla with P^3^–M^2^; BSPG 1879 1879-XV-32, maxilla with P^4^–M^3^; BSPG 1961-XVII-19, maxilla fragment with M^2^; BSPG 1959-IX-4, maxilla with M^1^–M^2^; BMNH M27578, maxilla with P^2^, P^4^, M^1^–M^2^; BMNH 26757, maxilla with P^4^–M^3^; BSPG 1879 XV 642, dP^3^

**“*Pterodon*” *phiomensis*** Osborn 1909

Formation: Jebel Qatrani Formation

Locality: Quarry A, Fayum Depression

Geological Age: early Oligocene

Absolute Age: 33.9–33.7 Ma

Country: Egypt

Citation: Holroyd, 1994; Holroyd, 1999; Seiffert, 2010

Specimens observed: AMNH 13253 (Holotype), P_2_–M_3_; AMNH 13254, dentary with P_2_–M_3_

***“Pterodon” syrtos***

Formation: Jebel Qatrani Formation

Locality: Quarry M (Fayum Depression)

Geological Age: Rupelian, Younger than G and V (in turn older than A and B)

Absolute Age: 30–29.2 Ma

Country: Egypt

Citation: Holroyd, 1999

Specimen observed from Holroyd, 1999: CGM FN 84-244, maxilla with P^3^, P^4^, M^1^, M^2^

***Pyrocyon strenuus*** Gingerich and Deutsch 1989

Formation: San Jose Formation

Locality: San Jose Basin, Bighorn Basin, Wind River, Green River

Geological Age: Early to Middle Eocene, middle Wasatchian (Graybullian) Wa3 through Bridgerian (Br1) (*P. multicuspis* is early Eocene, middle to late Wasatchian (late Graybullian Wa5) to Lostcabinian (Wa7)

Absolute Age: 55.8–50.3 Ma

Country: USA

Citation: Ivy, 1993; Gingerich and Deutsch, 1989

Specimens observed: USNM 1023 (Holotype), dentary with canine, P_2_–M_3_; AMNH 15234, maxilla with canine–M^3^ and dentary with canine–M_3_; UM 21186, dentary with P_2_, P_4_–M_3_

***Quasiapterodon minutus***

Formation: Jebel Qatrani Formation, Quarry M

Locality: Fayum Depression

Geological Age: Rupelian, Younger than G and V (in turn older than A and B)

Absolute Age: 30–29.2 Ma

Country: Egypt

Citation: Lavrov, 1999; Grohé et al., 2012

Specimens observed: SMNS with no number (Holotype, cast of type also available at UCMP 140656), dentary with part of P_4_, M_1_–M_3_; DPC 2948, maxilla with P^3^–M^3^; DPC 8288, maxilla with P^4^–M^1^; DPC 7314, maxilla with P^3^–M^1^; DPC 21473, dentary with P_4_–M_3_; DPC 3154, dentary with canine, P_2_–M_3_; DPC 201431, dentary with canine, P_3_–M_3_; DPC 5022, dentary with M_1_–M_2_; DPC 2949, dentary with P_4_–M_3_;

***Quercytherium simplicidens***

Formation: Quercy phosphorites

Locality: Quercy phosphorites

Geological Age: early late Eocene

Absolute Age: 37.2–33.9 Ma

Country: France

Citation: Lange-Badré, 1979; Solé, et al., 2014a

Specimens observed: MNHN unnumbered right dentary with P_2_–M_3_; MNHN.F.Qu8559, dentary with P_4_, portions of M_1_–M_3_; MNHN.1962-35, dentary with M_2_–M_3_; MNHN.1893-11, left dentary with P_2_–P_4_, M_3_; MNHN.F.Qu8645, dentary with M_2_–M_3_; MNHN.F.Qu8649, cranium with P^2^, P^4^, M^2^, M^3^

***Quercytherium tenebrosum***

Formation: See localities

Locality: Euzet-les-Bains (Gard), Quercy phosphorites

Geological Age: early late Eocene

Absolute Age: 40–33.9 Ma

Country: France

Citation: Lange-Badré, 1979; Solé, et al., 2014

Specimens observed: MNHN.F.Qu8644 (Holotype), dentary with P_2_–M_3_; MNHN.F.Qu8643, dentary with P_2_–M_3_; MNHN.F.Qu8646, maxilla with P^1^–M^3^

“***Sinopa” ethiopica***

Formation: Jebel Qatrani Formation

Locality: Quarry A, Fayum Depression

Geological Age: early Oligocene

Absolute Age: 33.9–33.7 Ma

Country: Egypt

Citation: Holroyd, 1994; Seiffert, 2010

Specimens observed: Cast of holotype CGM 10193 at BNHM M9260, dentary with P_4_–M_3_

***Sinopa grangeri***

Formation: lower Bridger (B3?) Formation

Locality: Uinta County, Wyoming, Bridger Basin, Uinta Basin

Geological Age: Middle Eocene, Early to late Bridgerian (Br1 to Br3)

Absolute Age: 50.3–46.2 Ma

Country: USA

Citation: Matthew, 1906; Ivy, 1993

Specimens observed: USNM 5341 (Holotype), cranium with Canine–P^2^, P^4^–M^3^, dentaries with canines–M_3_, cervical, thoracic, lumbar, caudal vertebral series, scapula, humerus, ulna, radius, carpals, metacarpals, innominate, femur, tibia, fibula, astragalus, calcaneum, metatarsals (mounted at USNM making it difficult to observe morphology so observations were supplemented by figures in Matthew, 1906)

***Sinopa jilinia***

Formation: Member III, Huadian Formation

Locality: Huadian basin, Huadian County, Jilin Province

Geological Age: middle Eocene, Yuanquan (Uintan)

Absolute Age: 46.2–40.4 Ma

Country: China

Citation: Morlo et al., 2014

Specimen observed from Morlo et al. (2014): RCPS-CAMHD06-001, dentary with P_2_–P_3_, roots of P_4_, M_1_–M_3_ (cast available at SMF)

***Teratodon spekei***

***Teratodon enigmae***

Formation: ?

Locality: Koru, Songhor (*T. enigmae* from Songhor only), and Rusinga

Geological Age: Burdigalian, Early Miocene

Absolute Age: 20–15 Ma

Country: Kenya

Citation: Savage, 1965; Werdelin, 2010

Specimens observed: AMNH 56429 (cast of *T. enigmae* holotype BMNH M19089, cast also available as UCMP 77538), dentary with roots of P_2_–P_4_, alveoli of M_1_–M_3_; AMNH 56428 (cast of *T. enigmae* holotype, cast also at UCMP 77535), maxilla with P^2^–M^3^; AMNH 56425 (original BNMH M14310, cast of *T. spekei* holotype), maxilla with canine, P^2^; AMNH 56427 (cast of *T. spekei* paratype, also UCMP 77543), dentary with P^2^–P^3^; AMNH 56426 (cast of BMNH M14216, also available at UCMP 77541), dentary with M_2_–M_3_; AMNH 56424 (cast of *T. spekei* holotype BMNH M14307, also available at UCMP 77542), maxilla with P^4^–M^2^; UCMP 77544 (cast of BMNH M14308), dentary with P_2_–P_3_; KNM-SO 85, M_2_; KNM-SO 1110, dentary with P_2_–P_3_, M_1_–M_3_; KNM-SO 1109, dentary with M_1_–M_3_; KNM-SO 1111, mandibular condyles, P_1_–P_2_; KNM-SO 5118, P^4^; KNM-CA 311, P_3_; KNM-CA 1915, dentary with P_3_–P_4_; KNM-RU 14769, maxilla with canine–P^4^, dentary with P_2_–P_4_, roots of M_1_–M_3_; KNM-ME 29, dentary with P_2_–M_2_; KNM-LG 679, maxilla with P^4^–M^2^

***Thinocyon velox***

Formation: Grizzly Buttes, Blacks Fork Member, Bridger Formation (Type)

Locality: Bridgerian biochrons Br-1b, Br-2 northern and southern Green River Basin, Br-3 Washakie Basin, Wyoming

Geological Age: middle Eocene, Br1–Br3

Absolute Age: 50.3–46.2 Ma

Country: USA

Citation: Morlo and Gunnell, 2003

Specimens observed: YPM 11797, dentary with canine, P_1_, fragmentary P_2_–P_3_, P_4_, partial M_1_–M_2_; AMNH 13081, cranium with P^1^–M^2^, dentary with P_2_–M_2_, ulna, radius, metacarpals, sacrum; AMNH 140007, cranium and dentary (occluded) with I^1^–M^2^ and I_1_–M_2_; AMNH 11524, femur, tibia; AMNH 12154, atlas, axis, cervical, thoracic, and lumbar vertebrae, humerus, radius, ulna, carpals, femur, tibia, metapodials, astragalus, calcaneum

Specimens observed rom Morlo and Gunnell (2003): GMUW 3059, humerus, astragalus, calcaneum

***Tinerhodon disputatum***

Formation: Adrar Mgorn 1 and Ihadjamene

Locality: Ouarzazate Basin

Geological Age: Thanetian, Late Paleocene

Absolute Age: 56.5–55.8 Ma

Country: Morocco

Citation: Gheerbrant, et al., 2006; Seiffert, 2010

Specimens from literature (Gheerbrant et al., 2006): THR 192 (Holotype), M_3_; THR 294, P_2_; THR 292, P_4_; THR 193, M_2_; THR 192, M_3_; THR 313, P_3_; IDJ 1, M_1_ or M_2_; THR 111, M_1_ or M_2_

***Tritemnodon agilis***

Formation: Bridger Formation

Locality: Type from Grizzly Buttes, Uinta County; Bighorn basin, Bridger basin, Uinta basin

Geological Age: middle Eocene, early to middle Bridgerian (Br1 to Br2)

Absolute Age: 50.3–46.2 Ma

Country: USA

Citation: Matthew, 1909; Ivy, 1993

Specimens observed: AMNH 11536, calcaneum, astragalus, femur, tibia,

fibula, atlas, innominate, sacrum, vertebrae, metapodials, metatrasals, phalanges, maxilla with canines–M^3^, dentaries with canines–M_3_; AMNH 12636, cranium with canines–M^3^; dentaries with canine–M_3_; complete cervical, thoracic, lumbar and partial caudal vertebral series, scapulae, humerus, ulna, radius, metapodials, carpals, phalanges, innominate, femur, tibia, fibula, metatarsals, astragalus, calcaneum, metapodials, phalanges

**Carnivora**

***Africanictis hyaenoides***

Family: Viverridae

Country: Kenya, Namibia

Absolute Age: 20–17 Ma

Citation: Morales et al., 2003

Specimens: AD 241’99, M_1_

***Africanictis meini***

Family: Viverridae

Country: Kenya, Namibia

Absolute Age: 20–17 Ma

Citation: Morales et al., 2003

Specimens: AD 119’98, dentary with P_2_, P_3_, P_4_, M_1_; AD 421’97, dentary with P_2_, P_3_, P_4_, M_1_; AD 118’98, dentary with P_4_, M_1_

***Africanictis schmidtkittleri***

Family: Viverridae

Country: Kenya, Namibia

Locality: Chamtwara, Legetet

Absolute Age: 20–17 Ma

Specimens: KNM-CA 303, P^4^; KNM-LG 1246, maxilla fragment with M^1^-M^2^

***Afrocyon burolleti***

Family: Amphicyonidae

Country: Libya

Locality: Gebel Zelten

Absolute Age: 19–15 Ma

Specimens: Cast of Holotype BMNH 1961-5-7; BMNH M82373, dentary with P_4_, M_1_, M_2_

***Afrosmilus turkanae***

Family: Barbourofelidae

Country: Kenya, Namibia

Locality: Muruorot

Absolute Age: 20–15.4 Ma

Specimens: KNM-MO 15929, dentary with P_2_, P_3_, M_1_, KNM-RU 15986, maxilla fragment with M^1^

***Amphicyon giganteus***

Family: Amphicyonidae

Country: Libya, Namibia

Locality: Kipsaraman, Ngorora Member

Absolute Age: 19–15 Ma

Specimens: PQAD 1520, dentary with P_4_, M_1_, M_2_

***Cynelos euryodon***

Family: Amphicyonidae

Country: Egypt, Kenya, Uganda

Locality: Mfwangano, Songhor,

Absolute Age: 20.5–16 Ma

Specimens: KNM-MW 17337, dentary with M_1_; KNM-SO 1107, M_1_; KNM-KT 31244, M_1_; KNM-SO 5668, M_1_; BMNH M19085, M_1_

***Diamantofelis ferox***

Family: Felidae

Country: Namibia

Locality: Arrisdrift

Absolute Age: 17.5–17 Ma

Citation: Morales et al., 2003

Specimen: AD 98’95, P_3_, P_4_, M_1_

***Ginsburgsmilus napakensis***

Family: Barbourofelidae

Country: Uganda

Locality: Napak

Absolute Age: 20–19 Ma

Citation: Morlo et al., 2004

Specimen: UM Nap IX 1966, maxilla with P^3^, P^4^, M^1^

***Herpestides aegypticus***

Family: Viverridae

Country: Egypt

Locality: Wadi Moghra

Absolute Age: 18–10 Ma

Citation: Morlo et al., 2007

Specimens: DPC 5975, dentary with P_4_, M_1_

***Herpestides aequatorialis***

Family: Viverridae

Country: Kenya, Ethiopia

Locality: Rusinga

Aboslute Age: 18–10 Ma

Specimens: KNM-RU 2901, dentary fragment with P_4_, M_1_; KNM-RU 2908, dentary with M_1_; KNM-RU 2914, dentary with P_4_, M_1_; KNM-RU 2924, M_1_

***Ketketictis solida***

Family: Viverridae

Country: Egypt

Locality: Wadi Moghra

Absolute Age: 18–10 Ma

Citation: Morlo et al., 2007

Specimens: DPC 5982, M_1_

***Kenyalutra songhorensis***

Family: Mustelidae

Country: Kenya

Locality: Songhor

Absolute Age: 20–19 Ma

Specimens: KNM-SO 1376, M_1_

***Kichechia zamanae***

Family: Herpestidae

Country: Kenya, Uganda

Locality: Moruorot, Rusinga

Absolute Age: 20.5–16.4 Ma

Specimens: KNM-MO 24, dentary with P_2_, P_3_, P_4_, M_1_; KNM-MO 18100, M_1_; KNM-RU 2902, M_1_; KNM-RU 2906, dentary with P_4_, M_1_; KNM-RU 2909, dentary with P_4_, M_1_; KNM-RU 2910, dentary with P_4_, M_1_; KNM-RU 2913, dentary with M_1_; KNM-RU 2923, dentary with P_4_, M_1_;

***Legetetia nandii***

Family: Herpestidae

Country: Kenya

Locality: Legetet, Koru, Songhor

Absolute Age: 20–19 Ma

Specimens: KNM-LG 725, M_1_; KNM-LG 789, M_1_; KNM-LG 802, M_1_; KNM-LG 2405, M_1_; KNM-KO 102, maxilla with P^4^, M^1^, M^2^; KNM-SO 1379, dentary with M_1_, M_2_; KNM-SO 1409, M_1_; KNM-SO 15963, maxilla with M^1^, M^2^;

***Leptoplesictis mbitensis***

Family: Herpestidae

Country: Kenya

Locality: Rusinga

Absolute Age: 20.5–16.4 Ma

Specimens: KNM-RU 15991, dentary with M_1_; KNM-RU 15994, dentary with P_4_, M_1_

***Leptoplesictis namibiensis***

Family: Herpestidae

Country: Namibia

Locality: Langental

Absolute Age: 20.5–16.4 Ma

Citation: Morales et al., 2008

Specimens: LT 50’07, M_1_

***Leptoplesictis rangwai***

Family: Herpestidae

Country: Kenya, Namibia

Locality: Legetetia, Rusinga

Absolute Age: 20.5–16.4 Ma

Specimens: KNM-LG 1555, dentary with P_2_, P_3_, P_4_, M_1_; KNM-RU 2900, dentary with M_1_; KNM-RU 15990, dentary with P_4_, M_1_, M_2_

***Leptoplesictis senutae***

Family: Herpestidae

Country: Namibia

Locality: Grillental, Sperrgebiet

Absolute Age: 20.5–16.4 Ma

Specimens: GT6-1’06, denary with P_1_, P_2_, P_3_, P_4_, M_1_, M_2_

***Luogale rusingensis***

Family: Mustelidae

Country: Kenya

Locality: Rusinga

Absolute Age: 18–17 Ma

Specimens: KNM-RU 15981, dentary with M_1_, M_2_

***Mioprionodon hodopeus***

Family: Viverridae

Country: Kenya

Locality: Nakwai

Absolute Age: 23 Ma

Citation: Rasmussen and Gutierrez, 2009

Specimens: KNM-NW 46827, dentary with M_1_, M_2_

***Mioprionodon pickfordi***

Family: Viverridae

Country: Kenya

Locality: Songhor

Absolute Age: 20–19 Ma

Specimens: KNM-SO 15971, dentary with M_1_, M_2_; KNM-SO 1378, maxilla with P^4^, M^1^

***Moghradictis nedjema***

Family: Viverridae

Country: Egypt

Locality: Wadi Moghra

Absolute Age: 18–17 Ma

Citation: Morlo et al., 2007

Specimens: DPC 4069, dentary with P_4_, M_1_

***Namibictis senuti***

Family: Mustelidae

Country: Namibia

Locality: Arrisdrift, Sperrgebiet

Absolute Age: 17.5–17 Ma

Citation: Morales et al., 2003

Specimens: AD 529’99, dentary with P_3_, P_4_, M_1_; AD 201’97, dentary with M_1_; AD 614’98, dentary with P_4_, M_1_; PQAD 614, dentary with canine, P_2_, P_3_, P_4_, M_1_

***Namafelis minor***

Family: Felidae

Country: Namibia

Locality: Arrisdrift, Sperrgebiet

Absolute Age: 17.5–17 Ma

Citation: Morales et al., 2003

Specimens: AD 99’95, dentary with P_3_, P_4_, M_1_

***Orangictis gariepensis***

Family: Viverridae

Country: Namibia

Locality: Arrisdrift, Sperrgebiet

Absolute Age: 17.5–17 Ma

Citation: Morales et al., 2001

Specimens: AD 613’98, dentary with C–M_2_

***Stenoplesictis muhoronii***

Family: Viverridae

Country: Kenya

Locality: Rusinga, Songhor

Absolute Age: 20–17 Ma

Specimens: KNM-RU 15989, dentary with M_1_; KNM-SO 15970, maxilla with P^4^, M^1^, M^2^

***Syrtosmilus syrtensis***

Family: Barbourofelidae

Country: Libya

Locality: Gebel Zelten

Absolute Age: 19–15 Ma

Specimens: Cast of holotype at BMNH

***Ysengrinia ginsburgi***

Family: Amphicyonidae

Country: Namibia

Geological Age: Middle Miocene

Absolute Age: 20–17 Ma

Specimens measured from Morales et al., 2003: PQAD 133, dentary with P_4_, M_1_, M_2_, M_3_; AD 227’99, M_1_

**Prionogalidae**

***Prionogale breviceps***

Locality: Chamtwara, Legetet, Napak, Songhor

Geological Age: early Miocene

Absolute Age: 20-19 Ma

Country: Kenya

Citation: Schmidt-Kittler and Heizmann, 1991

Specimen observed: KNM-SO 1431 (Holotype), maxilla fragment wit dP^3^, dP^4^, M^1^; KNM-SO 22957, dentary with two incisor roots, canine root, dP_4_? fragment; KNM-CA 2112, dentary with P_3_, P_4_, M_1_; KNM-LG 1554, dentary with M_2_, M_3_; KNM-SO 8334, dentary with P_4_, M_1_; KNM-SO 1380, dentary with dP_4_, M_1_; KNM-SO, 15976, dentary with M_2_, M_3_; KNM-LG 2398, maxilla with M_1_; KNM-CA 2731, dentary with M_3_; KNM-CA 301, dentary with M_2_, M_3_; KNM-CA 302, dentary fragment with M_3_; KNM-SO 3120, M^2^

***Namasector soriae***

Formation: ?

Locality: Elisabethfeld (Tortoise Site) Sperrgebiet

Geological Age: early Miocene

Absolute Age: 21 Ma – 19 Ma

Country: Namibia

Citation: Morales, Pickford & Salesa, 2008

Specimens observed from Morales, Pickford & Salesa, 2008: Elisabethfeld EF 60’01, dentary with dP_4_, M_1_, M_2_; Elisabethfeld EF 118’04, maxilla fragment with dP^3^, dP^4^, M^1^, premaxilla fragment with canine root

LITERATURE CITED

Barry, J. C. 1988. *Dissopsalis*, a middle and late Miocene proviverrine creodont (Mammalia) from Pakistan and Kenya. Journal of Vertebrate Paleontology 8:25–45.

Beck, R. M. D., and M. S. Y. Lee. 2014. Ancient dates or accelerated rates? Morphological clocks and the antiquity of placental mammals. Proceedings of the Royal Society B 281:20141278.

Becker, D., G. Rauber, and L. Scherler. 2013. New small mammal fauna of late Middle Eocene age from a fissure filling at La Verrerie de Roches (Jura, NW Switzerland). Revue de Paléobiologie, Genéve 32:433–446.

Coster, P., M. Benammi, M. Hahboubi, R. Tabuce, M. Adaci, L. Marivaux, M. Bensalah, S. Mahboubi, A. Mahboubi, F. Mebrouk, C. Maameri, and J-. J. Jaeger. 2012. Chronology of the Eocene continental deposits of Africa: Megnetostratigraphy and biostratigraphy of the El Kohol and Glib Zegdou Formations, Algeria. Geological Society of America Bulletin 124:1590–1606. doi: 10.1130/B30565.1

Chang, S., H. Zhang, P. R. Renne, and Y. Fang. 2009. High-precision ^40^Ar/^39^Ar age for the Jehol Biota. Palaeogeography, Palaeoclimatology, Palzeoecology 280:94–104. doi:10.1016/j.palaeo.2009.06.021

Crochet, J. -Y. 1988. Le plus ancien créodonte africain: *Koholia atlasense* nov. gen., nov. sp. (Eocène inférieur d’El Kohol, Atlas saharien, Algérie). Comptes Rendus de l’Académie des Sciences, Paris, Série II 307:1795–1798.

Crochet, J. -Y., H. Thomas, J. Roger, and S. Al-Sulaimani. 1990. Première découverte d’un créodonte dans la péninsule Arabique: *Masrasector ligabuei* nov. sp. (Oligocène inférieur de Taqah, Formation d’Ashawq, Sultanat d’Oman). Comptes rendus de l’Académie des sciences, Paris, Série II 311:1455–1460.

Egi, N., T. Tsubamoto, and M. Takai. 2007. Systematic status of Asian “*Pterodon*” and early evolution of hyaenaelurine hyaenodontid creodonts. Journal of Paleontology 81:770– 778.

Egi, N., P. A. Holroyd, T. Tsubamoto, A. N. Soe, M. Takai, R. L. Ciochon. 2005. Proviverrine hyaenodontids (Creodonta: Mammalia) from the Eocene of Myanmar and a phylogenetic analysis of the proviverrines from the para-Tethys area. Journal of Systematic Palaeontology 3:337–358.

Fox, R. C. 2015. A revision of the Late Cretaceous-Paleocene eutherian mammal *Cimolestes* Marsh, 1889. Canadian Journal of Earth Sciences 52:1137–1149. dx.doi.org/10.1139/cjes-2015-0113

Gebo, D., and K. Rose. 1993. Skeletal morphology and locomotor adaptation in *Prolimnocyon atavus,* an Early Eocene hyaenodontid creodont. Journal of Vertebrate Paleontology 13:125–144.

Gheerbrant, E., M. Iarochene, M. Amaghzaz, and B. Bouya. 2006. Early African hyaenodontid mammals and their bearing on the origin of Creodonta. Geological Magazine 143(4): 475–489.

Gingerich, P. D., and H. A. Deutsch. 1989. Systematics and evolution of early Eocene Hyaenodontidae (Mammalia, Creodonta) in the Clarks Fork Basin, Wyoming. Contributions from the Museum of Paleontology 27:327–391.

Godinot, M. 1981. Les mammiféres de Rians (Éocéne inférieur, Provence). Palaeovertebrata 10:43–126.

Godinot, M., J. Y. Crochet, J. L. Hartenberger, B. Lange-Badré, D. E. Russell, and B. Sigé. 1987. Nouvelles données sur les mammifères de Palette (Eocène inférieur, Provence). Münchner Geowissenschaftliche Abhandlungen A10:273–288.

Grohé, C., M. Morlo, Y. Chaimanee, C. Blondel, P. Coster, X. Valentin, M. Salem, A. A. Bilal, J. -J. Jaeger, and M. Brunet. 2012. New Apterodontinae (Hyaenodontida) from the Eocene locality of Dur At-Talah (Libya): systematic, paleoecological, and phylogenetical implications. PLOS ONE 7(11):e49054.

Gunnell, G. F. 1998. Creodonta; pp. 91–109 in C. M. Janis, K. M. Scott and L. L. Jacobs (eds.), Evolution of the Tertiary Mammals of North America. Volume 1: Terrestrial carnivores, ungulates, and ungulate like mammals. Cambridge University Press, Cambridge.

Hartenberger, J.-L. 1970. Les mammiferes d'Egerkingen et l'histoire des faunes de l'Eocene d'Europe. Comptes Rendus de la Societe geologique de France 12:886-893.

Holroyd, P. A. 1994. An examination of dispersal origins of Fayum Mammalia. Ph.D. dissertation, Duke University, Durham, 328 pp.

Holroyd, P. A. 1999. New Pterodontinae (Creodonta: Hyaenodontidae) from the late Eocene– early Oligocene Jebel Qatrani Formation, Fayum province, Egypt. PaleoBios 19(2):1–18.

Huang, X.-S., and B.-C. Zhu. 2002. Creodont (Mammalia) remains from the early Oligocene of Uiantatal, Nei Mongol. Vertebrata PalAsiatica 40:21–30.

Ivy, L. 1993. Systematic revision of early to middle Eocene North American Hyaenodontidae (Mammalia, Creodonta). Ph.D. dissertation, University of Colorado at Boulder, 463 pp.

Kielan-Jaworowska, Z., R. L. Cifelli, and Z. -X. Luo. 2004. Mammals from the Age of Dinosaurs. Columbia University Press, New York, 630 pp.

Kocsis, L., E. Gheerbrant, M. Mouflih, H. Capetta, J. Yans, and M. Amaghzaz. 2014. Comprehensive stable isotope investigation of marine biogenic apatite from the late Cretaceous–early Eocene phosphate series of Morocco. Palaeogeography, Palaeoclimatology, Palaeoecology 394:74–78.

Kumar, K. 1992. *Paratritemnodon indicus* (Creodonta: Mammalia) from the early Middle Eocene Subathu Formation, NW Himalaya, India, and the Kalakot mammalian community structure. Paleontologische Zeitschrift 66:387–403.

Lange-Badré, B. 1979. Les créodontes (Mammalia) d’Europe occidentale de l’Éocène supérieur à l’Oligocène supérieur. Mémoires du Muséum National d’Histoire Naturelle 42:1–249.

Lange-Badré, B. 1984. *Hurzelerius torvidus* (Van Valen), nouveau genre de Créodontes (Mammalia) du Bartonien inférieur d’Egerkingen (Suisse) en replacement de *Prototomus torvidus* Van Valen. Comptes Rendus de l’Académie des Sciences, Paris, Série II 299:739-742.

Lange-Badré, B., and M. Böhme. 2005. *Apterodon intermedius*, sp. nov., a new European creodont mammal from MP22 of Espenhain (Germany). Annals de Paléontologie 91:311–328.

Lavrov, A. V. 1996. A new genus *Neoparapterodon* (Creodonta, Hyaenodontidae) from the Khaichin-Ula-2 locality (Khaichin Formation, Middle-Upper Eocene, Mongolia) and the systematic position of the Asiatic *Pterodon* representatives. Paleontological Journal 30:593–604.

Lavrov, A. V. 1999. New material on the Hyaenodontidae (Mammalia, Creodonta) from the Ergiligyn Dzo Formation (Late Eocene of Mongolia) and some notes on the system of the Hyaenodontidae. Paleontological Journal 33:321–329.

Lavrov, A. V. 2007. A new species of *Paroxyaena* (Hyaenodontidae, Creodonta) from Phosphorites of Quercy, Late Eocene, France. Paleontological Journal 41:298–311.

Lewis, M. E., and M. Morlo. 2010. Creodonta; pp. 543–560 in L. Werdelin and W. Sanders (eds.), Cenozoic Mammals of Africa. University of California Press, Berkeley.

Lillegraven, J. A. 1969. Latest Cretaceous mammals of upper part of the Edmonton Formation of Alberta, Canada, and review of marsupial–placental dichotomy in mammalian evolution. Paleontological Contribution of the University of Kansas 50:1–122.

Liu, L.-P., and X.-S. Huang. 2002. *Propterodon* (Hyaenodontidae, Creodonta, Mammalia) from the middle Eocene of Yuanqu Basin, Shanxi Province. Vertebrata PalAsiatica 40:133– 138.

Matthew, W. D. 1901. Additional observations on Creodonta. Bulletin of the American Museum of Natural History 14:1–38.

Matthew, W. D. 1906. The osteology of *Sinopa,* a creodont mammal of the middle Eocene. Proceedings of the United States National Museum 30:203–233.

Matthew, W. D. 1909. The Carnivora and Insectivora of the Bridger Basin, middle Eocene. Memoirs of the American Museum of Natural History 9:289–567.

Matthew, W. D. 1915. A revision of the lower Eocene Wasatch and Wind River faunas, Part 1, Order Ferae (Carnivora), suborder Creodonta. Bulletin of the American Museum of Natural History 34:4–103.

Matthew, W. D., and W. Granger. 1924. New Carnivora from the Tertiary of Mongolia. American Museum Novitates 104:1–9.

Mellett, J. S. 1969. A skull of *Hemipsalodon* (Mammalia, Deltatheridia) from the Clarno Formation of Oregon. American Museum Novitates 387:1–19.

Mellett, J. S. 1977. Paleobiology of North American *Hyaenodon* (Mammalia, Creodonta). Contributions to Vertebrate Evolution 1:1–134.

Meng, J., R. Zhai, and A. R. Wyss. 1998. The late Paleocene Bayan Ulan fauna of inner Mongolia, China; pp. 148–185 in K. C. Beard, M. R. Dawson (eds.), Dawn of the age of mammals in Asia. Bulletin of Carnegie Museum of Natural History, Pittsburgh.

Moore, J. R., G. P. Wilson, M. Sharma, H. R. Hallock, D. R. Braman, and P. R. Renne. 2014. Assessing the relationships of the Hell Creek–Fort Union contact, Cretaceous–Paleogene boundary, and Chicxulub impact ejecta horizon at the Hell Creek Formation lectostratotype, Montana, USA. Geological Society of America Special Papers 503:123– 135. Doi:10.1130/2014.2503(04)

Morales, J., P. Brewer, and M. Pickford. 2010. Carnivores (Creodonta and Carnivora) from the basal middle Miocene of Gebel Zelten, Libya, with a note on a large amphicyonid from the middle Miocene of Ngorora, Kenya. Bulletin of the Tethys Geological Society, Cairo 5:43-54.

Morales, J., M. Pickford, S. Fraile, M.J. Salesa, and D. Soria. 2003. Creodonta and Carnivora from Arrisdrift, early Middle Miocene of Southern Namibia. Memoir of Geological Survey Namibia 19: 177–194.

Morlo, M., and G. F. Gunnell. 2003. Small limnocyonines (Hyaenodontidae, Mammalia) from the Bidgerian middle Eocene of Wyoming: *Thinocyon, Prolimnocyon,* and *Iridodon,* new genus. Contributions from the Museum of Paleontology, The University of Michigan 31:43–78.

Morlo, M., and J. Habersetzer. 1999. The Hyaenodontidae (Creodonta, Mammalia) from the lower Middle Eocene (MP 11) of Messel (Germany) with special remarks on new x-ray methods. Courier Forschungsinstitut Senckenberg 216:31–73.

Morlo, M., and D. Nagel. 2006. New remains of Hyaenodontidae (Creodonta, Mammalia) from the Oligocene of Central Mongolia. Annals de Paléontologie 92:305–321.

Morlo, M., K. Bastl, W. Wenhao, and S. F. K. Schaal. 2014. The first species of *Sinopa* (Hyaenodontida, Mammalia) from outside of North America: implications for the history of the genus in the Eocene of Asia and North America. Palaeontology 57:111–125.

Polly, P. D. 1996. The skeleton of *Gazinocyon vulpeculus* gen. et comb. nov. and the cladistic relationships of Hyaenodontidae (Eutheria, Mammalia). Journal of Vertebrate Paleontology 16:303–319.

Polly, P. D., and B. Lange-Badré. 1993. A new genus *Eurotherium* (Mammalia, Creodonta) in reference to taxonomic problems with some Eocene hyaenodontids from Eurasia. Comptes Rendus de l’Académie des Sciences, Paris, Série II 317:991–996.

Rana, R., K. Kumar, S. Zack, F. Solé, K. Rose, P. Missiaen, L. Singh, A. Sahni, and T. Smith. 2015. Craniodental and postcranial morphology of *Indohyaenodon raoi* from the early Eocene of India, and its implications for ecology, phylogeny, and biogeography of hyaenodontid mammals. Journal of Vertebrate Paleontology DOI: 10.1080/02724634.2015.965308.

Rodrigues, H. G., L. Marivaux, and M. Vianey-Liaud. 2014. Rodent paleocommunities from the Oligocene of Uiantatal (Inner Mongolia, China). PalaeoVertebrate 38:1–11.

Rose, K. D., L. T. Holbrook, R. S. Rana, K. Kumar, K. E. Jones, H. E. Ahrens, P. Missiaen, A. Sahni, and T. Smith. 2014. Early Eocene fossils suggest that the mammalian order Perissodactyla originated in India. Nature Communications 5:5570. doi:10.1038/ncomms6570

Savage, R. J. G. 1965. Fossil mammals of Africa: 19. The Miocene Carnivora of East Africa. Bulletin of the British Museum of Natural History (Geology) 10:239–316.

Savage, R. J. G. 1973. *Megistotherium,* gigantic hyaenodont from Miocene of Gebel Zelten, Libya. Bulletin of the British Museum of Natural History (Geology) 22:483–511.

Seiffert, E. R. 2006. Revised age estimates for the later Paleogene mammal faunas of Egypt and Oman. Proceedings of the National Academy of Sciences, U.S.A. 103:5000–5005.

Seiffert, E. R. 2010. Chronology of Paleogene mammal localities; pp. 19–26 in L. Werdelin and W. Sanders (eds.), Cenozoic Mammals of Africa. University of California Press, Berkeley.

Simons, E. L., and P. D. Gingerich. 1974. New carnivorous mammals from the Oligocene of Egypt. Annals of the Geological Survey of Egypt 4:157–166.

Smith, T., and R. Smith. 2001. The creodonts (Mammalia, Ferae) from the Paleocene–Eocene transition in Belgium (Tienen Formation, MP7). Belgian Journal of Zoology 131:117– 135.

Solé, F., E. Gheerbrant, A. Mbarek, and B. Bouya. 2009. Further evidence of the African antiquity of hyaenodontid (“Creodonta”, Mammalia) evolution. Zoological Journal of the Linnean Society 156:827–846.

Solé, F. 2013. New proviverrine genus from the early Eocene of Europe and the first phylogeny of late Palaeocene–middle Eocene hyaenodontidans (Mammalia). Journal of Systematic Palaeontology 11:375–398.

Solé, F. 2015. New fossils of Hyaenodonta (Mammalia, Placentalia) from the Ypresian an Lutetian of France, and their bearing on the evolution of the Proviverrinae in Southern Europe. Paleontology.

Solé, F., J. Falconnet, and L. Yves. 2014a. New proviverrines (Hyaenodontida) from the early Eocene of Europe; phylogeny and ecological evolution of the Proviverrinae. Zoological Journal of the Linnean Society 171:878–917.

Solé, F., T. Smith, R. Tabuce, and B. Marandat. 2015. New dental elements of the oldest proviverrine mammal, *Parvagula palulae*, from the early Eocene of Southern France support possible African origin of the subfamily. Acta Palaeontologica Polonica doi:http://dx.doi.org/10.4202/app.00146.2014.

Solé, F., J. Lhuillier, M. Adaci, M. Bensalah, M. Mahboubi, and R. Tabuce. 2014b. The hyaenodontidans from the Gour Lazib area (?early Eocene, Algeria): implications concerning the systematics and the origin of the Hyainailourinae and Teratodontinae. Journal of Systematic Paleontology 12:303–322.

Solé, F., E. Amson, M. Borths, D. Vidalenc, M. Morlo, and K. Bastl. 2015*.* A new large hyainailourine from the Bartonian of Europe and its bearings on the evolution and ecology of massive hyaenodonts (Mammalia). PLOS ONE.

Vislobokova, I. A. 2008. The oldest representative of Entelodontoidea (Artiodactyla, Suiformes) from the Middle Eocene of Khaichin Ula II, Mongolia, and some evolutionary features of this superfamily. Paleontological Journal 42:643–654.

Werdelin, L. 2010. Chronology of Neogene Mammal Localities; pp. 27–43 in L. Werdelin and W. Sanders (eds.), Cenozoic Mammals of Africa. University of California Press, Berkeley.

Wible, J. R., G. W. Rougier, M. J. Novacek, and R. J. Asher. 2007. Cretaceous eutherians and Laurasian origin for placental mammals near the K/T boundary. Nature 447:1003–1006.

Wible, J. R., G. W. Rougier, M. J. Novacek, and R. J. Asher. 2009. The eutherian mammal *Maelestes gobiensis* from the late Cretaceous of Mongolia and the phylogeny of Cretaceous Eutheria. Bulletin of the American Museum of Natural History 327:1–123.

Zack, S. P. 2011. New species of the rare early Eocene creodont *Galecyon* and the radiation of the early Hyaenodontidae. Journal of Paleontology 85:315–336.

Zaw, K., S. Meffre, M. Takai, H. Suzuki, C. Burrett, T. Htike, Z. Thein, T. Tsubamoto, N. Egi, and M. Maung. 2014. The oldest anthropoid primates in SE Asia: Evidence from LA- ICP-MS U-Pb zircon age in the Late Middle Eocene Pondaung Formation, Myanmar. Gondwana Research 26:122–131. [doi:10.1016/j.gr.2013.04.007](http://dx.doi.org/10.1016/j.gr.2013.04.007)
