# Supplementary figures and images for: The first hyaenodont from the late Oligocene Nsungwe Formation of Tanzania: Paleoecological insights into the Paleogene-Neogene carnivore transition
Source: PLoS One. 2017 Oct 11;12(10):e0185301. doi: 10.1371/journal.pone.0185301 (PMC5636082; doi:10.1371/journal.pone.0185301)

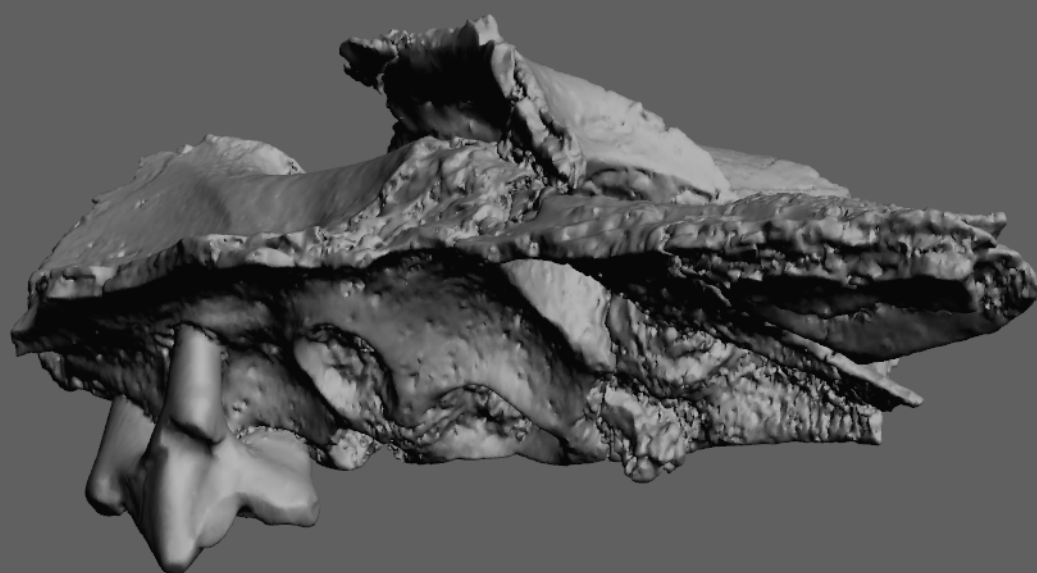

Supplement: S4 Appendix — The holotype of Pakakali rukwaensis embedded in a 3D PDF file. The digital model is also available for download at www.morphosource.org. (PDF) [file pone.0185301.s004.pdf]

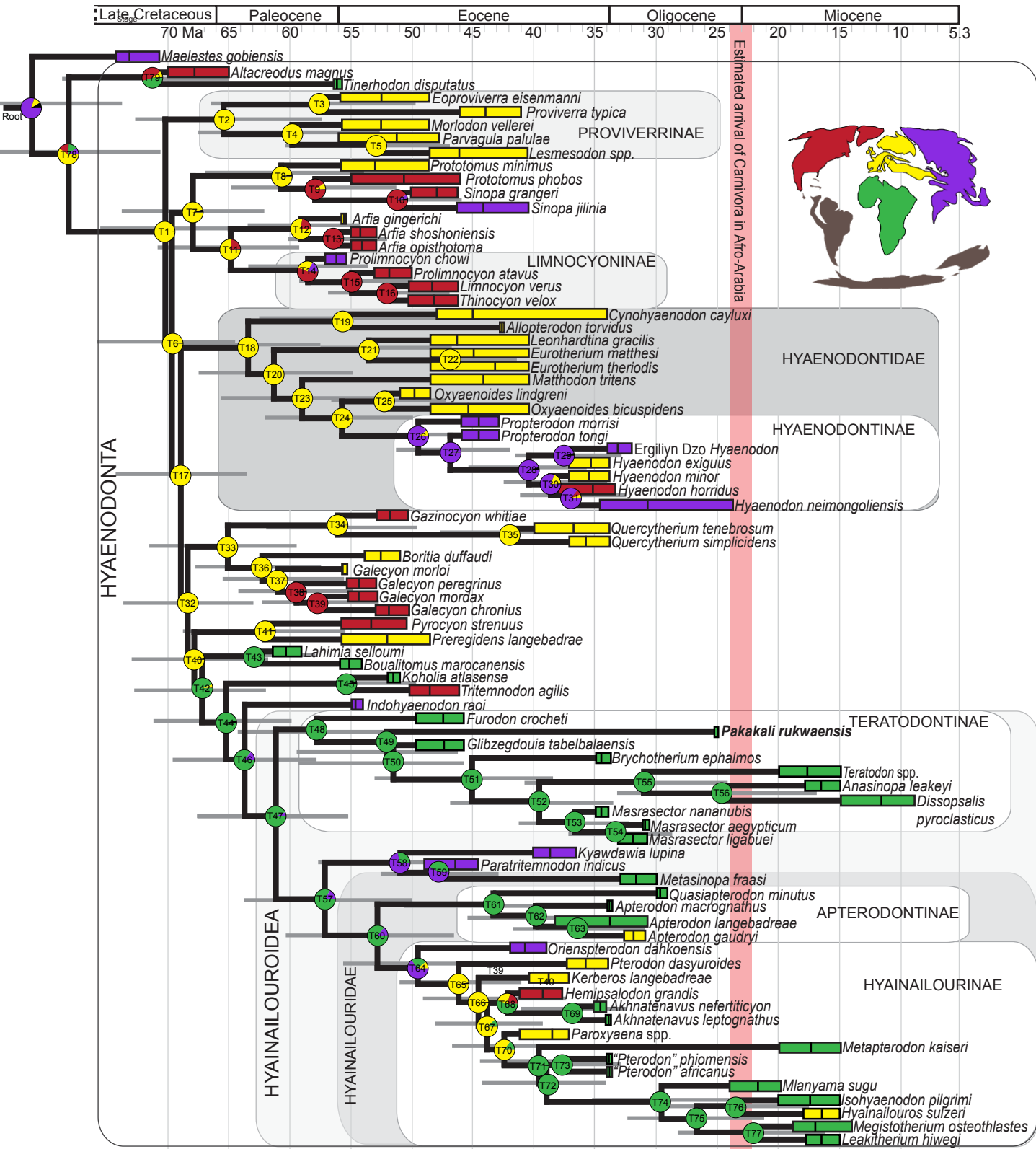

Supplement: S1 Fig — The complete phylogenetic analysis, showing the consensus with all OTUs, rather than only the Afro-Arabian OTUs shown in Fig 4 with BBM biogeographic results over each node. (PDF) [file pone.0185301.s009.pdf]
